# Stability Analysis of a Four-Species Periodic Diffusive Predator–Prey System with Delay and Feedback Control

**DOI:** 10.3390/biology14050462

**Published:** 2025-04-24

**Authors:** Lili Jia, Changyou Wang

**Affiliations:** 1 Department of Basic Teaching, Dianchi College, Kunming 650228, China; lilijiadianchi@163.com; 2 School of Mathematical Sciences, V.C. & V.R. Key Lab of Sichuan Province, Sichuan Normal University, Chengdu 610066, China; 3 College of Applied Mathematics, Chengdu University of Information Technology, Chengdu 610225, China

**Keywords:** delay, feedback control, reaction–diffusion, periodic solution, stability

## Abstract

In this work, we present a novel four-species periodic diffusive predator–prey model, which incorporates delay and feedback control mechanisms, marking substantial progress in ecological modeling. This model offers a more realistic and detailed portrayal of the intricate dynamics of predator–prey interactions. Our primary objective is to establish the existence of a periodic solution for this new model, which depends only on time variables and is independent of spatial variables (we refer to it as a spatially homogeneous periodic solution). By employing the comparison theorem and the fixed point theorem tailored for delay differential equations, we derive a set of sufficient conditions that guarantee the emergence of such a solution. This analytical framework lays a solid mathematical foundation for understanding the periodic behaviors exhibited by predator–prey systems with delayed and feedback-regulated interactions. Moreover, we explore the global asymptotic stability of the aforementioned periodic solution. We organically combine Lyapunov stability theory, upper and lower solution techniques for partial differential equations with delay, and the squeezing theorem for limits to formulate additional sufficient conditions that ensure the stability of the periodic solution. This stability analysis is vital for forecasting the long-term outcomes of predator–prey interactions and evaluating the model’s resilience against disturbances. To validate our theoretical findings, we undertake a series of numerical simulations. These simulations not only corroborate our analytical results but also further elucidate the dynamic behaviors of the four-species predator–prey model. Our research enhances our understanding of the complex interactions within ecological systems and carries significant implications for the conservation and management of biological populations.

## Simple Summary

This study explores interactions among four species (two predators and two prey) in ecosystems using a novel model that incorporates time delays and feedback mechanisms to better reflect real-world dynamics. We aimed to determine whether these populations form consistent, repeating patterns over time and whether these patterns remain stable. By applying mathematical methods such as Lyapunov stability theory and computer simulations, we found that under specific conditions, these regular cycles emerge and persist. The findings improve our understanding of natural ecosystem fluctuations and provide tools for wildlife conservation, helping to predict population changes and guide management decisions to protect biodiversity.

## 1. Introduction

In the vast field of ecological research, understanding the interactions between biological species and their dynamic changes has always been one of the core issues of concern for scientists and researchers. As scientific research progresses, people have gradually realized that while the traditional Lotka–Volterra model provides a valuable initial framework for understanding the dynamic changes in population numbers in predator–prey models, it has limitations in describing the fine structure and dynamic changes in real-world ecosystems. In particular, the limitations of traditional models become particularly evident when considering ratio-dependent population growth rates, time delay effects, and disturbances to ecosystems caused by environmental changes. Therefore, extended models of the classic Lotka–Volterra predator–prey model and their dynamic behaviors have become hot topics in ecological research.

In 2006, Zhang and Teng [1] explored a two-species Lotka–Volterra predator–prey model with periodic coefficients and derived a set of concise criteria to ensure the existence and stability of positive periodic solutions for the model. Subsequently, in 2008, Shi and Chen [2] further constructed a two–species predator–prey model incorporating stage structure and obtained sufficient conditions for the global asymptotic stability of nontrivial periodic solutions. In 2010, Wang [3] focused on the persistence of a periodic predator–prey model with prey diffusion between two patches and derived a criterion to ensure the model’s persistence. In 2013, Kim and Baek [4] introduced impulsive control into the predator–prey model, obtaining the persistence of the model and the existence of nontrivial periodic solutions. As time went on, research on predator–prey models gradually deepened. In 2016, Zhang and Teng [5] considered a periodic predator–prey model with a Gompertz growth function and derived sufficient conditions for the persistence of prey populations and the extinction of predator populations. In 2019, Deng et al. [6] incorporated predator cannibalism into the predator–prey model, obtaining the existence and global stability of equilibrium points for the improved model. The study of predator–prey models has grown increasingly diversified in recent times. In 2020, Zhang et al. [7] studied a Lotka–Volterra predator–prey model with non-selective predation, obtaining sufficient conditions for the persistence of both populations and the global asymptotic stability of positive equilibrium points using Lyapunov stability theory. In 2021, Kaushik and Banerjee [8] considered a predator–prey model incorporating stage structure and counter-attack behavior in predators. In 2022, He and Li [9] focused on a predator–prey model with fear effects, incorporating mutual interference or group defense. However, as model complexity increases, such as by incorporating diffusion terms to more accurately describe interactions between populations, traditional research methods have become increasingly difficult to apply. In 2023, Guin et al. [10] studied a reaction–diffusion predator–prey model with Crowley–Martin type response function, obtaining criteria to ensure the local stability of the trivial solutions and the existence of Hopf bifurcation for the model. In 2024, Liu [11] studied a predator–prey model with a general functional response function and stochastic switching mechanisms, proving that the system has exactly two dynamical bifurcation points. The aforementioned literature initially focused on models considering only periodic coefficients in two-species Lotka–Volterra systems but gradually incorporated complex factors such as impulsive control, Gompertz growth functions, predator cannibalism, fear effects, diffusion terms, and stochastic switching mechanisms over time. As model complexity increases, incorporating more ecological factors and more detailed population dynamics descriptions typically enhances model accuracy. However, more complex models may require more data and more powerful computing capabilities, which can be challenging to achieve in practice. Therefore, how to reduce model complexity and make it easier to apply while ensuring greater accuracy is a difficult problem facing current research.

In recent years, with the in-depth study of non-autonomous systems, ratio-dependent theory, reaction–diffusion processes, and time delay effects, ecologists and mathematicians have begun to construct more sophisticated models to simulate and predict the interactions among multiple populations. With the help of delayed reaction–diffusion equations (DRDEs), numerous real-world phenomena in nature can be accurately described and reasonably explained. The study of DRDEs has gradually become a hot topic in academia. Although early research focused mostly on academic theoretical aspects [12,13], in recent years, numerous outstanding achievements have emerged in the field of periodic solutions and equilibrium points of ratio-dependent predator–prey models with time delays. In 2016, Li [14] explored a ratio-dependent predator–prey model incorporating hyperbolic mortality rates and deeply analyzed the impact mechanism of time delays on the stability of equilibrium solutions of the model. In 2017, Zhang and Li [15] studied a ratio-dependent predator–prey model involving nonlinear predation and hyperbolic mortality rates and successfully derived global stability conditions for the model’s unique constant positive equilibrium point. In 2018, Ma et al. [16] focused on a ratio-dependent predator–prey model with mutual interference among predators and discussed in detail the spatio-temporal dynamics induced by both time delays and diffusion in the model. In 2019, Chen et al. [17] focused their research on a ratio-dependent predator–prey model with Neumann boundary conditions and systematically analyzed the global stability of the model’s strictly positive steady-state solutions. In 2020, Jiang et al. [18] conducted in-depth research on a predator–prey model incorporating ratio-dependent functions and provided stability criteria for the model’s positive equilibrium points. In 2021, Djilali and Bentout [19] further explored a ratio-dependent predator–prey model considering prey social behavior and predator competition and proposed a series of criteria to ensure the stability of inhomogeneous and homogeneous positive periodic solutions of the system. In 2022, Xu et al. [20] focused their research on a ratio-dependent predator–prey model with predator maturation delay and successfully obtained the global asymptotic stability of the model’s positive equilibrium solutions. In 2023, Yuan and Guo [21] conducted in-depth discussions on a spatially nonlocal ratio-dependent model and provided stability criteria for the model’s positive steady-state solutions. In 2024, Ma and Meng [22] studied a ratio-dependent model incorporating memory delays and derived sufficient conditions to ensure the global asymptotic stability of the model’s constant equilibrium solutions.

In actual situations, ecosystems frequently undergo disruptions caused by unforeseeable factors that influence crucial parameters like birth and death rates. Introducing feedback control mechanisms allows for real-time monitoring and adjustment of ecosystem dynamics, enabling the parameters to be tuned based on the system’s current state and future trends to ensure ecosystem stability. In recent years, ecosystems incorporating feedback control have garnered significant attention from many scholars [23,24,25,26,27,28,29,30,31,32]. In 2022, Wang et al. [33] studied a non-autonomous ratio-dependent food chain model incorporating delay and feedback control. Using delayed differential inequalities and Lyapunov methods, they derived a set of sufficient conditions to ensure the persistence and attractivity for positive periodic solution to the model. In 2023, Jiang et al. [31] investigated a n-species Lotka–Volterra mutualism model with feedback control and continuous delay. By utilizing integral inequalities, comparison principles, and Lyapunov methods, they derived sufficient conditions for the model’s global attractiveness, persistence, and the existence of periodic solutions. In 2024, Yue et al. [34] proposed a novel non-autonomous single-species feedback control model based on the feedback control variable related to the COVID-19 pandemic. This model reflects the impact of the pandemic by reducing birth rates and increasing death rates and clarifies sufficient conditions of persistence, extinction, and global stability. This model originates from research on the fear effect in predator species [35,36,37,38]. All the aforementioned models incorporating feedback control belong to the category of ordinary differential equation models. There has been relatively little research on predator models incorporating feedback control in the context of reaction–diffusion equations, and no relevant results have been published to date.

The work presented herein primarily concerns the stability analysis of four-species periodic reaction–diffusion predator–prey models that incorporate time delays and feedback control mechanisms. The exact formulation of these models is detailed below.

(1) $$\left\{\begin{array}{ll} \frac{\partial v_{1}(x,t)}{\partial t}-d_{1}(t)\Delta v_{1}(x,t) & =v_{1}(x,t)[r_{1}(t)-a_{11}(t)v_{1}(x,t-\tau_{11})-a_{12}(t)v_{2}(x,t-\tau_{12})-\frac{a_{13}(t)v_{3}(x,t)}{b_{13}(t)v_{3}(x,t)+v_{1}(x,t)}-\frac{a_{14}(t)v_{4}(x,t)}{b_{14}(t)v_{4}(x,t)+v_{1}(x,t)}-k_{1}(t)u_{1}(x,t)],\\ \frac{\partial v_{2}(x,t)}{\partial t}-d_{2}(t)\Delta v_{2}(x,t) & =v_{2}(x,t)[r_{2}(t)-a_{22}(t)v_{2}(x,t-\tau_{22})-a_{21}(t)v_{1}(x,t-\tau_{21})-\frac{a_{23}(t)v_{3}(x,t)}{b_{23}(t)v_{3}(x,t)+v_{2}(x,t)}-\frac{a_{24}(t)v_{4}(x,t)}{b_{24}(t)v_{4}(x,t)+v_{2}(x,t)}-k_{2}(t)u_{2}(x,t)],\\ \frac{\partial v_{3}(x,t)}{\partial t}-d_{3}(t)\Delta v_{3}(x,t) & =v_{3}(x,t)[-r_{3}(t)+\frac{a_{31}(t)v_{1}(x,t-\tau_{31})}{b_{13}(t)v_{3}(x,t)+v_{1}(x,t-\tau_{31})}+\frac{a_{32}(t)v_{2}(x,t-\tau_{32})}{b_{23}(t)v_{3}(x,t)+v_{2}(x,t-\tau_{32})}-a_{34}(t)v_{4}(x,t-\tau_{34})+k_{3}(t)u_{3}(x,t)],\\ \frac{\partial v_{4}(x,t)}{\partial t}-d_{4}(t)\Delta v_{4}(x,t) & =v_{4}(x,t)[-r_{4}(t)+\frac{a_{41}(t)v_{1}(x,t-\tau_{41})}{b_{14}(t)v_{4}(x,t)+v_{1}(x,t-\tau_{41})}+\frac{a_{42}(t)v_{2}(x,t-\tau_{42})}{b_{24}(t)v_{4}(x,t)+v_{2}(x,t-\tau_{42})}-a_{43}(t)v_{3}(x,t-\tau_{43})+k_{4}(t)u_{4}(x,t)],\\ \frac{\partial u_{1}(x,t)}{\partial t} & =e_{1}(t)-f_{1}(t)u_{1}(x,t)+q_{1}(t)v_{1}(x,t),\;\frac{\partial u_{2}(x,t)}{\partial t}=e_{2}(t)-f_{2}(t)u_{2}(x,t)+q_{2}(t)v_{2}(x,t),\\ \frac{\partial u_{3}(x,t)}{\partial t} & =e_{3}(t)-f_{3}(t)u_{3}(x,t)-q_{3}(t)v_{3}(x,t),\;\frac{\partial u_{4}(x,t)}{\partial t}=e_{4}(t)-f_{4}(t)u_{4}(x,t)-q_{4}(t)v_{4}(x,t), \end{array}\right.$$

with the following boundary and initial conditions:

(2) $$\left\{\begin{array}{l} \partial v_{i}(x,t)/\partial n=\partial u_{i}(x,t)/\partial n=0,\;(x,t)\in \partial \Omega \times R^{+},\;i=1,2,3,4,\\ v_{i}(x,t)=\eta_{i0}(x,t)>0,\;x\in \Omega \times (-\tau ,0),\;u_{i}(x,0)=\mu_{i0}(x)>0,x\in \Omega , \end{array}\right.$$

where Ω is a smooth bounded domain in $R^{n}$ with boundary, ∂Ω,Δ is a Laplace operator on Ω, ∂/∂n represents the outward normal derivation on $\partial \Omega ,\;v_{i}(x,t)$ denotes the density of i-th species at point $x=(x_{1},x_{2},\cdots ,x_{n})$ and the time of t. $u_{i}(t)$ are the feedback control functions, and $k_{i}(t),e_{i}(t),f_{i}(t)$ and $q_{i}(t)$ are the control parameters. $\tau_{11},\tau_{12},\tau_{21},\tau_{22}$ are constants, which represent the negative feedback delay caused by the crowding of prey and predators, respectively. $\tau_{31},\;\tau_{32},\tau_{41},\tau_{42}$ are constant delays due to pregnancy, that is, only mature adult predators can contribute to the predator’s biomass. And $\tau =\max\left\{\tau_{11},\tau_{12},\tau_{21},\tau_{22},\;\tau_{31},\tau_{32},\tau_{41},\tau_{42}\right\}$. From Table 1, one can discern the biological implications associated with the other parameters in model (1.1). All coefficients in the model are positive ω-periodic functions that are both continuous and bounded within the interval [0, +∞). This model integrates multiple key factors to more accurately reflect the complexity and dynamics of predation activities in nature, thereby furnishing a scientific basis for ecological protection, biodiversity management, and sustainable resource utilization.

The article is structured as follows: Section 2 explores the existence of space homogeneous periodic solution in reaction–diffusion predator–prey models incorporating time delays and feedback controls. Section 3 shifts attention to the global asymptotic stability of the space homogeneous periodic solution. Section 4 offers a numerical illustration to support the theoretical results presented herein. Lastly, the conclusion summarizes the primary achievements of our research.

The main contributions of this paper are as follows: (1) Innovation in model construction: The paper introduces a groundbreaking periodic reaction–diffusion system that seamlessly integrates time delays and feedback control. This novel system transcends traditional predator–prey models by amalgamating multiple pivotal ecological factors: the influence of time delays, the intricacies of feedback control mechanisms, non-autonomous behaviors, ratio-dependent functions, and the essential dynamics of reaction–diffusion processes. This holistic integration enables the model to more accurately mirror the complex population fluctuations observed in real-world ecosystems, offering a significant advancement in our understanding of ecological dynamics. (2) Innovative Research Perspective: By delving into the existence and stability of time-periodic solutions within this innovative model, the research presents a fresh lens through which to view the dynamic responses of ecosystems to periodic environmental perturbations. Such perturbations, like seasonal variations and climatic shifts, are ubiquitous in nature. This study provides profound insights into how these cyclic changes impact the stability and behavior of ecological systems, thereby enriching our ecological knowledge and enhancing our predictive capabilities concerning ecosystem responses to environmental alterations. (3) Innovative Research Methodology: The methodology employed in this paper represents a remarkable transformation, cleverly reducing the stability problem of reaction–diffusion ecosystems with time delays and feedback control to the stability analysis of corresponding ordinary differential ecosystems sharing similar characteristics. This ingenious approach not only streamlines the complexity of the problem but also opens up a novel perspective for exploring and comprehending the stability dynamics of such ecosystems. This methodological innovation stands as a testament to the paper’s contribution to the field, offering a simpler yet equally effective framework for future research endeavors in this crucial area of biomathematics.

## 2. Existence of Space Homogeneous Periodic Solution

Set φ(t) be a positive ω-periodic continuous and bounded functions defined on $R_{+}$, we denote

$$\varphi^{m}=\sup\left\{\varphi (t),\;t\in R_{+}\right\},\;\varphi^{l}=\inf\left\{\varphi (t),\;t\in R_{+}\right\}$$

Next, we will consider the degenerate system of the four-species periodic predator–prey diffusive system (1) and (2), which is described as follows:

(3) $$\left\{\begin{array}{l} \frac{dv_{1}(t)}{dt}=v_{1}(t)[r_{1}(t)-a_{11}(t)v_{1}(t-\tau_{11})-a_{12}(t)v_{2}(t-\tau_{12})-\frac{a_{13}(t)v_{3}(t)}{b_{13}(t)v_{3}(t)+v_{1}(t)}-\frac{a_{14}(t)v_{4}(t)}{b_{14}(t)v_{4}(t)+v_{1}(t)}-k_{1}(t)u_{1}(t)],\\ \frac{dv_{2}(t)}{dt}=v_{2}(t)[r_{2}(t)-a_{22}(t)v_{2}(t-\tau_{22})-a_{21}(t)v_{1}(t-\tau_{21})-\frac{a_{23}(t)v_{3}(t)}{b_{23}(t)v_{3}(t)+v_{2}(t)}-\frac{a_{24}(t)v_{4}(t)}{b_{24}(t)v_{4}(t)+v_{2}(t)}-k_{2}(t)u_{2}(t)],\\ \frac{dv_{3}(t)}{dt}=v_{3}(t)[-r_{3}(t)+\frac{a_{31}(t)v_{1}(t-\tau_{31})}{b_{13}(t)v_{3}(t)+v_{1}(t-\tau_{31})}+\frac{a_{32}(t)v_{2}(t-\tau_{32})}{b_{23}(t)v_{3}(t)+v_{2}(t-\tau_{32})}-a_{34}(t)v_{4}(t-\tau_{34})+k_{3}(t)u_{3}(t)],\\ \frac{dv_{4}(t)}{dt}=v_{4}(t)[-r_{4}(t)+\frac{a_{41}(t)v_{1}(t-\tau_{41})}{b_{14}(t)v_{4}(t)+v_{1}(t-\tau_{41})}+\frac{a_{42}(t)v_{2}(t-\tau_{42})}{b_{24}(t)v_{4}(t)+v_{2}(t-\tau_{42})}-a_{43}(t)v_{3}(t-\tau_{43})+k_{4}(t)u_{4}(t)],\\ \frac{du_{1}(t)}{dt}=e_{1}(t)-f_{1}(t)u_{1}(t)+q_{1}(t)v_{1}(t),\;\frac{du_{2}(t)}{dt}=e_{2}(t)-f_{2}(t)u_{2}(t)+q_{2}(t)v_{2}(t),\\ \frac{du_{3}(t)}{dt}=e_{3}(t)-f_{3}(t)u_{3}(t)-q_{3}(t)v_{3}(t),\;\frac{du_{4}(t)}{dt}=e_{4}(t)-f_{4}(t)u_{4}(t)-q_{4}(t)v_{4}(t), \end{array}\right.$$

with the initial conditions

(4) $$\;v_{i}(t)=\xi_{i}(t)>0,\;u_{i}(t)=\eta_{i}(t)>0,t\in [-\tau ,\;0].$$

**Theorem 1.**  *For any positive initial conditions, the solution of the models (3) and (4) is positive.*

**Proof.**  It can be proven using a proof method similar to Theorem 2.1 in reference [39], which is omitted here.

For the system (3), set

$$\begin{array}{c} M_{1}=\frac{r_{1}^{m}}{a_{11}^{l}}\exp(r_{1}^{m}\tau_{11}),\;M_{2}=\frac{r_{2}^{m}}{a_{22}^{l}}\exp(r_{2}^{m}\tau_{22}),\\ N_{1}=\frac{e_{1}^{m}+q_{1}^{m}M_{1}}{f_{1}^{l}},\;N_{2}=\frac{e_{2}^{m}+q_{2}^{m}M_{2}}{f_{2}^{l}},\;N_{3}=\frac{e_{3}^{m}}{f_{3}^{l}},\;N_{4}=\frac{e_{4}^{m}}{f_{4}^{l}},\\ n_{1}=\frac{e_{1}^{l}+q_{1}^{l}m_{1}}{f_{1}^{m}},\;n_{2}=\frac{e_{2}^{l}+q_{2}^{l}m_{2}}{f_{2}^{m}},\;n_{3}=\frac{e_{3}^{l}-q_{3}^{m}M_{3}}{f_{3}^{m}},\;n_{4}=\frac{e_{4}^{l}-q_{4}^{m}M_{4}}{f_{4}^{m}},\\ M_{3}^{*}=\frac{M_{1}(a_{31}^{m}-r_{3}^{l}+a_{32}^{m}+k_{3}^{m}N_{3})}{b_{13}^{l}(r_{3}^{l}-a_{32}^{m}-k_{3}^{m}N_{3})},\;M_{4}^{*}=\frac{M_{1}(a_{41}^{m}-r_{4}^{l}+a_{42}^{m}+k_{4}^{m}N_{4})}{b_{14}^{l}(r_{4}^{l}-a_{42}^{m}-k_{4}^{m}N_{4})},\\ m_{1}=\frac{(r_{1}^{l}-a_{12}^{m}M_{2}-k_{1}^{m}N_{1})b_{13}^{l}b_{14}^{l}-a_{13}^{m}b_{14}^{l}-a_{14}^{m}b_{13}^{l}}{b_{13}^{l}b_{14}^{l}a_{11}^{m}}\exp[(\frac{(r_{1}^{l}-a_{12}^{m}M_{2}-k_{1}^{m}N_{1})b_{13}^{l}b_{14}^{l}-a_{13}^{m}b_{14}^{l}-a_{14}^{m}b_{13}^{l}}{b_{13}^{l}b_{14}^{l}}-a_{11}^{m}M_{1})\tau_{11}],\\ m_{2}=\frac{(r_{2}^{l}-a_{21}^{m}M_{1}-k_{2}^{m}N_{2})b_{23}^{l}b_{24}^{l}-a_{23}^{m}b_{24}^{l}-a_{24}^{m}b_{23}^{l}}{b_{23}^{l}b_{24}^{l}a_{22}^{m}}\exp[(\frac{(r_{2}^{l}-a_{21}^{m}M_{1}-k_{2}^{m}N_{2})b_{23}^{l}b_{24}^{l}-a_{23}^{m}b_{24}^{l}-a_{24}^{m}b_{23}^{l}}{b_{23}^{l}b_{24}^{l}}-a_{22}^{m}M_{2})\tau_{22}],\\ m_{3}^{*}=\frac{a_{31}^{l}m_{1}-M_{1}(r_{3}^{m}+a_{34}^{m}M_{4}-k_{3}^{l}n_{3})}{b_{13}^{m}(r_{3}^{m}+a_{34}^{m}M_{4}-k_{3}^{l}n_{3})},\;m_{4}^{*}=\frac{a_{41}^{l}m_{1}-M_{1}(r_{4}^{m}+a_{43}^{m}M_{3}-k_{4}^{l}n_{4})}{b_{14}^{m}(r_{4}^{m}+a_{43}^{m}M_{3}-k_{4}^{l}n_{4})}. \end{array}$$

□

**Theorem 2.**  *Suppose that*

$$\begin{array}{l} (H_{1})\;r_{3}^{l}-a_{32}^{m}-k_{3}^{m}N_{3}>0,\;(H_{2})\;r_{4}^{l}-a_{42}^{m}-k_{4}^{m}N_{4}>0,\\ (H_{3})\;a_{31}^{m}-r_{3}^{l}+a_{32}^{m}+k_{3}^{m}N_{3}>0,\;(H_{4})\;a_{41}^{m}-r_{4}^{l}+a_{42}^{m}+k_{4}^{m}N_{4}>0,\\ (H_{5})\;(r_{1}^{l}-a_{12}^{m}M_{2}-k_{1}^{m}N_{1})b_{13}^{l}b_{14}^{l}-a_{13}^{m}b_{14}^{l}-a_{14}^{m}b_{13}^{l}>0,\;\\ (H_{6})\;(r_{2}^{l}-a_{21}^{m}M_{1}-k_{2}^{m}N_{2})b_{23}^{l}b_{24}^{l}-a_{23}^{m}b_{24}^{l}-a_{24}^{m}b_{23}^{l}>0,\\ (H_{7})\;r_{3}^{m}+a_{34}^{m}M_{4}-k_{3}^{l}n_{3}>0,\;(H_{8})\;r_{4}^{m}+a_{43}^{m}M_{3}-k_{4}^{l}n_{4}>0,\\ (H_{9})\;a_{31}^{l}m_{1}-M_{1}(r_{3}^{m}+a_{34}^{m}M_{4}-k_{3}^{l}n_{3})>0,\;\\ (H_{10})\;a_{41}^{l}m_{1}-M_{1}(r_{4}^{m}+a_{43}^{m}M_{3}-k_{4}^{l}n_{4})>0. \end{array}$$

Then, the system (3) is permanent.

**Proof.**  Under fulfillment of conditions $\left(H_{1})-(H_{6}\right)$ by model (3), there exists some appropriate positive real numbers $M_{i},\;m_{i},\;(i=3,4)$ enabling

(5) $$0<m_{i}<m_{i}^{*}<M_{i}^{*}<M_{i}.$$

From the primary equation of system (3), we obtain

$$\begin{array}{ll} \frac{dv_{1}(t)}{dt} & =v_{1}(t)[r_{1}(t)-a_{11}(t)v_{1}(t-\tau_{11})-a_{12}(t)v_{2}(t-\tau_{12})-\frac{a_{13}(t)v_{3}(t)}{b_{13}(t)v_{3}(t)+v_{1}(t)}-\frac{a_{14}(t)v_{4}(t)}{b_{14}(t)v_{4}(t)+v_{1}(t)}-k_{1}(t)u_{1}(t)]\\ & \leq v_{1}(t)[r_{1}(t)-a_{11}(t)v_{1}(t-\tau_{11})]\leq v_{1}(t)[r_{1}^{m}-a_{11}^{l}v_{1}(t-\tau_{11})]. \end{array}$$

From the Lemma 2.2 in [40], it follows that

(6) $$\underset{t\to +\infty }{\lim\sup\;}v_{1}(t)\leq \frac{r_{1}^{m}}{a_{11}^{l}}\exp(r_{1}^{m}\tau_{11})=M_{1}.$$

Similarly, from the second equation of system (3), we derive

(7) $$\underset{t\to +\infty }{\lim\sup\;}v_{2}(t)\leq \frac{r_{2}^{m}}{a_{22}^{l}}\exp(r_{2}^{m}\tau_{22})=M_{2}.$$

From the fifth equation of system (3), we conclude that

$$\frac{du_{1}(t)}{dt}=e_{1}(t)-f_{1}(t)u_{1}(t)+q_{1}(t)v_{1}(t)\leq e_{1}^{m}-f_{1}^{l}u_{1}(t)+q_{1}^{m}M_{1},$$

From the Lemma 2.1 in [33], it follows that

(8) $$\underset{t\to +\infty }{\lim\sup\;}u_{1}(t)\leq \frac{e_{1}^{m}+q_{1}^{m}M_{1}}{f_{1}^{l}}=N_{1}.$$

Similarly, from the sixth equation of system (3) and the Lemma 2.1 in [33], it holds that

(9) $$\underset{t\to +\infty }{\lim\sup\;}u_{2}(t)\leq \frac{e_{2}^{m}+q_{2}^{m}M_{2}}{f_{2}^{l}}=N_{2}.$$

According to the seventh and eighth equations of system (3), we obtain

$$\frac{du_{3}(t)}{dt}=e_{3}(t)-f_{3}(t)u_{3}(t)-q_{3}(t)v_{3}(t)\leq e_{3}(t)-f_{3}(t)u_{3}(t)\leq e_{3}^{m}-f_{3}^{l}u_{3}(t),$$

and

$$\frac{du_{4}(t)}{dt}=e_{4}(t)-f_{4}(t)u_{4}(t)-q_{4}(t)v_{4}(t)\leq e_{4}(t)-f_{4}(t)u_{4}(t)\leq e_{4}^{m}-f_{4}^{l}u_{4}(t),$$

From the Lemma 2.1 in [33], it follows that

(10) $$\underset{t\to +\infty }{\lim\sup\;}u_{3}(t)\leq \frac{e_{3}^{m}}{f_{3}^{l}}=N_{3},$$

and

(11) $$\underset{t\to +\infty }{\lim\sup\;}u_{4}(t)\leq \frac{e_{4}^{m}}{f_{4}^{l}}=N_{4}.$$

According to the third of system (3), and combining (5), (6), (10), and $H_{1}$, we have

$$\begin{array}{ll} \frac{dv_{3}(t)}{dt} & =v_{3}(t)[-r_{3}(t)+\frac{a_{31}(t)v_{1}(t-\tau_{31})}{b_{13}(t)v_{3}(t)+v_{1}(t-\tau_{31})}+\frac{a_{32}(t)v_{2}(t-\tau_{32})}{b_{23}(t)v_{3}(t)+v_{2}(t-\tau_{32})}-a_{34}(t)v_{4}(t-\tau_{34})+k_{3}(t)u_{3}(t)]\\ & \leq v_{3}(t)[-r_{3}(t)+\frac{a_{31}(t)v_{1}(t-\tau_{31})}{b_{13}(t)v_{3}(t)+v_{1}(t-\tau_{31})}+\frac{a_{32}(t)v_{2}(t-\tau_{32})}{b_{23}(t)v_{3}(t)+v_{2}(t-\tau_{32})}+k_{3}(t)u_{3}(t)]\\ & \leq v_{3}(t)[-r_{3}^{l}+\frac{a_{31}^{m}M_{1}}{b_{13}^{l}v_{3}(t)+M_{1}}+a_{32}^{m}+k_{3}^{m}N_{3}]\\ & =v_{3}(t)\frac{(-r_{3}^{l}+a_{32}^{m}+k_{3}^{m}N_{3})(b_{13}^{l}v_{3}(t)+M_{1})+a_{31}^{m}M_{1}}{b_{13}^{l}v_{3}(t)+M_{1}}\\ & =v_{3}(t)\frac{b_{13}^{l}(r_{3}^{l}-a_{32}^{m}-k_{3}^{m}N_{3})}{b_{13}^{l}v_{3}(t)+M_{1}}[-v_{3}(t)-\frac{M_{1}}{b_{13}^{l}}+\frac{a_{31}^{m}M_{1}}{b_{13}^{l}(r_{3}^{l}-a_{32}^{m}-k_{3}^{m}N_{3})}]\\ & =v_{3}(t)\frac{b_{13}^{l}(r_{3}^{l}-a_{32}^{m}-k_{3}^{m}N_{3})}{b_{13}^{l}v_{3}(t)+M_{1}}[-v_{3}(t)+\frac{M_{1}(a_{31}^{m}-r_{3}^{l}+a_{32}^{m}+k_{3}^{m}N_{3})}{b_{13}^{l}(r_{3}^{l}-a_{32}^{m}-k_{3}^{m}N_{3})}]\\ & =v_{3}(t)\frac{b_{13}^{l}(r_{3}^{l}-a_{32}^{m}-k_{3}^{m}N_{3})}{b_{13}^{l}v_{3}(t)+M_{1}}[-v_{3}(t)+M_{3}^{*}]<v_{3}(t)\frac{b_{13}^{l}(r_{3}^{l}-a_{32}^{m}-k_{3}^{m}N_{3})}{M_{1}}[-v_{3}(t)+M_{3}]. \end{array}$$

In view of the comparison theorem of the ordinary differential equation and $H_{3}$, we can obtain that

1. When $0<v_{3}(t_{0})<M_{3}$, if $t\geq t_{0}$, then $v_{3}(t)\leq M_{3}$.
2. When $v_{3}(t_{0})\geq M_{3}$, for a sufficiently large t, one has $v_{3}(t)\leq M_{3}$. Otherwise, if $v_{3}(t)>M_{3}$, then there exists α>0 such that $v_{3}(t)\geq M_{3}^{*}+\alpha$. Furthermore, one has
3.
${{\dot{v}}_{3}(t)|}_{v_{3}(t)>M_{3}}\leq \frac{b_{13}^{l}(r_{3}^{l}-a_{32}^{m}-k_{3}^{m}N_{3})}{M_{1}}v_{3}(t)[-v_{3}(t)+M_{3}^{*}]<-\frac{b_{13}^{l}(r_{3}^{l}-a_{32}^{m}-k_{3}^{m}N_{3})}{M_{1}}\alpha v_{3}(t),$

4. Thus, from $\left(H_{1}\right)$, it holds that

(12) $$v_{3}(t)<v_{3}(t_{0})\exp(-\frac{b_{13}^{l}(r_{3}^{l}-a_{32}^{m}-k_{3}^{m}N_{3})}{M_{1}}\alpha \;t)\to 0\;\mathrm{as}\;t\to +\infty$$

It is obvious that the inequality (12) contradicts $v_{3}(t)>M_{3}$. Therefore, we can select an adequately large $T_{3}\geq t_{0}\geq 0$ such that

(13) $$v_{3}(t)\leq M_{3}\;\mathrm{as}\;t>T_{3}$$

Similarly, from the fourth equation of the model (3), and combining (5), (6), (11), $H_{2}$,and $H_{4}$, there exists a sufficiently large $T_{4}\geq t_{0}\geq 0$, enabling

(14) $$v_{4}(t)\leq M_{4}\;\mathrm{as}\;t>T_{4}$$

In contrast, considering the primary equation of model (3), along with the integration of (7) and (8), we derive that

$$\begin{array}{ll} \frac{dv_{1}(t)}{dt} & =v_{1}(t)[r_{1}(t)-a_{11}(t)v_{1}(t-\tau_{11})-a_{12}(t)v_{2}(t-\tau_{12})-\frac{a_{13}(t)v_{3}(t)}{b_{13}(t)v_{3}(t)+v_{1}(t)}-\frac{a_{14}(t)v_{4}(t)}{b_{14}(t)v_{4}(t)+v_{1}(t)}-k_{1}(t)u_{1}(t)]\\ & \geq v_{1}(t)[r_{1}^{l}-a_{11}^{m}v_{1}(t-\tau_{11})-a_{12}^{m}M_{2}-\frac{a_{13}^{m}}{b_{13}^{l}}-\frac{a_{14}^{m}}{b_{14}^{l}}-k_{1}^{m}N_{1}]\\ & =v_{1}(t)[\frac{(r_{1}^{l}-a_{12}^{m}M_{2}-k_{1}^{m}N_{1})b_{13}^{l}b_{14}^{l}-a_{13}^{m}b_{14}^{l}-a_{14}^{m}b_{13}^{l}}{b_{13}^{l}b_{14}^{l}}-a_{11}^{m}v_{1}(t-\tau_{11})]. \end{array}$$

By $H_{5}$ and the Lemma 2.3 in [40], one has

(15) $$\begin{array}{ll} \underset{t\to +\infty }{\lim\inf\;}v_{1}(t) & \geq \frac{(r_{1}^{l}-a_{12}^{m}M_{2}-k_{1}^{m}N_{1})b_{13}^{l}b_{14}^{l}-a_{13}^{m}b_{14}^{l}-a_{14}^{m}b_{13}^{l}}{b_{13}^{l}b_{14}^{l}a_{11}^{m}}\\ & \;\cdot \exp[(\frac{(r_{1}^{l}-a_{12}^{m}M_{2}-k_{1}^{m}N_{1})b_{13}^{l}b_{14}^{l}-a_{13}^{m}b_{14}^{l}-a_{14}^{m}b_{13}^{l}}{b_{13}^{l}b_{14}^{l}}-a_{11}^{m}M_{1})\tau_{11}]=m_{1}. \end{array}$$

Similarly, from the second equation of model (3), and combining (6), (9), $H_{6}$, and the Lemma 2.3 in [40], it holds that

(16) $$\begin{array}{ll} \underset{t\to +\infty }{\lim\inf\;}v_{2}(t) & \geq \frac{(r_{2}^{l}-a_{21}^{m}M_{1}-k_{2}^{m}N_{2})b_{23}^{l}b_{24}^{l}-a_{23}^{m}b_{24}^{l}-a_{24}^{m}b_{23}^{l}}{b_{23}^{l}b_{24}^{l}a_{22}^{m}}\\ & \;\cdot \exp[(\frac{(r_{2}^{l}-a_{21}^{m}M_{1}-k_{2}^{m}N_{2})b_{23}^{l}b_{24}^{l}-a_{23}^{m}b_{24}^{l}-a_{24}^{m}b_{23}^{l}}{b_{23}^{l}b_{24}^{l}}-a_{22}^{m}M_{2})\tau_{22}]=m_{2}. \end{array}$$

According to the fifth equation of model (3), and combining (15), one has

$$\frac{du_{1}(t)}{dt}=e_{1}(t)-f_{1}(t)u_{1}(t)+q_{1}(t)v_{1}(t)\geq e_{1}^{l}-f_{1}^{m}u_{1}(t)+q_{1}^{l}m_{1}.$$

From the Lemma 2.1 in [33], it follows that

(17) $$\underset{t\to +\infty }{\lim\sup\;}u_{1}(t)\geq \frac{e_{1}^{l}+q_{1}^{l}m_{1}}{f_{1}^{m}}=n_{1}.$$

Similarly, from the sixth equation of model (3), and combining (16), it holds that

(18) $$\underset{t\to +\infty }{\lim\sup\;}u_{2}(t)\geq \frac{e_{2}^{l}+q_{2}^{l}m_{2}}{f_{2}^{m}}=n_{2}.$$

According to the seventh and eighth equations of system (3), and combining (13) and (14), we have

$$\frac{du_{3}(t)}{dt}=e_{3}(t)-f_{3}(t)u_{3}(t)-q_{3}(t)v_{3}(t)\geq e_{3}^{l}-f_{3}^{m}u_{3}(t)-q_{3}^{m}M_{3},$$

and

$$\frac{du_{4}(t)}{dt}=e_{4}(t)-f_{4}(t)u_{4}(t)-q_{4}(t)v_{4}(t)\geq e_{4}^{l}-f_{4}^{m}u_{4}(t)-q_{4}^{m}M_{4}.$$

From the Lemma 2.1 in [33], it follows that

(19) $$\underset{t\to +\infty }{\lim\sup\;}u_{3}(t)\leq \frac{e_{3}^{l}-q_{3}^{m}M_{3}}{f_{3}^{m}}=n_{3},$$

and

(20) $$\underset{t\to +\infty }{\lim\sup\;}u_{4}(t)\geq \frac{e_{4}^{l}-q_{4}^{m}M_{4}}{f_{4}^{m}}=n_{4}.$$

Based on the third of model (3), and combining (5), (6), (13)–(15), (19), and $H_{7}$, we have

$$\begin{array}{ll} \frac{dv_{3}(t)}{dt} & =v_{3}(t)[-r_{3}(t)+\frac{a_{31}(t)v_{1}(t-\tau_{31})}{b_{13}(t)v_{3}(t)+v_{1}(t-\tau_{31})}+\frac{a_{32}(t)v_{2}(t-\tau_{32})}{b_{23}(t)v_{3}(t)+v_{2}(t-\tau_{32})}-a_{34}(t)v_{4}(t-\tau_{34})+k_{3}(t)u_{3}(t)]\\ & \geq v_{3}(t)[-r_{3}^{m}+\frac{a_{31}^{l}m_{1}}{b_{13}^{m}v_{3}(t)+M_{1}}-a_{34}^{m}M_{4}+k_{3}^{l}n_{3})]\\ & =v_{3}(t)\frac{(-r_{3}^{m}-a_{34}^{m}M_{4}+k_{3}^{l}n_{3})(b_{13}^{m}v_{3}(t)+M_{1})+a_{31}^{l}m_{1}}{b_{13}^{m}v_{3}(t)+M_{1}}\\ & =v_{3}(t)\frac{b_{13}^{m}(r_{3}^{m}+a_{34}^{m}M_{4}-k_{3}^{l}n_{3})}{b_{13}^{m}v_{3}(t)+M_{1}}[-v_{3}(t)-\frac{M_{1}}{b_{13}^{m}}+\frac{a_{31}^{l}m_{1}}{b_{13}^{m}(r_{3}^{m}+a_{34}^{m}M_{4}-k_{3}^{l}n_{3})}]\\ & =v_{3}(t)\frac{b_{13}^{m}(r_{3}^{m}+a_{34}^{m}M_{4}-k_{3}^{l}n_{3})}{b_{13}^{m}v_{3}(t)+M_{1}}[-v_{3}(t)+\frac{a_{31}^{l}m_{1}-M_{1}(r_{3}^{m}+a_{34}^{m}M_{4}-k_{3}^{l}n_{3})}{b_{13}^{m}(r_{3}^{m}+a_{34}^{m}M_{4}-k_{3}^{l}n_{3})}]\\ & >v_{3}(t)\frac{b_{13}^{m}(r_{3}^{m}+a_{34}^{m}M_{4}-k_{3}^{l}n_{3})}{b_{13}^{l}M_{3}+M_{1}}[-v_{3}(t)+m_{3}^{*}]>v_{3}(t)\frac{b_{13}^{m}(r_{3}^{m}+a_{34}^{m}M_{4}-k_{3}^{l}n_{3})}{b_{13}^{l}M_{3}+M_{1}}[-v_{3}(t)+m_{3}]. \end{array}$$

By the comparison theorem of the ordinary differential equation and $H_{9}$, it holds that

5. When $m_{3}<v_{3}(t_{0})$, if $t\geq t_{0}$, then $m_{3}\leq v_{3}(t)$.
6. When $v_{3}(t_{0})\geq m_{3}$, for a enough large t, we have $m_{3}\leq v_{3}(t)$. Otherwise, if $v_{3}(t)<m_{3}$, then there exists β>0 such that $v_{3}(t)\leq m_{3}^{*}-\beta$. Furthermore, we can obtain
7.
${\left.\dot{v}3(t)\right|}_{v_{3}(t)<m_{3}}\geq \frac{b_{13}^{m}(r_{3}^{m}+a_{34}^{m}M_{4}-k_{3}^{l}n_{3})}{b_{13}^{l}M_{3}+M_{1}}v_{3}(t)[-v_{3}(t)+m_{3}^{*}]>\frac{b_{13}^{m}(r_{3}^{m}+a_{34}^{m}M_{4}-k_{3}^{l}n_{3})}{b_{13}^{l}M_{3}+M_{1}}\beta v_{1}(t),$

Thus, from $H_{7}$ it holds that

(21) $$v_{3}(t)\geq v_{3}(t_{0})\exp(\frac{b_{13}^{m}(r_{3}^{m}+a_{34}^{m}M_{4}-k_{3}^{l}n_{3})}{b_{13}^{l}M_{3}+M_{1}}\beta t)\to +\infty \;\mathrm{as}\;t\to +\infty$$

It is obvious that the inequality (21) contradicts $v_{3}(t)<m_{3}$. Therefore, it follows that there exists a large enough $T_{3}^{\prime }\geq t_{0}\geq 0$ such that

(22) $$v_{3}(t)\geq m_{3}\;\mathrm{as}\;t>T_{3}^{\prime }$$

Analogously, from the fourth equation of the model (3), and combining (5), (6), (13), (14), (20), $H_{8}$, and $H_{10}$, it holds that we can select an adequately large positive constant $T_{4}^{\prime }$, enabling

(23) $$v_{4}(t)\geq m_{4}\;\mathrm{as}\;t>T_{4}^{\prime }$$

From (6)–(11), (13)–(20), (22), and (23), we see that the model (3) is permanent, see (Definition 2.1, [33]) for the definition of permanent property. □

**Theorem 3.**  *Assume that* $\left(H_{1})-(H_{10}\right)$ *hold. Then, there exists a strictly positive, spatially homogeneous,* ω- *periodic solution to system (1) and (2).*

**Proof.**  Let $V=C([-\tau ,+\infty ),R_{+}^{8})$ be a Banach space consisting of continuous, bounded, ω-periodic, and positive functions defined on [−τ,+∞), equipped with the infinite norm. Based on the existence and uniqueness theorem of solutions of the functional differential equations, see (Theorem 2.3, page 42 of [41]), we define a Poincaré mapping φ: V→V in the following form:

$$\varphi (U_{0})=U(t,\;\omega ,\;U_{0})$$

where $U(t,\omega ,U_{0})=\left(v_{1}(t),v_{2}(t),v_{3}(t),v_{4}(t),u_{1}(t),u_{2}(t),u_{3}(t),u_{4}(t)\right)$ is a positive solution of (3) and (4).

It is easy to see that φ is continuously mapping by using the continuity of the solution of the functional differential equation (3) with regard to the initial conditions (4), see (Theorem 4.1, page 46 of [41]). For any bounded set K in V, the uniform boundedness of φ(K) can be proved based on the persistence of solutions to models (3) and (4). Furthermore, according to Theorem 2, the derivative of the mapping φ is also bounded, which can then be used to prove that the φ(K) is equicontinuous. The Arzela-Ascoli theorem implies that φ is completely continuous.

We define

$$S=\left\{(v_{1}(t),v_{2}(t),v_{3}(t),v_{4}(t),u_{1}(t),u_{2}(t),u_{3}(t),u_{4}(t))\in V\left|m_{i}\leq v_{i}(t)\leq M_{i},n_{j}\leq u_{j}(t)\leq N_{j},i,j=1,2,3,4\right.\right\}$$

then it is very clear that S is a closed bounded convex subset of the Banach space V. Based on Theorem 2, φ is a completely continuous mapping from S to S. Thus, by Schauder fixed-point theorem, see (Lemma 2.4, page 40 of [41]), the mapping φ has a fixed point $(v_{1}^{*}(t),v_{2}^{*}(t),v_{3}^{*}(t),v_{4}^{*}(t),u_{1}^{*}(t),u_{2}^{*}(t),u_{3}^{*}(t),u_{4}^{*}(t)),$ which is a strictly positive, spatially homogeneous, ω-periodic solution to system (1) and (2), see (Definition 2.2, [42]). □

## 3. Stability of Spatial Homogeneity Periodic Solution

In this part, the focus is on outlining the criteria that uphold the spatial homogeneity and global asymptotic stability of the ω-periodic solution in models (1) and (2). These criteria are formulated leveraging the Lyapunov stability theory, upper and lower solution techniques for partial differential equations with delay, and the squeezing theorem for limits.

**Theorem 4.**  *Assume that assumptions *$\left(H_{1})-(H_{10}\right)$*, along with the following additional assumptions, are satisfied.*

$$\begin{array}{l} \left(H_{11})\;A_{1}=a_{11}^{l}-a_{21}^{m}-q_{1}^{m}-(a_{11}^{m}\tau_{11}+a_{21}^{m}\tau_{21})[r_{1}^{m}+a_{11}^{m}M_{1}+a_{12}^{m}M_{2}+\frac{a_{13}^{m}M_{3}}{b_{13}^{l}m_{3}+m_{1}}+\frac{a_{14}^{m}M_{4}}{b_{14}^{l}m_{4}+m_{1}}+k_{1}^{m}N_{1}\right]\\ \;\;\;\;-(M_{1}a_{11}^{m}\tau_{11}+M_{1}a_{21}^{m}\tau_{21}+1+b_{13}^{m})\frac{a_{13}^{m}M_{3}}{{\left(b_{13}^{l}m_{3}+m_{1}\right)}^{2}}-(M_{1}a_{11}^{m}\tau_{11}+M_{1}a_{21}^{m}\tau_{21}+1+b_{14}^{m})\frac{a_{14}^{m}M_{4}}{{\left(b_{14}^{l}m_{4}+m_{1}\right)}^{2}}\\ \;\;\;\;-M_{1}{\left(a_{11}^{m}\right)}^{2}\tau_{11}-M_{2}a_{21}^{m}a_{12}^{m}\tau_{12}-M_{2}a_{21}^{m}a_{22}^{m}\tau_{22}-M_{1}a_{21}^{m}a_{11}^{m}\tau_{21}\\ \;\;\;\;-\frac{a_{34}^{m}\tau_{34}a_{41}^{m}b_{14}^{m}{\left(M_{4}\right)}^{2}}{{\left(b_{14}^{l}m_{4}+m_{1}\right)}^{2}}-\frac{a_{43}^{m}\tau_{43}a_{31}^{m}b_{13}^{m}{\left(M_{3}\right)}^{2}}{{\left(b_{13}^{l}m_{3}+m_{1}\right)}^{2}}>0, \end{array}$$

$$\begin{array}{l} \left(H_{12})\;A_{2}=a_{22}^{l}-a_{12}^{m}-q_{2}^{m}-(a_{12}^{m}\tau_{12}+a_{22}^{m}\tau_{22})[r_{2}^{m}+a_{22}^{m}M_{2}+a_{21}^{m}M_{1}+\frac{a_{23}^{m}M_{3}}{b_{23}^{l}m_{3}+m_{2}}+\frac{a_{24}^{m}M_{4}}{b_{24}^{l}m_{4}+m_{2}}+k_{2}^{m}N_{2}\right]\\ \;\;-(M_{2}a_{12}^{m}\tau_{12}+M_{2}a_{22}^{m}\tau_{22}+1+b_{23}^{m})\frac{a_{23}^{m}M_{3}}{{\left(b_{23}^{l}m_{3}+m_{2}\right)}^{2}}-(M_{2}a_{12}^{m}\tau_{12}+M_{2}a_{22}^{m}\tau_{22}+1+b_{24}^{m})\frac{a_{24}^{m}M_{4}}{{\left(b_{24}^{l}m_{4}+m_{2}\right)}^{2}}\\ \;\;-M_{1}a_{12}^{m}a_{11}^{m}\tau_{11}-M_{2}a_{12}^{m}a_{22}^{m}\tau_{12}-M_{2}{\left(a_{22}^{m}\right)}^{2}\tau_{22}-M_{1}a_{12}^{m}a_{21}^{m}\tau_{21}\\ \;\;-\frac{a_{34}^{m}\tau_{34}a_{42}^{m}b_{24}^{m}{\left(M_{4}\right)}^{2}}{{\left(b_{24}^{l}m_{4}+m_{2}\right)}^{2}}-\frac{a_{43}^{m}\tau_{43}a_{32}^{m}b_{23}^{m}{\left(M_{3}\right)}^{2}}{{\left(b_{23}^{l}m_{3}+m_{2}\right)}^{2}}>0, \end{array}$$

$$\begin{array}{l} (H_{13})\;A_{3}=\frac{a_{31}^{l}b_{13}^{l}m_{2}}{{\left(b_{13}^{m}M_{3}+M_{1}\right)}^{2}}+\frac{a_{32}^{l}b_{23}^{l}m_{2}}{{\left(b_{23}^{m}M_{3}+M_{2}\right)}^{2}}-a_{34}^{m}\tau_{34}a_{43}^{m}M_{4}-a_{43}^{m}-q_{3}^{m}\\ \;\;-\frac{a_{13}^{m}M_{1}+a_{13}^{m}{\left(M_{1}\right)}^{2}(a_{11}^{m}\tau_{11}+a_{21}^{m}\tau_{21})+a_{43}^{m}\tau_{43}a_{31}^{m}b_{13}^{m}M_{1}M_{3}}{{\left(b_{13}^{l}m_{3}+m_{1}\right)}^{2}}\\ \;\;-\frac{a_{23}^{m}{\left(M_{2}\right)}^{2}a_{12}^{m}\tau_{12}+a_{43}^{m}\tau_{43}a_{32}^{m}b_{23}^{m}M_{2}M_{3}+a_{23}^{m}M_{2}+a_{23}^{m}{\left(M_{2}\right)}^{2}a_{22}^{m}\tau_{22}}{{\left(b_{23}^{l}m_{3}+m_{2}\right)}^{2}}\\ \;\;-a_{43}^{m}\tau_{43}[r_{3}^{m}+\frac{a_{31}^{m}M_{1}}{b_{13}^{l}m_{3}+m_{1}}+\frac{a_{32}^{m}M_{2}}{b_{23}^{l}m_{3}+m_{2}}+a_{34}^{m}M_{4}+k_{3}^{m}N_{3}^{m}]>0, \end{array}$$

$$\begin{array}{l} (H_{14})\;A_{4}=\frac{a_{41}^{l}b_{14}^{l}m_{2}}{{\left(b_{14}^{m}M_{4}+M_{1}\right)}^{2}}+\frac{a_{42}^{l}b_{24}^{l}m_{2}}{{\left(b_{24}^{m}M_{4}+M_{2}\right)}^{2}}-a_{34}^{m}-a_{43}^{m}\tau_{43}a_{34}^{m}M_{3}-q_{4}^{m}\\ \;\;-\frac{a_{14}^{m}M_{1}+a_{14}^{m}{\left(M_{1}\right)}^{2}a_{11}^{m}\tau_{11}+a_{14}^{m}{\left(M_{1}\right)}^{2}a_{21}^{m}\tau_{21}+a_{34}^{m}\tau_{34}a_{41}^{m}b_{14}^{m}M_{1}M_{4}}{{\left(b_{14}^{l}m_{4}+m_{1}\right)}^{2}}\\ \;\;-\frac{a_{24}^{m}{\left(M_{2}\right)}^{2}(a_{12}^{m}\tau_{12}+a_{22}^{m}\tau_{22})+a_{34}^{m}\tau_{34}a_{42}^{m}b_{24}^{m}M_{2}M_{4}+a_{24}^{m}M_{2}}{{\left(b_{24}^{l}m_{4}+m_{2}\right)}^{2}}\\ \;\;-a_{34}^{m}\tau_{34}[r_{4}^{m}+\frac{a_{41}^{m}M_{1}}{b_{14}^{l}m_{4}+m_{1}}+\frac{a_{42}^{m}M_{2}}{b_{24}^{l}m_{4}+m_{2}}+a_{43}^{m}M_{3}+k_{4}^{m}N_{4}^{m}]>0, \end{array}$$

$$(H_{15})\;B_{1}=f_{1}^{l}-k_{1}^{m}(1+M_{1}a_{11}^{m}\tau_{11})-M_{1}k_{1}^{m}a_{21}^{m}\tau_{21}>0,$$

$$(H_{16})\;B_{2}=f_{2}^{l}-M_{2}k_{2}^{m}a_{12}^{m}\tau_{12}-k_{2}^{m}(1+M_{2}a_{22}^{m}\tau_{22})>0,$$

$$(H_{17})\;B_{3}=f_{3}^{l}-k_{3}^{m}-a_{43}^{m}\tau_{43}k_{3}^{m}M_{3}>0,$$

$$(H_{18})\;B_{4}=f_{4}^{l}-k_{4}^{m}-a_{34}^{m}\tau_{34}k_{4}^{m}M_{4}>0.$$

then there is a spatial homogeneity strictly positive ω-periodic solution $\left(v_{1}^{*}(t),v_{2}^{*}(t),v_{3}^{*}(t),v_{4}^{*}(t),u_{1}^{*}(t),u_{2}^{*}(t),u_{3}^{*}(t),u_{4}^{*}(t)\right)$. And the ω-periodic solution is globally asymptotically stable, i.e., the solution $\left(v_{1}(x,t),v_{2}(x,t),v_{3}(x,t),v_{4}(x,t),u_{1}(x,t),u_{2}(x,t),u_{3}(x,t),u_{4}(x,t)\right)$ of model (3) with any positive initial value (4) fulfills

(24) $$\underset{t\to \infty }{\lim\;}(v_{i}(x,t)-v_{i}^{*}(t))=0,\;\underset{t\to \infty }{\lim\;}(u_{i}(x,t)-u_{i}^{*}(t))=0,\mathrm{uniformly}\;\mathrm{for},\;x\in \bar{\Omega },\;i=1,2,3,4.$$

**Proof.**  Based on Theorem 2, the system comprising equations (1) and (2) possesses a spatially homogeneous, strictly positive, and ω-periodic solution. In this context, we establish the stability of this solution. According to Theorem 1 and the fact that the parameters in equations (1) and (2) are positive continuous functions, it can be deduced that the right-hand side functions of equations (1) and (2) satisfy mixed quasimonotonicity, thereby fulfilling the conditions of Theorem 2.1 in reference [43]. Let $l_{i}=\underset{x\in \bar{\Omega },t\in [0,\tau ]}{\min}\eta_{i0}(x,t),\;l_{i}^{*}=\underset{x\in \bar{\Omega },t\in [0,\tau ]}{\min}\mu_{i0}(x,t),\;r_{i}=\underset{x\in \bar{\Omega },t\in [0,\tau ]}{\max}\eta_{i0}(x,t),\;r_{i}^{*}=\underset{x\in \bar{\Omega },t\in [0,\tau ]}{\max}\mu_{i0}(x,t),\;$ then $0<l_{i}\leq \eta_{i0}(x,t)\leq r_{i}.\;0<l_{i}^{*}\leq \mu_{i0}(x,t)\leq r_{i}^{*}.$ Let $\left({\tilde{v}}_{1}(t),{\tilde{v}}_{2}(t),{\tilde{v}}_{3}(t),{\tilde{v}}_{4}(t),{\tilde{u}}_{1}(t),{\tilde{u}}_{2}(t),{\tilde{u}}_{3}(t),{\tilde{u}}_{4}(t)\right)$ and $({\hat{v}}_{1}(t),{\hat{v}}_{2}(t),{\hat{v}}_{3}(t),{\hat{v}}_{4}(t),\;{\hat{u}}_{1}(t),{\hat{u}}_{2}(t),{\hat{u}}_{3}(t),{\hat{u}}_{4}(t))$ are the solutions for (3) subject to initial values $\left(\eta_{10}(t),\eta_{20}(t),\eta_{30}(t),\eta_{40}(t),\mu_{10}(t),\mu_{20}(t),\mu_{30}(t),\mu_{40}(t))=(r_{1},r_{2},r_{3},r_{4},r_{1}^{*},r_{2}^{*},r_{3}^{*},r_{4}^{*}\right)$ and, respectively, then there exists a pair of ordered upper and lower solutions $\left({\tilde{v}}_{1}(t),{\tilde{v}}_{2}(t),{\tilde{v}}_{3}(t),{\tilde{v}}_{4}(t),{\tilde{u}}_{1}(t),{\tilde{u}}_{2}(t),{\tilde{u}}_{3}(t),{\tilde{u}}_{4}(t)\right)$ and $\left({\hat{v}}_{1}(t),{\hat{v}}_{2}(t),{\hat{v}}_{3}(t),{\hat{v}}_{4}(t),{\hat{u}}_{1}(t),{\hat{u}}_{2}(t),{\hat{u}}_{3}(t),{\hat{u}}_{4}(t)\right)$ for (1) and (2). Therefore, from Theorem 2.1 in [43], (1) and (2) have a unique solution.

$$(v_{1}(x,t),v_{2}(x,t),v_{3}(x,t),v_{4}(x,t),u_{1}(x,t),u_{2}(x,t),u_{3}(x,t),u_{4}(x,t)),\;(x,t)\in \bar{\Omega }\times R^{+},$$

which satisfies

$$\begin{array}{ll} \left({\hat{v}}_{1}(t),{\hat{v}}_{2}(t),{\hat{v}}_{3}(t),{\hat{v}}_{4}(t),{\hat{u}}_{1}(t),{\hat{u}}_{2}(t),{\hat{u}}_{3}(t),{\hat{u}}_{4}(t)\right) & \leq (v_{1}(x,t),v_{2}(x,t),v_{3}(x,t),v_{4}(x,t),u_{1}(x,t),u_{2}(x,t),u_{3}(x,t),u_{4}(x,t))\\ & \leq ({\tilde{v}}_{1}(t),{\tilde{v}}_{2}(t),{\tilde{v}}_{3}(t),{\tilde{v}}_{4}(t),{\tilde{u}}_{1}(t),{\tilde{u}}_{2}(t),{\tilde{u}}_{3}(t),{\tilde{u}}_{4}(t)). \end{array}$$

We prove

(25) $$\underset{t\to \infty }{\lim\;}({\tilde{v}}_{i}(t)-v_{i}^{*}(t))=\underset{t\to \infty }{\lim\;}({\hat{v}}_{i}(t)-v_{i}^{*}(t))=0,\underset{t\to \infty }{\lim\;}({\tilde{u}}_{i}(t)-u_{i}^{*}(t))=\underset{t\to \infty }{\lim\;}({\hat{u}}_{i}(t)-u_{i}^{*}(t))=0.$$

We first prove that the solution $\left(v_{1}(t),v_{2}(t),v_{3}(t),v_{4}(t),u_{1}(t),u_{2}(t),u_{3}(t),u_{4}(t)\right)$ for the functional differential equation (3) with any positive initial $(\eta_{10}(t),\eta_{20}(t),\eta_{30}(t),\eta_{40}(t),\mu_{10}(t),\mu_{20}(t),\mu_{30}(t),\mu_{40}(t)).$ satisfies

(26) $$\underset{t\to \infty }{\lim\;}\left(v_{i}(t)-v_{i}^{*}(t)\right)=0,\;\underset{t\to \infty }{\lim\;}\left(u_{i}(t)-u_{i}^{*}(t)\right)=0.$$

Based on Theorem 2, there exists positive real numbers $M_{i},m_{i},N_{i},n_{i}$ and T,such that

$$m_{i}\leq v_{i}(t)\leq M_{i},n_{i}\leq u_{i}(t)\leq N_{i},\;\mathrm{when}\;t>T.$$

Let

$$V_{11}(t)=\left|\ln v_{1}(t)-\ln v_{1}^{*}(t)\right|,$$

we denote by $D^{+}V_{11}(t)$ the right-side derivative of $V_{11}(t)$, then

(27) $$\begin{array}{ll} D^{+}V_{11}(t) & =\mathrm{sgn}(v_{1}(t)-v_{1}^{*}(t))[-a_{11}(t)(v_{1}(t-\tau_{11})-v_{1}^{*}(t-\tau_{11}))-a_{12}(t)(v_{2}(t-\tau_{12})-v_{2}^{*}(t-\tau_{12}))\\ & \;-a_{13}(t)(\frac{v_{3}(t)}{b_{13}(t)v_{3}(t)+v_{1}(t)}-\frac{v_{3}^{*}(t)}{b_{13}(t)v_{3}^{*}(t)+v_{1}^{*}(t)})-a_{14}(t)(\frac{v_{4}(t)}{b_{14}(t)v_{4}(t)+v_{1}(t)}-\frac{v_{4}^{*}(t)}{b_{14}(t)v_{4}^{*}(t)+v_{1}^{*}(t)})\\ & \;-k_{1}(t)(u_{1}(t)-u_{1}^{*}(t))]\\ & =\mathrm{sgn}(v_{1}(t)-v_{1}^{*}(t))[-a_{11}(t)(v_{1}(t)-v_{1}^{*}(t))-a_{12}(t)(v_{2}(t)-v_{2}^{*}(t))\\ & \;-a_{13}(t)\frac{v_{1}^{*}(t)(v_{3}(t)-v_{3}^{*}(t))-v_{3}^{*}(t)(v_{1}(t)-v_{1}^{*}(t))}{\left(b_{13}(t)v_{3}(t)+v_{1}(t))(b_{13}(t)v_{3}^{*}(t)+v_{1}^{*}(t)\right)}-a_{14}(t)\frac{v_{1}^{*}(t)(v_{4}(t)-v_{4}^{*}(t))-v_{4}^{*}(t)(v_{1}(t)-v_{1}^{*}(t))}{\left(b_{14}(t)v_{4}(t)+v_{1}(t))(b_{14}(t)v_{4}^{*}(t)+v_{1}^{*}(t)\right)}\\ & \;-k_{1}(t)(u_{1}(t)-u_{1}^{*}(t))+a_{11}(t)\int_{t-\tau_{11}}^{t}({\dot{v}}_{1}(\theta )-{\dot{v}}_{1}^{*}(\theta ))d\theta +a_{12}(t)\int_{t-\tau_{12}}^{t}({\dot{v}}_{2}(\theta )-{\dot{v}}_{2}^{*}(\theta ))d\theta ]\\ & =\mathrm{sgn}(v_{1}(t)-v_{1}^{*}(t))\{-a_{11}(t)(v_{1}(t)-v_{1}^{*}(t))-a_{12}(t)(v_{2}(t)-v_{2}^{*}(t))\\ & \;-a_{13}(t)\frac{v_{1}^{*}(t)(v_{3}(t)-v_{3}^{*}(t))-v_{3}^{*}(t)(v_{1}(t)-v_{1}^{*}(t))}{\left(b_{13}(t)v_{3}(t)+v_{1}(t))(b_{13}(t)v_{3}^{*}(t)+v_{1}^{*}(t)\right)}-a_{14}(t)\frac{v_{1}^{*}(t)(v_{4}(t)-v_{4}^{*}(t))-v_{4}^{*}(t)(v_{1}(t)-v_{1}^{*}(t))}{\left(b_{14}(t)v_{4}(t)+v_{1}(t))(b_{14}(t)v_{4}^{*}(t)+v_{1}^{*}(t)\right)}\\ & \;-k_{1}(t)(u_{1}(t)-u_{1}^{*}(t))+a_{11}(t)\int_{t-\tau_{11}}^{t}\{v_{1}(\theta )[r_{1}(\theta )-a_{11}(\theta )v_{1}(\theta -\tau_{11})-a_{12}(\theta )v_{2}(\theta -\tau_{12})-\frac{a_{13}(\theta )v_{3}(\theta )}{b_{13}(\theta )v_{3}(\theta )+v_{1}(\theta )}\\ & \;-\frac{a_{14}(\theta )v_{4}(\theta )}{b_{14}(\theta )v_{4}(\theta )+v_{1}(\theta )}-k_{1}(\theta )u_{1}(\theta )]-v_{1}^{*}(\theta )[r_{1}(\theta )-a_{11}(\theta )v_{1}^{*}(\theta -\tau_{11})-a_{12}(\theta )v_{2}^{*}(\theta -\tau_{12})\\ & \;-\frac{a_{13}(\theta )v_{3}^{*}(\theta )}{b_{13}(\theta )v_{3}^{*}(\theta )+v_{1}^{*}(\theta )}-\frac{a_{14}(\theta )v_{4}^{*}(\theta )}{b_{14}(\theta )v_{4}^{*}(\theta )+v_{1}^{*}(\theta )}-k_{1}(\theta )u_{1}^{*}(\theta )]\}d\theta \\ & \;+a_{12}(t)\int_{t-\tau_{12}}^{t}\{v_{2}(\theta )[r_{2}(\theta )-a_{22}(\theta )v_{2}(\theta -\tau_{22})-a_{21}(\theta )v_{1}(\theta -\tau_{21})-\frac{a_{23}(\theta )v_{3}(\theta )}{b_{23}(\theta )v_{3}(\theta )+v_{2}(\theta )}\\ & \;-\frac{a_{24}(\theta )v_{4}(\theta )}{b_{24}(\theta )v_{4}(\theta )+v_{2}(\theta )}-k_{2}(\theta )u_{2}(\theta )]-v_{2}^{*}(\theta )[r_{2}(\theta )-a_{22}(\theta )v_{2}^{*}(\theta -\tau_{22})\\ & \;-a_{21}(\theta )v_{1}^{*}(\theta -\tau_{21})-\frac{a_{23}(\theta )v_{3}^{*}(\theta )}{b_{23}(\theta )v_{3}^{*}(\theta )+v_{2}^{*}(\theta )}-\frac{a_{24}(\theta )v_{4}^{*}(\theta )}{b_{24}(\theta )v_{4}^{*}(\theta )+v_{2}^{*}(\theta )}-k_{2}(\theta )u_{2}^{*}(\theta )]\}d\theta \}\\ & =\mathrm{sgn}(v_{1}(t)-v_{1}^{*}(t))\{-a_{11}(t)(v_{1}(t)-v_{1}^{*}(t))-a_{12}(t)(v_{2}(t)-v_{2}^{*}(t))\\ & \;-a_{13}(t)\frac{v_{1}^{*}(t)(v_{3}(t)-v_{3}^{*}(t))-v_{3}^{*}(t)(v_{1}(t)-v_{1}^{*}(t))}{\left(b_{13}(t)v_{3}(t)+v_{1}(t))(b_{13}(t)v_{3}^{*}(t)+v_{1}^{*}(t)\right)}-a_{14}(t)\frac{v_{1}^{*}(t)(v_{4}(t)-v_{4}^{*}(t))-v_{4}^{*}(t)(v_{1}(t)-v_{1}^{*}(t))}{\left(b_{14}(t)v_{4}(t)+v_{1}(t))(b_{14}(t)v_{4}^{*}(t)+v_{1}^{*}(t)\right)}\\ & \;-k_{1}(t)(u_{1}(t)-u_{1}^{*}(t))+a_{11}(t)\int_{t-\tau_{11}}^{t}\left\{((v_{1}(\theta )-v_{1}^{*}(\theta )\right)[r_{1}(\theta )-a_{11}(\theta )v_{1}(\theta -\tau_{11})-a_{12}(\theta )v_{2}(\theta -\tau_{12})\\ & \;-\frac{a_{13}(\theta )v_{3}(\theta )}{b_{13}(\theta )v_{3}(\theta )+v_{1}(\theta )}-\frac{a_{14}(\theta )v_{4}(\theta )}{b_{14}(\theta )v_{4}(\theta )+v_{1}(\theta )}-k_{1}(\theta )u_{1}(\theta )]-v_{1}^{*}(\theta )[a_{11}(\theta )(v_{1}(\theta -\tau_{11})-v_{1}^{*}(\theta -\tau_{11}))\\ & \;+a_{12}(\theta )(v_{2}(\theta -\tau_{12})-v_{2}^{*}(\theta -\tau_{12}))+a_{13}(\theta )\frac{v_{1}^{*}(\theta )(v_{3}(\theta )-v_{3}^{*}(\theta ))-v_{3}^{*}(\theta )(v_{1}(\theta )-v_{1}^{*}(\theta ))}{\left(b_{13}(\theta )v_{3}(\theta )+v_{1}(\theta ))(b_{13}(\theta )v_{3}^{*}(\theta )+v_{1}^{*}(\theta )\right)}\\ & \;+a_{14}(\theta )\frac{v_{1}^{*}(\theta )(v_{4}(\theta )-v_{4}^{*}(\theta ))-v_{4}^{*}(\theta )(v_{1}(\theta )-v_{1}^{*}(\theta ))}{\left(b_{14}(\theta )v_{4}(\theta )+v_{1}(\theta ))(b_{14}(\theta )v_{4}^{*}(\theta )+v_{1}^{*}(\theta )\right)}+k_{1}(\theta )(u_{1}(\theta )-u_{1}^{*}(\theta ))]\}d\theta \\ & \;+a_{12}(t)\int_{t-\tau_{12}}^{t}\left\{((v_{2}(\theta )-v_{2}^{*}(\theta )\right)[r_{2}(\theta )-a_{22}(\theta )v_{2}(\theta -\tau_{22})-a_{21}(\theta )v_{1}(\theta -\tau_{21})-\frac{a_{23}(\theta )v_{3}(\theta )}{b_{23}(\theta )v_{3}(\theta )+v_{2}(\theta )}\\ & \;-\frac{a_{24}(\theta )v_{4}(\theta )}{b_{24}(\theta )v_{4}(\theta )+v_{2}(\theta )}-k_{2}(\theta )u_{2}(\theta )]-v_{2}^{*}(\theta )[a_{22}(\theta )(v_{2}(\theta -\tau_{22})-v_{2}^{*}(\theta -\tau_{22}))+a_{21}(\theta )(v_{1}(\theta -\tau_{21})\\ & \;-v_{1}^{*}(\theta -\tau_{21}))+a_{23}(\theta )\frac{v_{2}^{*}(\theta )(v_{3}(\theta )-v_{3}^{*}(\theta ))-v_{3}^{*}(\theta )(v_{2}(\theta )-v_{2}^{*}(\theta ))}{\left(b_{23}(\theta )v_{3}(\theta )+v_{2}(\theta ))(b_{23}(\theta )v_{3}^{*}(\theta )+v_{2}^{*}(\theta )\right)}\\ & \;+a_{24}(\theta )\frac{v_{2}^{*}(\theta )(v_{4}(\theta )-v_{4}^{*}(\theta ))-v_{4}^{*}(\theta )(v_{2}(\theta )-v_{2}^{*}(\theta ))}{\left(b_{24}(\theta )v_{4}(\theta )+v_{2}(\theta ))(b_{24}(\theta )v_{4}^{*}(\theta )+v_{2}^{*}(\theta )\right)}+k_{2}(\theta )(u_{2}(\theta )-u_{2}^{*}(\theta ))]\}d\theta \}\\ & \leq (\frac{a_{13}(t)v_{3}^{*}(t)}{\left(b_{13}(t)v_{3}(t)+v_{1}(t))(b_{13}(t)v_{3}^{*}(t)+v_{1}^{*}(t)\right)}+\frac{a_{14}(t)v_{4}^{*}(t)}{\left(b_{14}(t)v_{4}(t)+v_{1}(t))(b_{14}(t)v_{4}^{*}(t)+v_{1}^{*}(t)\right)}-a_{11}(t))\left|v_{1}(t)-v_{1}^{*}(t)\right|\\ & \;+a_{12}(t)\left|v_{2}(t)-v_{2}^{*}(t)\right|+\frac{a_{13}(t)v_{1}^{*}(t)}{\left(b_{13}(t)v_{3}(t)+v_{1}(t))(b_{13}(t)v_{3}^{*}(t)+v_{1}^{*}(t)\right)}\left|v_{3}(t)-v_{3}^{*}(t)\right|\\ & \;+\frac{a_{14}(t)v_{1}^{*}(t)}{\left(b_{14}(t)v_{4}(t)+v_{1}(t))(b_{14}(t)v_{4}^{*}(t)+v_{1}^{*}(t)\right)}\left|v_{4}(t)-v_{4}^{*}(t)\right|+k_{1}(t)\left|u_{1}(t)-u_{1}^{*}(t)\right|\\ & \;+a_{11}(t)\int_{t-\tau_{11}}^{t}\{[r_{1}(\theta )+a_{11}(\theta )v_{1}(\theta -\tau_{11})+a_{12}(\theta )v_{2}(\theta -\tau_{12})+\frac{a_{13}(\theta )v_{3}(\theta )}{b_{13}(\theta )v_{3}(\theta )+v_{1}(\theta )}+\frac{a_{14}(\theta )v_{4}(\theta )}{b_{14}(\theta )v_{4}(\theta )+v_{1}(\theta )}\\ & \;+k_{1}(\theta )u_{1}(\theta )]\left|v_{1}(\theta )-v_{1}^{*}(\theta )\right|+v_{1}^{*}(\theta )[a_{11}(\theta )\left|v_{1}(\theta -\tau_{11})-v_{1}^{*}(\theta -\tau_{11})\right|+a_{12}(\theta )\left|v_{2}(\theta -\tau_{12})-v_{2}^{*}(\theta -\tau_{12})\right|\\ & \;+\frac{a_{13}(\theta )v_{1}^{*}(\theta )}{\left(b_{13}(\theta )v_{3}(\theta )+v_{1}(\theta ))(b_{13}(\theta )v_{3}^{*}(\theta )+v_{1}^{*}(\theta )\right)}\left|v_{3}(\theta )-v_{3}^{*}(\theta )\right|+(\frac{a_{13}(\theta )v_{3}^{*}(\theta )}{\left(b_{13}(\theta )v_{3}(\theta )+v_{1}(\theta ))(b_{13}(\theta )v_{3}^{*}(\theta )+v_{1}^{*}(\theta )\right)}\\ & \;+\frac{a_{14}(\theta )v_{4}^{*}(\theta )}{\left(b_{14}(\theta )v_{4}(\theta )+v_{1}(\theta ))(b_{14}(\theta )v_{4}^{*}(\theta )+v_{1}^{*}(\theta )\right)})\left|v_{1}(\theta )-v_{1}^{*}(\theta )\right|\\ & \;+\frac{a_{14}(\theta )v_{1}^{*}(\theta )}{\left(b_{14}(\theta )v_{4}(\theta )+v_{1}(\theta ))(b_{14}(\theta )v_{4}^{*}(\theta )+v_{1}^{*}(\theta )\right)}\left|v_{4}(\theta )-v_{4}^{*}(\theta )\right|+k_{1}(\theta )\left|u_{1}(\theta )-u_{1}^{*}(\theta )\right|]\}d\theta \\ & \;+a_{12}(t)\int_{t-\tau_{12}}^{t}\{[r_{2}(\theta )+a_{22}(\theta )v_{2}(\theta -\tau_{12})+a_{21}(\theta )v_{1}(\theta -\tau_{21})+\frac{a_{23}(\theta )v_{3}(\theta )}{b_{23}(\theta )v_{3}(\theta )+v_{2}(\theta )}+\frac{a_{24}(\theta )v_{4}(\theta )}{b_{24}(\theta )v_{4}(\theta )+v_{2}(\theta )}\\ & \;+k_{2}(\theta )u_{2}(\theta )]\left|v_{2}(\theta )-v_{2}^{*}(\theta )\right|+v_{2}^{*}(\theta )[a_{22}(\theta )\left|v_{2}(\theta -\tau_{22})-v_{2}^{*}(\theta -\tau_{22})\right|+a_{21}(\theta )\left|v_{1}(\theta -\tau_{21})-v_{1}^{*}(\theta -\tau_{21})\right|\\ & +\frac{a_{23}(\theta )v_{2}^{*}(\theta )}{\left(b_{23}(\theta )v_{3}(\theta )+v_{2}(\theta ))(b_{23}(\theta )v_{3}^{*}(\theta )+v_{2}^{*}(\theta )\right)}\left|v_{3}(\theta )-v_{3}^{*}(\theta )\right|\\ & \;+(\frac{a_{23}(\theta )v_{3}^{*}(\theta )}{\left(b_{23}(\theta )v_{3}(\theta )+v_{2}(\theta ))(b_{23}(\theta )v_{3}^{*}(\theta )+v_{2}^{*}(\theta )\right)}+\frac{a_{24}(\theta )v_{4}^{*}(\theta )}{\left(b_{24}(\theta )v_{4}(\theta )+v_{2}(\theta ))(b_{24}(\theta )v_{4}^{*}(\theta )+v_{2}^{*}(\theta )\right)})\left|v_{2}(\theta )-v_{2}^{*}(\theta )\right|\\ & \;+\frac{a_{24}(\theta )v_{2}^{*}(\theta )}{\left(b_{24}(\theta )v_{4}(\theta )+v_{2}(\theta ))(b_{24}(\theta )v_{4}^{*}(\theta )+v_{2}^{*}(\theta )\right)}\left|v_{4}(\theta )-v_{4}^{*}(\theta )\right|+k_{2}(\theta )\left|u_{2}(\theta )-u_{2}^{*}(\theta )\right|]\}d\theta . \end{array}$$

To eliminate the delay term, we redefine it again.

(28) $$\begin{array}{ll} V_{12}(t) & =\int_{t-\tau_{11}}^{t}\int_{s}^{t}\left\{a_{11}(s+\tau_{11}\right)[r_{1}(\theta )+a_{11}(\theta )v_{1}(\theta -\tau_{11})+a_{12}(\theta )v_{2}(\theta -\tau_{12})+\frac{a_{13}(\theta )v_{3}(\theta )}{b_{13}(\theta )v_{3}(\theta )+v_{1}(\theta )}+\frac{a_{14}(\theta )v_{4}(\theta )}{b_{14}(\theta )v_{4}(\theta )+v_{1}(\theta )}\\ & \;+k_{1}(\theta )u_{1}(\theta )]\left|v_{1}(\theta )-v_{1}^{*}(\theta )\right|+v_{1}^{*}(\theta )[a_{11}(\theta )\left|v_{1}(\theta -\tau_{11})-v_{1}^{*}(\theta -\tau_{11})\right|+a_{12}(\theta )\left|v_{2}(\theta -\tau_{12})-v_{2}^{*}(\theta -\tau_{12})\right|\\ & \;+\frac{a_{13}(\theta )v_{1}^{*}(\theta )}{\left(b_{13}(\theta )v_{3}(\theta )+v_{1}(\theta ))(b_{13}(\theta )v_{3}^{*}(\theta )+v_{1}^{*}(\theta )\right)}]\left|v_{3}(\theta )-v_{3}^{*}(\theta )\right|+v_{1}^{*}(\theta )(\frac{a_{13}(\theta )v_{3}^{*}(\theta )}{\left(b_{13}(\theta )v_{3}(\theta )+v_{1}(\theta ))(b_{13}(\theta )v_{3}^{*}(\theta )+v_{1}^{*}(\theta )\right)}\\ & \;+\frac{a_{14}(\theta )v_{4}^{*}(\theta )}{\left(b_{14}(\theta )v_{4}(\theta )+v_{1}(\theta ))(b_{14}(\theta )v_{4}^{*}(\theta )+v_{1}^{*}(\theta )\right)})\left|v_{1}(\theta )-v_{1}^{*}(\theta )\right|\\ & \;+\frac{a_{14}(\theta ){\left(v_{1}^{*}(\theta )\right)}^{2}}{\left(b_{14}(\theta )v_{4}(\theta )+v_{1}(\theta ))(b_{14}(\theta )v_{4}^{*}(\theta )+v_{1}^{*}(\theta )\right)}\left|v_{4}(\theta )-v_{4}^{*}(\theta )\right|+k_{1}(\theta )v_{1}^{*}(\theta )\left|u_{1}(\theta )-u_{1}^{*}(\theta )\right|\}d\theta ds\\ & \;+\int_{t-\tau_{12}}^{t}\int_{s}^{t}\left\{a_{12}(s+\tau_{12}\right)[r_{2}(\theta )+a_{22}(\theta )v_{2}(\theta -\tau_{12})+a_{21}(\theta )v_{1}(\theta -\tau_{21})+\frac{a_{23}(\theta )v_{3}(\theta )}{b_{23}(\theta )v_{3}(\theta )+v_{2}(\theta )}+\frac{a_{24}(\theta )v_{4}(\theta )}{b_{24}(\theta )v_{4}(\theta )+v_{2}(\theta )}\\ & \;+k_{2}(\theta )u_{2}(\theta )]\left|v_{2}(\theta )-v_{2}^{*}(\theta )\right|+v_{2}^{*}(\theta )[a_{22}(\theta )\left|v_{2}(\theta -\tau_{22})-v_{2}^{*}(\theta -\tau_{22})\right|+a_{21}(\theta )\left|v_{1}(\theta -\tau_{21})-v_{1}^{*}(\theta -\tau_{21})\right|\\ & \;+\frac{a_{23}(\theta )v_{2}^{*}(\theta )}{\left(b_{23}(\theta )v_{3}(\theta )+v_{2}(\theta ))(b_{23}(\theta )v_{3}^{*}(\theta )+v_{2}^{*}(\theta )\right)}\left|v_{3}(\theta )-v_{3}^{*}(\theta )\right|\;+v_{2}^{*}(\theta )(\frac{a_{23}(\theta )v_{3}^{*}(\theta )}{\left(b_{23}(\theta )v_{3}(\theta )+v_{2}(\theta ))(b_{23}(\theta )v_{3}^{*}(\theta )+v_{2}^{*}(\theta )\right)}\\ & \;+\frac{a_{24}(\theta )v_{4}^{*}(\theta )}{\left(b_{24}(\theta )v_{4}(\theta )+v_{2}(\theta ))(b_{24}(\theta )v_{4}^{*}(\theta )+v_{2}^{*}(\theta )\right)})\left|v_{2}(\theta )-v_{2}^{*}(\theta )\right|\\ & \;+\frac{a_{24}(\theta ){\left(v_{2}^{*}(\theta )\right)}^{2}}{\left(b_{24}(\theta )v_{4}(\theta )+v_{2}(\theta ))(b_{24}(\theta )v_{4}^{*}(\theta )+v_{2}^{*}(\theta )\right)}\left|v_{4}(\theta )-v_{4}^{*}(\theta )\right|+k_{2}(\theta )v_{2}^{*}(\theta )\left|u_{2}(\theta )-u_{2}^{*}(\theta )\right|\}d\theta ds. \end{array}$$

By (27) and (28), it holds that

(29) $$\begin{array}{ll} D^{+}\sum_{i=1}^{2}V_{1i}(t) & \leq (\frac{a_{13}(t)v_{3}^{*}(t)}{\left(b_{13}(t)v_{3}(t)+v_{1}(t))(b_{13}(t)v_{3}^{*}(t)+v_{1}^{*}(t)\right)}+\frac{a_{14}(t)v_{4}^{*}(t)}{\left(b_{14}(t)v_{4}(t)+v_{1}(t))(b_{14}(t)v_{4}^{*}(t)+v_{1}^{*}(t)\right)}\\ & \;-a_{11}(t))\left|v_{1}(t)-v_{1}^{*}(t)\right|+a_{12}(t)\left|v_{2}(t)-v_{2}^{*}(t)\right|+\frac{a_{13}(t)v_{1}^{*}(t)}{\left(b_{13}(t)v_{3}(t)+v_{1}(t))(b_{13}(t)v_{3}^{*}(t)+v_{1}^{*}(t)\right)}\left|v_{3}(t)-v_{3}^{*}(t)\right|\\ & \;+\frac{a_{14}(t)v_{1}^{*}(t)}{\left(b_{14}(t)v_{4}(t)+v_{1}(t))(b_{14}(t)v_{4}^{*}(t)+v_{1}^{*}(t)\right)}\left|v_{4}(t)-v_{4}^{*}(t)\right|+k_{1}(t)\left|u_{1}(t)-u_{1}^{*}(t)\right|\\ & \;+\int_{t-\tau_{11}}^{t}a_{11}(s+\tau_{11})ds\{[r_{1}(t)+a_{11}(t)v_{1}(t-\tau_{11})+a_{12}(t)v_{2}(t-\tau_{12})+\frac{a_{13}(t)v_{3}(t)}{b_{13}(t)v_{3}(t)+v_{1}(t)}\\ & \;+\frac{a_{14}(t)v_{4}(t)}{b_{14}(t)v_{4}(t)+v_{1}(t)}+k_{1}(t)u_{1}(t)]\left|v_{1}(t)-v_{1}^{*}(t)\right|+v_{1}^{*}(t)[a_{11}(t)\left|v_{1}(t-\tau_{11})-v_{1}^{*}(t-\tau_{11})\right|\\ & \;+a_{12}(t)\left|v_{2}(t-\tau_{12})-v_{2}^{*}(t-\tau_{12})\right|+\frac{a_{13}(t)v_{1}^{*}(t)}{\left(b_{13}(t)v_{3}(t)+v_{1}(t))(b_{13}(t)v_{3}^{*}(t)+v_{1}^{*}(t)\right)}]\left|v_{3}(t)-v_{3}^{*}(t)\right|\\ & \;+v_{1}^{*}(t)(\frac{a_{13}(t)v_{3}^{*}(t)}{\left(b_{13}(t)v_{3}(t)+v_{1}(t))(b_{13}(t)v_{3}^{*}(t)+v_{1}^{*}(t)\right)}+\frac{a_{14}(t)v_{4}^{*}(t)}{\left(b_{14}(t)v_{4}(t)+v_{1}(t))(b_{14}(t)v_{4}^{*}(t)+v_{1}^{*}(t)\right)})\left|v_{1}(t)-v_{1}^{*}(t)\right|\\ & \;+\frac{a_{14}(t){\left(v_{1}^{*}(t)\right)}^{2}}{\left(b_{14}(t)v_{4}(t)+v_{1}(t))(b_{14}(t)v_{4}^{*}(t)+v_{1}^{*}(t)\right)}\left|v_{4}(t)-v_{4}^{*}(t)\right|+k_{1}(t)v_{1}^{*}(t)\left|u_{1}(t)-u_{1}^{*}(t)\right|\}\\ & \;+\int_{t-\tau_{12}}^{t}a_{12}(s+\tau_{12})ds\{[r_{2}(t)+a_{22}(t)v_{2}(t-\tau_{12})+a_{21}(t)v_{1}(t-\tau_{21})+\frac{a_{23}(t)v_{3}(t)}{b_{23}(t)v_{3}(t)+v_{2}(t)}\\ & \;+\frac{a_{24}(t)v_{4}(t)}{b_{24}(t)v_{4}(t)+v_{2}(t)}+k_{2}(t)u_{2}(t)]\left|v_{2}(t)-v_{2}^{*}(t)\right|+v_{2}^{*}(t)[a_{22}(t)\left|v_{2}(t-\tau_{22})-v_{2}^{*}(t-\tau_{22})\right|\\ & \;+a_{21}(t)\left|v_{1}(t-\tau_{21})-v_{1}^{*}(t-\tau_{21})\right|+\frac{a_{23}(t)v_{2}^{*}(t)}{\left(b_{23}(t)v_{3}(t)+v_{2}(t))(b_{23}(t)v_{3}^{*}(t)+v_{2}^{*}(t)\right)}\left|v_{3}(t)-v_{3}^{*}(t)\right|\\ & \;+v_{2}^{*}(t)(\frac{a_{23}(t)v_{3}^{*}(t)}{\left(b_{23}(t)v_{3}(t)+v_{2}(t))(b_{23}(t)v_{3}^{*}(t)+v_{2}^{*}(t)\right)}+\frac{a_{24}(t)v_{4}^{*}(t)}{\left(b_{24}(t)v_{4}(t)+v_{2}(t))(b_{24}(t)v_{4}^{*}(t)+v_{2}^{*}(t)\right)})\left|v_{2}(t)-v_{2}^{*}(t)\right|\\ & \;+\frac{a_{24}(t){\left(v_{2}^{*}(t)\right)}^{2}}{\left(b_{24}(t)v_{4}(t)+v_{2}(t))(b_{24}(t)v_{4}^{*}(t)+v_{2}^{*}(t)\right)}\left|v_{4}(t)-v_{4}^{*}(t)\right|+k_{2}(t)v_{2}^{*}(t)\left|u_{2}(t)-u_{2}^{*}(t)\right|)\\ & \leq (\frac{a_{13}^{m}M_{3}}{{\left(b_{13}^{l}m_{3}+m_{1}\right)}^{2}}+\frac{a_{14}^{m}M_{4}}{{\left(b_{14}^{l}m_{4}+m_{1}\right)}^{2}}-a_{11}^{l})\left|v_{1}(t)-v_{1}^{*}(t)\right|+a_{12}^{m}\left|v_{2}(t)-v_{2}^{*}(t)\right|\\ & \;+\frac{a_{13}^{m}M_{1}}{{\left(b_{13}^{l}m_{3}+m_{1}\right)}^{2}}\left|v_{3}(t)-v_{3}^{*}(t)\right|+\frac{a_{14}^{m}M_{1}}{{\left(b_{14}^{l}m_{4}+m_{1}\right)}^{2}}\left|v_{4}(t)-v_{4}^{*}(t)\right|+k_{1}^{m}\left|u_{1}(t)-u_{1}^{*}(t)\right|\\ & \;+a_{11}^{m}\tau_{11}[r_{1}^{m}+a_{11}^{m}M_{1}+a_{12}^{m}M_{2}+\frac{a_{13}^{m}M_{3}}{b_{13}^{l}m_{3}+m_{1}}+\frac{a_{14}^{m}M_{4}}{b_{14}^{l}m_{4}+m_{1}}+k_{1}^{m}N_{1}]\left|v_{1}(t)-v_{1}^{*}(t)\right|\\ & \;+M_{1}{\left(a_{11}^{m}\right)}^{2}\tau_{11}\left|v_{1}(t-\tau_{11})-v_{1}^{*}(t-\tau_{11})\right|+M_{1}a_{12}^{m}a_{11}^{m}\tau_{11}\left|v_{2}(t-\tau_{12})-v_{2}^{*}(t-\tau_{12})\right|\\ & \;+\frac{a_{13}^{m}{\left(M_{1}\right)}^{2}a_{11}^{m}\tau_{11}}{{\left(b_{13}^{l}m_{3}+m_{1}\right)}^{2}}\left|v_{3}(t)-v_{3}^{*}(t)\right|+M_{1}a_{11}^{m}\tau_{11}(\frac{a_{13}^{m}M_{3}}{{\left(b_{13}^{l}m_{3}+m_{1}\right)}^{2}}+\frac{a_{14}^{m}M_{4}}{{\left(b_{14}^{l}m_{4}+m_{1}\right)}^{2}})\left|v_{1}(t)-v_{1}^{*}(t)\right|\\ & \;+\frac{a_{14}^{m}{\left(M_{1}\right)}^{2}a_{11}^{m}\tau_{11}}{{\left(b_{14}^{l}m_{4}+m_{1}\right)}^{2}}\left|v_{4}(t)-v_{4}^{*}(t)\right|+M_{1}k_{1}^{m}a_{11}^{m}\tau_{11}\left|u_{1}(t)-u_{1}^{*}(t)\right|\\ & \;+a_{12}^{m}\tau_{12}[r_{2}^{m}+a_{22}^{m}M_{2}+a_{21}^{m}M_{1}+\frac{a_{23}^{m}M_{3}}{b_{23}^{l}m_{3}+m_{2}}+\frac{a_{24}^{m}M_{4}}{b_{24}^{l}m_{4}+m_{2}}+k_{2}^{m}N_{2}]\left|v_{2}(t)-v_{2}^{*}(t)\right|\\ & \;+M_{2}a_{12}^{m}a_{22}^{m}\tau_{12}\left|v_{2}(t-\tau_{22})-v_{2}^{*}(t-\tau_{22})\right|+M_{2}a_{21}^{m}a_{12}^{m}\tau_{12}\left|v_{1}(t-\tau_{21})-v_{1}^{*}(t-\tau_{21})\right|\\ & \;+\frac{a_{23}^{m}{\left(M_{2}\right)}^{2}a_{12}^{m}\tau_{12}}{{\left(b_{23}^{l}m_{3}+m_{2}\right)}^{2}}\left|v_{3}(t)-v_{3}^{*}(t)\right|+M_{2}a_{12}^{m}\tau_{12}(\frac{a_{23}^{m}M_{3}}{{\left(b_{23}^{l}m_{3}+m_{2}\right)}^{2}}+\frac{a_{24}^{m}M_{4}}{{\left(b_{24}^{l}m_{4}+m_{2}\right)}^{2}})\left|v_{2}(t)-v_{2}^{*}(t)\right|\\ & \;+\frac{a_{24}^{m}{\left(M_{2}\right)}^{2}a_{12}^{m}\tau_{12}}{{\left(b_{24}^{l}m_{4}+m_{2}\right)}^{2}}\left|v_{4}(t)-v_{4}^{*}(t)\right|+M_{2}k_{2}^{m}a_{12}^{m}\tau_{12}\left|u_{2}(t)-u_{2}^{*}(t)\right|\\ & \leq \{\frac{a_{13}^{m}M_{3}}{{\left(b_{13}^{l}m_{3}+m_{1}\right)}^{2}}+\frac{a_{14}^{m}M_{4}}{{\left(b_{14}^{l}m_{4}+m_{1}\right)}^{2}}-a_{11}^{l}+a_{11}^{m}\tau_{11}[r_{1}^{m}+a_{11}^{m}M_{1}+a_{12}^{m}M_{2}+\frac{a_{13}^{m}M_{3}}{b_{13}^{l}m_{3}+m_{1}}\\ & \;+\frac{a_{14}^{m}M_{4}}{b_{14}^{l}m_{4}+m_{1}}+k_{1}^{m}N_{1}]+M_{1}a_{11}^{m}\tau_{11}(\frac{a_{13}^{m}M_{3}}{{\left(b_{13}^{l}m_{3}+m_{1}\right)}^{2}}+\frac{a_{14}^{m}M_{4}}{{\left(b_{14}^{l}m_{4}+m_{1}\right)}^{2}})\}\left|v_{1}(t)-v_{1}^{*}(t)\right|\\ & \;+\{a_{12}^{m}+a_{12}^{m}\tau_{12}[r_{2}^{m}+a_{22}^{m}M_{2}+a_{21}^{m}M_{1}+\frac{a_{23}^{m}M_{3}}{b_{23}^{l}m_{3}+m_{2}}+\frac{a_{24}^{m}M_{4}}{b_{24}^{l}m_{4}+m_{2}}+k_{2}^{m}N_{2}]\\ & \;+M_{2}a_{12}^{m}\tau_{12}(\frac{a_{23}^{m}M_{3}}{{\left(b_{23}^{l}m_{3}+m_{2}\right)}^{2}}+\frac{a_{24}^{m}M_{4}}{{\left(b_{24}^{l}m_{4}+m_{2}\right)}^{2}})\}\left|v_{2}(t)-v_{2}^{*}(t)\right|\\ & \;+(\frac{a_{13}^{m}M_{1}+a_{13}^{m}{\left(M_{1}\right)}^{2}a_{11}^{m}\tau_{11}}{{\left(b_{13}^{l}m_{3}+m_{1}\right)}^{2}}+\frac{a_{23}^{m}{\left(M_{2}\right)}^{2}a_{12}^{m}\tau_{12}}{{\left(b_{23}^{l}m_{3}+m_{2}\right)}^{2}})\left|v_{3}(t)-v_{3}^{*}(t)\right|\\ & \;+(\frac{a_{14}^{m}M_{1}+a_{14}^{m}{\left(M_{1}\right)}^{2}a_{11}^{m}\tau_{11}}{{\left(b_{14}^{l}m_{4}+m_{1}\right)}^{2}}+\frac{a_{24}^{m}{\left(M_{2}\right)}^{2}a_{12}^{m}\tau_{12}}{{\left(b_{24}^{l}m_{4}+m_{2}\right)}^{2}})\left|v_{4}(t)-v_{4}^{*}(t)\right|\\ & \;+k_{1}^{m}(1+M_{1}a_{11}^{m}\tau_{11})\left|u_{1}(t)-u_{1}^{*}(t)\right|+M_{2}k_{2}^{m}a_{12}^{m}\tau_{12}\left|u_{2}(t)-u_{2}^{*}(t)\right|\\ & \;+M_{1}{\left(a_{11}^{m}\right)}^{2}\tau_{11}\left|v_{1}(t-\tau_{11})-v_{1}^{*}(t-\tau_{11})\right|+M_{1}a_{12}^{m}a_{11}^{m}\tau_{11}\left|v_{2}(t-\tau_{12})-v_{2}^{*}(t-\tau_{12})\right|\\ & \;+M_{2}a_{12}^{m}a_{22}^{m}\tau_{12}\left|v_{2}(t-\tau_{22})-v_{2}^{*}(t-\tau_{22})\right|+M_{2}a_{21}^{m}a_{12}^{m}\tau_{12}\left|v_{1}(t-\tau_{21})-v_{1}^{*}(t-\tau_{21})\right|. \end{array}$$

Let

(30) $$\begin{array}{ll} V_{13}(t) & =M_{1}{\left(a_{11}^{m}\right)}^{2}\tau_{11}\int_{t-\tau_{11}}^{t}\left|v_{1}(w)-v_{1}^{*}(w)\right|dw+M_{1}a_{12}^{m}a_{11}^{m}\tau_{11}\int_{t-\tau_{12}}^{t}\left|v_{2}(w)-v_{2}^{*}(w)\right|dw\\ & \;+M_{2}a_{12}^{m}a_{22}^{m}\tau_{12}\int_{t-\tau_{11}}^{t}\left|v_{2}(w)-v_{2}^{*}(w)\right|dw+M_{2}a_{21}^{m}a_{12}^{m}\tau_{12}\int_{t-\tau_{21}}^{t}\left|v_{1}(w)-v_{1}^{*}(w)\right|dw, \end{array}$$

and

(31) $$V_{1}(t)=V_{11}(t)+V_{12}(t)+V_{13}(t).$$

By (29) and (30), we have

(32) $$\begin{array}{ll} D^{+}V_{1}(t) & \leq \{\frac{a_{13}^{m}M_{3}}{{\left(b_{13}^{l}m_{3}+m_{1}\right)}^{2}}+\frac{a_{14}^{m}M_{4}}{{\left(b_{14}^{l}m_{4}+m_{1}\right)}^{2}}-a_{11}^{l}+a_{11}^{m}\tau_{11}[r_{1}^{m}+a_{11}^{m}M_{1}+a_{12}^{m}M_{2}+\frac{a_{13}^{m}M_{3}}{b_{13}^{l}m_{3}+m_{1}}\\ & \;+\frac{a_{14}^{m}M_{4}}{b_{14}^{l}m_{4}+m_{1}}+k_{1}^{m}N_{1}]+M_{1}a_{11}^{m}\tau_{11}(\frac{a_{13}^{m}M_{3}}{{\left(b_{13}^{l}m_{3}+m_{1}\right)}^{2}}+\frac{a_{14}^{m}M_{4}}{{\left(b_{14}^{l}m_{4}+m_{1}\right)}^{2}})+M_{1}{\left(a_{11}^{m}\right)}^{2}\tau_{11}\\ & \;+M_{2}a_{21}^{m}a_{12}^{m}\tau_{12}\}\left|v_{1}(t)-v_{1}^{*}(t)\right|+\{a_{12}^{m}+a_{12}^{m}\tau_{12}[r_{2}^{m}+a_{22}^{m}M_{2}+a_{21}^{m}M_{1}+\frac{a_{23}^{m}M_{3}}{b_{23}^{l}m_{3}+m_{2}}\\ & \;+\frac{a_{24}^{m}M_{4}}{b_{24}^{l}m_{4}+m_{2}}+k_{2}^{m}N_{2}]+M_{2}a_{12}^{m}\tau_{12}(\frac{a_{23}^{m}M_{3}}{{\left(b_{23}^{l}m_{3}+m_{2}\right)}^{2}}+\frac{a_{24}^{m}M_{4}}{{\left(b_{24}^{l}m_{4}+m_{2}\right)}^{2}})\\ & \;+M_{1}a_{12}^{m}a_{11}^{m}\tau_{11}+M_{2}a_{12}^{m}a_{22}^{m}\tau_{12}\}\left|v_{2}(t)-v_{2}^{*}(t)\right|+(\frac{a_{13}^{m}M_{1}+a_{13}^{m}{\left(M_{1}\right)}^{2}a_{11}^{m}\tau_{11}}{{\left(b_{13}^{l}m_{3}+m_{1}\right)}^{2}}\\ & \;+\frac{a_{23}^{m}{\left(M_{2}\right)}^{2}a_{12}^{m}\tau_{12}}{{\left(b_{23}^{l}m_{3}+m_{2}\right)}^{2}})\left|v_{3}(t)-v_{3}^{*}(t)\right|+(\frac{a_{14}^{m}M_{1}+a_{14}^{m}{\left(M_{1}\right)}^{2}a_{11}^{m}\tau_{11}}{{\left(b_{14}^{l}m_{4}+m_{1}\right)}^{2}}+\frac{a_{24}^{m}{\left(M_{2}\right)}^{2}a_{12}^{m}\tau_{12}}{{\left(b_{24}^{l}m_{4}+m_{2}\right)}^{2}})\left|v_{4}(t)-v_{4}^{*}(t)\right|\\ & \;+k_{1}^{m}(1+M_{1}a_{11}^{m}\tau_{11})\left|u_{1}(t)-u_{1}^{*}(t)\right|+M_{2}k_{2}^{m}a_{12}^{m}\tau_{12}\left|u_{2}(t)-u_{2}^{*}(t)\right|. \end{array}$$

Analogously, we define

$$V_{21}(t)=\left|\ln v_{2}(t)-\ln v_{2}^{*}(t)\right|.$$

Let $D^{+}V_{21}(t)$ represent the right-hand derivative of $V_{21}(t)$, and it follows that

(33) $$\begin{array}{ll} D^{+}V_{21}(t) & =\mathrm{sgn}(v_{2}(t)-v_{2}^{*}(t))[-a_{22}(t)(v_{2}(t-\tau_{22})-v_{2}^{*}(t-\tau_{22}))-a_{21}(t)(v_{1}(t-\tau_{21})-v_{1}^{*}(t-\tau_{21}))\\ & \;\;-a_{23}(t)(\frac{v_{3}(t)}{b_{23}(t)v_{3}(t)+v_{2}(t)}-\frac{v_{3}^{*}(t)}{b_{23}(t)v_{3}^{*}(t)+v_{2}^{*}(t)})-a_{24}(t)(\frac{v_{4}(t)}{b_{24}(t)v_{4}(t)+v_{2}(t)}-\frac{v_{4}^{*}(t)}{b_{24}(t)v_{4}^{*}(t)+v_{2}^{*}(t)})\\ & \;\;-k_{2}(t)(u_{2}(t)-u_{2}^{*}(t))]\\ & =\mathrm{sgn}(v_{2}(t)-v_{2}^{*}(t))[-a_{22}(t)(v_{2}(t)-v_{2}^{*}(t))-a_{21}(t)(v_{1}(t)-v_{1}^{*}(t))\\ & \;\;-a_{23}(t)\frac{v_{2}^{*}(t)(v_{3}(t)-v_{3}^{*}(t))-v_{3}^{*}(t)(v_{2}(t)-v_{2}^{*}(t))}{\left(b_{23}(t)v_{3}(t)+v_{2}(t))(b_{23}(t)v_{3}^{*}(t)+v_{2}^{*}(t)\right)}-a_{24}(t)\frac{v_{2}^{*}(t)(v_{4}(t)-v_{4}^{*}(t))-v_{4}^{*}(t)(v_{2}(t)-v_{2}^{*}(t))}{\left(b_{24}(t)v_{4}(t)+v_{2}(t))(b_{24}(t)v_{4}^{*}(t)+v_{2}^{*}(t)\right)}\\ & \;\;-k_{2}(t)(u_{2}(t)-u_{2}^{*}(t))+a_{22}(t)\int_{t-\tau_{22}}^{t}({\dot{v}}_{2}(\theta )-{\dot{v}}_{2}^{*}(\theta ))d\theta +a_{21}(t)\int_{t-\tau_{21}}^{t}({\dot{v}}_{1}(\theta )-{\dot{v}}_{1}^{*}(\theta ))d\theta ]\\ & =\mathrm{sgn}(v_{2}(t)-v_{2}^{*}(t))\{-a_{22}(t)(v_{2}(t)-v_{2}^{*}(t))-a_{21}(t)(v_{1}(t)-v_{1}^{*}(t))\\ & \;\;-a_{23}(t)\frac{v_{2}^{*}(t)(v_{3}(t)-v_{3}^{*}(t))-v_{3}^{*}(t)(v_{2}(t)-v_{2}^{*}(t))}{\left(b_{23}(t)v_{3}(t)+v_{2}(t))(b_{23}(t)v_{3}^{*}(t)+v_{2}^{*}(t)\right)}-a_{24}(t)\frac{v_{2}^{*}(t)(v_{4}(t)-v_{4}^{*}(t))-v_{4}^{*}(t)(v_{2}(t)-v_{2}^{*}(t))}{\left(b_{24}(t)v_{4}(t)+v_{2}(t))(b_{24}(t)v_{4}^{*}(t)+v_{2}^{*}(t)\right)}\\ & \;\;-k_{2}(t)(u_{2}(t)-u_{2}^{*}(t))\;\;+a_{22}(t)\int_{t-\tau_{22}}^{t}\{v_{2}(\theta )[r_{2}(\theta )-a_{22}(\theta )v_{2}(\theta -\tau_{22})-a_{21}(\theta )v_{1}(\theta -\tau_{21})-\frac{a_{23}(\theta )v_{3}(\theta )}{b_{23}(\theta )v_{3}(\theta )+v_{2}(\theta )}\\ & \;\;-\frac{a_{24}(\theta )v_{4}(\theta )}{b_{24}(\theta )v_{4}(\theta )+v_{2}(\theta )}-k_{2}(\theta )u_{2}(\theta )]-v_{2}^{*}(t)[r_{2}(\theta )-a_{22}(\theta )v_{2}^{*}(\theta -\tau_{22})-a_{21}(\theta )v_{1}^{*}(\theta -\tau_{21})\\ & \;\;-\frac{a_{23}(\theta )v_{3}^{*}(\theta )}{b_{23}(\theta )v_{3}^{*}(\theta )+v_{2}^{*}(\theta )}-\frac{a_{24}(\theta )v_{4}^{*}(\theta )}{b_{24}(\theta )v_{4}^{*}(\theta )+v_{2}^{*}(\theta )}-k_{2}(\theta )u_{2}^{*}(\theta )]\}d\theta \\ & \;\;+a_{21}(t)\int_{t-\tau_{21}}^{t}\{v_{1}(t)[r_{1}(\theta )-a_{11}(\theta )v_{1}(\theta -\tau_{11})-a_{12}(\theta )v_{2}(\theta -\tau_{12})-\frac{a_{13}(\theta )v_{3}(\theta )}{b_{13}(\theta )v_{3}(\theta )+v_{1}(\theta )}\\ & \;\;-\frac{a_{14}(\theta )v_{4}(\theta )}{b_{14}(\theta )v_{4}(\theta )+v_{1}(\theta )}-k_{1}(\theta )u_{1}(\theta )]-v_{1}^{*}(t)[r_{1}(\theta )-a_{11}(\theta )v_{1}^{*}(\theta -\tau_{11})-a_{12}(\theta )v_{2}^{*}(\theta -\tau_{12})\\ & \;\;-\frac{a_{13}(\theta )v_{3}^{*}(\theta )}{b_{13}(\theta )v_{3}^{*}(\theta )+v_{1}^{*}(\theta )}-\frac{a_{14}(\theta )v_{4}^{*}(\theta )}{b_{14}(\theta )v_{4}^{*}(\theta )+v_{1}^{*}(\theta )}-k_{1}(\theta )u_{1}^{*}(\theta )]\}d\theta \}\\ & =\mathrm{sgn}(v_{2}(t)-v_{2}^{*}(t))\{-a_{22}(t)(v_{2}(t)-v_{2}^{*}(t))-a_{21}(t)(v_{1}(t)-v_{1}^{*}(t))\\ & \;-a_{23}(t)\frac{v_{2}^{*}(t)(v_{3}(t)-v_{3}^{*}(t))-v_{3}^{*}(t)(v_{2}(t)-v_{2}^{*}(t))}{\left(b_{23}(t)v_{3}(t)+v_{2}(t))(b_{23}(t)v_{3}^{*}(t)+v_{2}^{*}(t)\right)}-a_{24}(t)\frac{v_{2}^{*}(t)(v_{4}(t)-v_{4}^{*}(t))-v_{4}^{*}(t)(v_{2}(t)-v_{2}^{*}(t))}{\left(b_{24}(t)v_{4}(t)+v_{2}(t))(b_{24}(t)v_{4}^{*}(t)+v_{2}^{*}(t)\right)}\\ & \;-k_{2}(t)(u_{2}(t)-u_{2}^{*}(t))+a_{22}(t)\int_{t-\tau_{22}}^{t}\left\{(v_{2}(\theta )-v_{2}^{*}(\theta )\right)[r_{2}(\theta )-a_{22}(\theta )v_{2}(\theta -\tau_{22})-a_{21}(\theta )v_{1}(\theta -\tau_{21})\\ & \;-\frac{a_{23}(\theta )v_{3}(\theta )}{b_{23}(\theta )v_{3}(\theta )+v_{2}(\theta )}-\frac{a_{24}(\theta )v_{4}(\theta )}{b_{24}(\theta )v_{4}(\theta )+v_{2}(\theta )}-k_{2}(\theta )u_{2}(\theta )]-v_{2}^{*}(\theta )[a_{22}(\theta )(v_{2}(\theta -\tau_{22})-v_{2}^{*}(\theta -\tau_{22}))\\ & \;+a_{21}(\theta )(v_{1}(\theta -\tau_{21})-v_{1}^{*}(\theta -\tau_{21}))+a_{23}(\theta )\frac{v_{2}^{*}(\theta )(v_{3}(\theta )-v_{3}^{*}(\theta ))-v_{3}^{*}(\theta )(v_{2}(\theta )-v_{2}^{*}(\theta ))}{\left(b_{23}(\theta )v_{3}(\theta )+v_{2}(\theta ))(b_{23}(\theta )v_{3}^{*}(\theta )+v_{2}^{*}(\theta )\right)}\\ & \;+a_{24}(\theta )\frac{v_{2}^{*}(\theta )(v_{4}(\theta )-v_{4}^{*}(\theta ))-v_{4}^{*}(\theta )(v_{2}(\theta )-v_{2}^{*}(\theta ))}{\left(b_{24}(\theta )v_{4}(\theta )+v_{2}(\theta ))(b_{24}(\theta )v_{4}^{*}(\theta )+v_{2}^{*}(\theta )\right)}+k_{2}(\theta )(u_{2}(\theta )-u_{2}^{*}(\theta ))]\}d\theta \\ & \;+a_{21}(t)\int_{t-\tau_{21}}^{t}\left\{(v_{1}(\theta )-v_{1}^{*}(\theta )\right)[r_{1}(\theta )-a_{11}(\theta )v_{1}(\theta -\tau_{11})-a_{12}(\theta )v_{2}(\theta -\tau_{12})-\frac{a_{13}(\theta )v_{3}(\theta )}{b_{13}(\theta )v_{3}(\theta )+v_{1}(\theta )}\\ & \;-\frac{a_{14}(\theta )v_{4}(\theta )}{b_{14}(\theta )v_{4}(\theta )+v_{1}(\theta )}-k_{1}(\theta )u_{1}(\theta )]-v_{1}^{*}(\theta )[a_{11}(\theta )(v_{1}(\theta -\tau_{11})-v_{1}^{*}(\theta -\tau_{11}))+a_{12}(\theta )(v_{2}(\theta -\tau_{12})\\ & \;-v_{2}^{*}(\theta -\tau_{12}))+a_{13}(\theta )\frac{v_{1}^{*}(\theta )(v_{3}(\theta )-v_{3}^{*}(\theta ))-v_{3}^{*}(\theta )(v_{1}(\theta )-v_{1}^{*}(\theta ))}{\left(b_{13}(\theta )v_{3}(\theta )+v_{1}(\theta ))(b_{13}(\theta )v_{3}^{*}(\theta )+v_{1}^{*}(\theta )\right)}\\ & \;+a_{14}(\theta )\frac{v_{1}^{*}(\theta )(v_{4}(\theta )-v_{4}^{*}(\theta ))-v_{4}^{*}(\theta )(v_{1}(\theta )-v_{1}^{*}(\theta ))}{\left(b_{14}(\theta )v_{4}(\theta )+v_{1}(\theta ))(b_{24}(\theta )v_{4}^{*}(\theta )+v_{1}^{*}(\theta )\right)}+k_{1}(\theta )(u_{1}(\theta )-u_{1}^{*}(\theta ))]\}d\theta \}\\ & \leq (\frac{a_{23}(t)v_{3}^{*}(t)}{\left(b_{23}(t)v_{3}(t)+v_{2}(t))(b_{23}(t)v_{3}^{*}(t)+v_{2}^{*}(t)\right)}+\frac{a_{24}(t)v_{4}^{*}(t)}{\left(b_{24}(t)v_{4}(t)+v_{2}(t))(b_{24}(t)v_{4}^{*}(t)+v_{2}^{*}(t)\right)}-a_{22}(t))\left|v_{2}(t)-v_{2}^{*}(t)\right|\\ & \;+a_{21}(t)\left|v_{1}(t)-v_{1}^{*}(t)\right|+\frac{a_{23}(t)v_{2}^{*}(t)}{\left(b_{23}(t)v_{3}(t)+v_{2}(t))(b_{23}(t)v_{3}^{*}(t)+v_{2}^{*}(t)\right)}\left|v_{3}(t)-v_{3}^{*}(t)\right|\\ & \;+\frac{a_{24}(t)v_{2}^{*}(t)}{\left(b_{24}(t)v_{4}(t)+v_{2}(t))(b_{24}(t)v_{4}^{*}(t)+v_{2}^{*}(t)\right)}\left|v_{4}(t)-v_{4}^{*}(t)\right|+k_{2}(t)\left|u_{2}(t)-u_{2}^{*}(t)\right|\\ & \;+a_{22}(t)\int_{t-\tau_{22}}^{t}[r_{2}(\theta )+a_{22}(\theta )v_{2}(\theta -\tau_{22})+a_{21}(\theta )v_{1}(\theta -\tau_{21})+\frac{a_{23}(\theta )v_{3}(\theta )}{b_{23}(\theta )v_{3}(\theta )+v_{2}(\theta )}\\ & \;+\frac{a_{24}(\theta )v_{4}(\theta )}{b_{24}(\theta )v_{4}(\theta )+v_{2}(\theta )}+k_{2}(\theta )u_{2}(\theta )]\left|v_{2}(\theta )-v_{2}^{*}(\theta )\right|+v_{2}^{*}(\theta )[a_{22}(\theta )\left|v_{2}(\theta -\tau_{22})-v_{2}^{*}(\theta -\tau_{22})\right|\\ & \;+a_{21}(\theta )\left|v_{1}(\theta -\tau_{21})-v_{1}^{*}(\theta -\tau_{21})\right|+\frac{a_{23}(\theta )v_{2}^{*}(\theta )}{\left(b_{23}(\theta )v_{3}(\theta )+v_{2}(\theta ))(b_{23}(\theta )v_{3}^{*}(\theta )+v_{2}^{*}(\theta )\right)}\left|v_{3}(\theta )-v_{3}^{*}(\theta )\right|\\ & \;+(\frac{a_{23}(\theta )v_{3}^{*}(\theta )}{\left(b_{23}(\theta )v_{3}(\theta )+v_{2}(\theta ))(b_{23}(\theta )v_{3}^{*}(\theta )+v_{2}^{*}(\theta )\right)}+\frac{a_{24}(\theta )v_{4}^{*}(\theta )}{\left(b_{24}(\theta )v_{4}(\theta )+v_{2}(\theta ))(b_{24}(\theta )v_{4}^{*}(\theta )+v_{2}^{*}(\theta )\right)})\left|v_{2}(\theta )-v_{2}^{*}(\theta )\right|\\ & \;+\frac{a_{24}(\theta )v_{2}^{*}(\theta )}{\left(b_{24}(\theta )v_{4}(\theta )+v_{2}(\theta ))(b_{24}(\theta )v_{4}^{*}(\theta )+v_{2}^{*}(\theta )\right)}\left|v_{4}(\theta )-v_{4}^{*}(\theta )\right|+k_{2}(\theta )\left|u_{2}(\theta )-u_{2}^{*}(\theta )\right|]d\theta ]\\ & \;+a_{21}(t)\int_{t-\tau_{21}}^{t}[r_{1}(\theta )+a_{11}(\theta )v_{1}(\theta -\tau_{11})+a_{12}(\theta )v_{2}(\theta -\tau_{12})+\frac{a_{13}(\theta )v_{3}(\theta )}{b_{13}(\theta )v_{3}(\theta )+v_{1}(\theta )}+\frac{a_{14}(\theta )v_{4}(\theta )}{b_{14}(\theta )v_{4}(\theta )+v_{1}(\theta )}\\ & \;+k_{1}(\theta )u_{1}(\theta )]\left|v_{1}(\theta )-v_{1}^{*}(\theta )\right|+v_{1}^{*}(\theta )[a_{11}(\theta )\left|v_{1}(\theta -\tau_{11})-v_{1}^{*}(\theta -\tau_{11})\right|+a_{12}(\theta )\left|v_{2}(\theta -\tau_{12})-v_{2}^{*}(\theta -\tau_{12})\right|\\ & \;+\frac{a_{13}(\theta )v_{1}^{*}(\theta )}{\left(b_{13}(\theta )v_{3}(\theta )+v_{1}(\theta ))(b_{13}(\theta )v_{3}^{*}(\theta )+v_{1}^{*}(\theta )\right)}\left|v_{3}(\theta )-v_{3}^{*}(\theta )\right|\\ & \;+(\frac{a_{13}(\theta )v_{3}^{*}(\theta )}{\left(b_{13}(\theta )v_{3}(\theta )+v_{1}(\theta ))(b_{13}(\theta )v_{3}^{*}(\theta )+v_{1}^{*}(\theta )\right)}+\frac{a_{14}(\theta )v_{4}^{*}(\theta )}{\left(b_{14}(\theta )v_{4}(\theta )+v_{1}(\theta ))(b_{14}(\theta )v_{4}^{*}(\theta )+v_{1}^{*}(\theta )\right)})\left|v_{1}(\theta )-v_{1}^{*}(\theta )\right|\\ & \;+\frac{a_{14}(\theta )v_{1}^{*}(\theta )}{\left(b_{14}(\theta )v_{4}(\theta )+v_{1}(\theta ))(b_{14}(\theta )v_{4}^{*}(\theta )+v_{1}^{*}(\theta )\right)}\left|v_{4}(\theta )-v_{4}^{*}(\theta )\right|+k_{1}(\theta )\left|u_{1}(\theta )-u_{1}^{*}(\theta )\right|]d\theta ]. \end{array}$$

To eliminate the delay term, we redefine it again

(34) $$\begin{array}{ll} V_{22}(t) & =\int_{t-\tau_{22}}^{t}\int_{s}^{t}\left\{a_{22}(s+\tau_{22}\right)[r_{2}(\theta )+a_{22}(\theta )v_{2}(\theta -\tau_{22})+a_{21}(\theta )v_{1}(\theta -\tau_{21})+\frac{a_{23}(\theta )v_{3}(\theta )}{b_{23}(\theta )v_{3}(\theta )+v_{2}(\theta )}+\frac{a_{24}(\theta )v_{4}(\theta )}{b_{24}(\theta )v_{4}(\theta )+v_{2}(\theta )}\\ & \;+k_{2}(\theta )u_{2}(\theta )]\left|v_{2}(\theta )-v_{2}^{*}(\theta )\right|+v_{2}^{*}(\theta )[a_{22}(\theta )\left|v_{2}(\theta -\tau_{22})-v_{2}^{*}(\theta -\tau_{22})\right|+a_{21}(\theta )\left|v_{1}(\theta -\tau_{21})-v_{1}^{*}(\theta -\tau_{21})\right|\\ & \;+\frac{a_{23}(\theta )v_{2}^{*}(\theta )}{\left(b_{23}(\theta )v_{3}(\theta )+v_{2}(\theta ))(b_{23}(\theta )v_{3}^{*}(\theta )+v_{2}^{*}(\theta )\right)}]\left|v_{3}(\theta )-v_{3}^{*}(\theta )\right|+[\frac{a_{23}(\theta )v_{3}^{*}(\theta )}{\left(b_{23}(\theta )v_{3}(\theta )+v_{2}(\theta ))(b_{23}(\theta )v_{3}^{*}(\theta )+v_{2}^{*}(\theta )\right)}\\ & \;+\frac{a_{24}(\theta )v_{4}^{*}(\theta )}{\left(b_{24}(\theta )v_{4}(\theta )+v_{2}(\theta ))(b_{24}(\theta )v_{4}^{*}(\theta )+v_{2}^{*}(\theta )\right)}]\left|v_{2}(\theta )-v_{2}^{*}(\theta )\right|\\ & \;+\frac{a_{24}(\theta )v_{2}^{*}(\theta )}{\left(b_{24}(\theta )v_{4}(\theta )+v_{2}(\theta ))(b_{24}(\theta )v_{4}^{*}(\theta )+v_{2}^{*}(\theta )\right)}\left|v_{4}(\theta )-v_{4}^{*}(\theta )\right|+k_{2}(\theta )\left|u_{2}(\theta )-u_{2}^{*}(\theta )\right|]\}d\theta ds\\ & \;+\int_{t-\tau_{21}}^{t}\int_{s}^{t}\left\{a_{21}(s+\tau_{21}\right)[r_{1}(\theta )+a_{11}(\theta )v_{1}(\theta -\tau_{11})+a_{12}(\theta )v_{2}(\theta -\tau_{12})+\frac{a_{13}(\theta )v_{3}(\theta )}{b_{13}(\theta )v_{3}(\theta )+v_{1}(\theta )}\\ & \;+\frac{a_{14}(\theta )v_{4}(\theta )}{b_{14}(\theta )v_{4}(\theta )+v_{1}(\theta )}+k_{1}(\theta )u_{1}(\theta )]\left|v_{1}(\theta )-v_{1}^{*}(\theta )\right|+v_{1}^{*}(\theta )[a_{11}(\theta )\left|v_{1}(\theta -\tau_{11})-v_{1}^{*}(\theta -\tau_{11})\right|\\ & \;+a_{12}(\theta )\left|v_{2}(\theta -\tau_{12})-v_{2}^{*}(\theta -\tau_{12})\right|+\frac{a_{13}(\theta )v_{1}^{*}(\theta )}{\left(b_{13}(\theta )v_{3}(\theta )+v_{1}(\theta ))(b_{13}(\theta )v_{3}^{*}(\theta )+v_{1}^{*}(\theta )\right)}\left|v_{3}(\theta )-v_{3}^{*}(\theta )\right|\\ & \;+[\frac{a_{13}(\theta )v_{3}^{*}(\theta )}{\left(b_{13}(\theta )v_{3}(\theta )+v_{1}(\theta ))(b_{13}(\theta )v_{3}^{*}(\theta )+v_{1}^{*}(\theta )\right)}+\frac{a_{14}(\theta )v_{4}^{*}(\theta )}{\left(b_{14}(\theta )v_{4}(\theta )+v_{1}(\theta ))(b_{14}(\theta )v_{4}^{*}(\theta )+v_{1}^{*}(\theta )\right)}]\left|v_{1}(\theta )-v_{1}^{*}(\theta )\right|\\ & \;+\frac{a_{14}(\theta )v_{1}^{*}(\theta )}{\left(b_{14}(\theta )v_{4}(\theta )+v_{1}(\theta ))(b_{14}(\theta )v_{4}^{*}(\theta )+v_{1}^{*}(\theta )\right)}\left|v_{4}(\theta )-v_{4}^{*}(\theta )\right|+k_{1}(\theta )\left|u_{1}(\theta )-u_{1}^{*}(\theta )\right|]\}d\theta ds. \end{array}$$

From (33) and (34), one has

(35) $$\begin{array}{ll} D^{+}\sum_{i=1}^{2}V_{2i}(t) & \leq (\frac{a_{23}(t)v_{3}^{*}(t)}{\left(b_{23}(t)v_{3}(t)+v_{2}(t))(b_{23}(t)v_{3}^{*}(t)+v_{2}^{*}(t)\right)}+\frac{a_{24}(t)v_{4}^{*}(t)}{\left(b_{24}(t)v_{4}(t)+v_{2}(t))(b_{24}(t)v_{4}^{*}(t)+v_{2}^{*}(t)\right)}\\ & \;-a_{22}(t))\left|v_{2}(t)-v_{2}^{*}(t)\right|+a_{21}(t)\left|v_{1}(t)-v_{1}^{*}(t)\right|+\frac{a_{23}(t)v_{2}^{*}(t)}{\left(b_{23}(t)v_{3}(t)+v_{2}(t))(b_{23}(t)v_{3}^{*}(t)+v_{2}^{*}(t)\right)}\left|v_{3}(t)-v_{3}^{*}(t)\right|\\ & \;+\frac{a_{24}(t)v_{2}^{*}(t)}{\left(b_{24}(t)v_{4}(t)+v_{2}(t))(b_{24}(t)v_{4}^{*}(t)+v_{2}^{*}(t)\right)}\left|v_{4}(t)-v_{4}^{*}(t)\right|+k_{2}(t)\left|u_{2}(t)-u_{2}^{*}(t)\right|\\ & \;+\int_{t-\tau_{22}}^{t}a_{22}(s+\tau_{22})ds\{[r_{2}(t)+a_{22}(t)v_{2}(t-\tau_{22})+a_{21}(t)v_{1}(t-\tau_{21})+\frac{a_{23}(t)v_{3}(t)}{b_{23}(t)v_{3}(t)+v_{2}(t)}\\ & \;+\frac{a_{24}(t)v_{4}(t)}{b_{24}(t)v_{4}(t)+v_{2}(t)}+k_{2}(t)u_{2}(t)]\left|v_{2}(t)-v_{2}^{*}(t)\right|+v_{2}^{*}(t)[a_{22}(t)\left|v_{2}(t-\tau_{22})-v_{2}^{*}(t-\tau_{22})\right|\\ & \;+a_{21}(t)\left|v_{1}(t-\tau_{21})-v_{1}^{*}(t-\tau_{21})\right|+\frac{a_{23}(t)v_{2}^{*}(t)}{\left(b_{23}(t)v_{3}(t)+v_{2}(t))(b_{23}(t)v_{3}^{*}(t)+v_{2}^{*}(t)\right)}]\left|v_{3}(t)-v_{3}^{*}(t)\right|\\ & \;+v_{2}^{*}(t)[\frac{a_{23}(t)v_{3}^{*}(t)}{\left(b_{23}(t)v_{3}(t)+v_{2}(t))(b_{23}(t)v_{3}^{*}(t)+v_{2}^{*}(t)\right)}+\frac{a_{24}(t)v_{4}^{*}(t)}{\left(b_{24}(t)v_{4}(t)+v_{2}(t))(b_{14}(t)v_{4}^{*}(t)+v_{2}^{*}(t)\right)}]\left|v_{2}(t)-v_{2}^{*}(t)\right|\\ & \;+\frac{a_{24}(t){\left(v_{2}^{*}(t)\right)}^{2}}{\left(b_{24}(t)v_{4}(t)+v_{2}(t))(b_{24}(t)v_{4}^{*}(t)+v_{2}^{*}(t)\right)}\left|v_{4}(t)-v_{4}^{*}(t)\right|+v_{2}^{*}(t)k_{2}(t)\left|u_{2}(t)-u_{2}^{*}(t)\right|\}\\ & +\int_{t-\tau_{21}}^{t}a_{21}(s+\tau_{21})ds\{[r_{1}(t)+a_{11}(t)v_{1}(t-\tau_{11})+a_{12}(t)v_{2}(t-\tau_{12})+\frac{a_{13}(t)v_{3}(t)}{b_{13}(t)v_{3}(t)+v_{1}(t)}\\ & +\frac{a_{14}(t)v_{4}(t)}{b_{14}(t)v_{4}(t)+v_{1}(t)}+k_{1}(t)u_{1}(t)]\left|v_{1}(t)-v_{1}^{*}(t)\right|+v_{1}^{*}(t)[a_{11}(t)\left|v_{1}(t-\tau_{22})-v_{1}^{*}(t-\tau_{22})\right|\\ & +a_{12}(t)\left|v_{2}(t-\tau_{12})-v_{2}^{*}(t-\tau_{12})\right|+\frac{a_{13}(t)v_{1}^{*}(t)}{\left(b_{13}(t)v_{3}(t)+v_{1}(t))(b_{13}(t)v_{3}^{*}(t)+v_{1}^{*}(t)\right)}]\left|v_{3}(t)-v_{3}^{*}(t)\right|\\ & +v_{1}^{*}(t)[\frac{a_{13}(t)v_{3}^{*}(t)}{\left(b_{13}(t)v_{3}(t)+v_{1}(t))(b_{13}(t)v_{3}^{*}(t)+v_{1}^{*}(t)\right)}+\frac{a_{14}(t)v_{4}^{*}(t)}{\left(b_{14}(t)v_{4}(t)+v_{1}(t))(b_{14}(t)v_{4}^{*}(t)+v_{1}^{*}(t)\right)}]\left|v_{1}(t)-v_{1}^{*}(t)\right|\\ & +\frac{a_{14}(t){\left(v_{1}^{*}(t)\right)}^{2}}{\left(b_{14}(t)v_{4}(t)+v_{1}(t))(b_{14}(t)v_{4}^{*}(t)+v_{1}^{*}(t)\right)}\left|v_{4}(t)-v_{4}^{*}(t)\right|+v_{1}^{*}(t)k_{1}(t)\left|u_{1}(t)-u_{1}^{*}(t)\right|)\\ & \leq (\frac{a_{23}^{m}M_{3}}{{\left(b_{23}^{l}m_{3}+m_{2}\right)}^{2}}+\frac{a_{24}^{m}M_{4}}{{\left(b_{24}^{l}m_{4}+m_{2}\right)}^{2}}-a_{22}^{l})\left|v_{2}(t)-v_{2}^{*}(t)\right|+a_{21}^{m}\left|v_{1}(t)-v_{1}^{*}(t)\right|+\frac{a_{23}^{m}M_{2}}{{\left(b_{23}^{l}m_{3}+m_{2}\right)}^{2}}\left|v_{3}(t)-v_{3}^{*}(t)\right|\\ & +\frac{a_{24}^{m}M_{2}}{{\left(b_{24}^{l}m_{4}+m_{2}\right)}^{2}}\left|v_{4}(t)-v_{4}^{*}(t)\right|+k_{2}^{m}\left|u_{2}(t)-u_{2}^{*}(t)\right|+a_{22}^{m}\tau_{22}[r_{2}^{m}+a_{22}^{m}M_{2}+a_{21}^{m}M_{1}+\frac{a_{23}^{m}M_{3}}{b_{23}^{l}m_{3}+m_{2}}\\ & +\frac{a_{24}^{m}M_{4}}{b_{24}^{l}m_{4}+m_{2}}+k_{2}^{m}N_{2}]\left|v_{2}(t)-v_{2}^{*}(t)\right|+M_{2}{\left(a_{22}^{m}\right)}^{2}\tau_{22}\left|v_{2}(t-\tau_{22})-v_{2}^{*}(t-\tau_{22})\right|\\ & +M_{2}a_{21}^{m}a_{22}^{m}\tau_{22}\left|v_{1}(t-\tau_{21})-v_{1}^{*}(t-\tau_{21})\right|+\frac{a_{23}^{m}{\left(M_{2}\right)}^{2}a_{22}^{m}\tau_{22}}{{\left(b_{23}^{l}m_{3}+m_{2}\right)}^{2}}\left|v_{3}(t)-v_{3}^{*}(t)\right|+M_{2}a_{22}^{m}\tau_{22}(\frac{a_{23}^{m}M_{3}}{{\left(b_{23}^{l}m_{3}+m_{2}\right)}^{2}}\\ & +\frac{a_{24}^{m}M_{4}}{{\left(b_{24}^{l}m_{4}+m_{2}\right)}^{2}})\left|v_{2}(t)-v_{2}^{*}(t)\right|+\frac{a_{24}^{m}{\left(M_{2}\right)}^{2}a_{22}^{m}\tau_{22}}{{\left(b_{24}^{l}m_{4}+m_{2}\right)}^{2}}\left|v_{4}(t)-v_{4}^{*}(t)\right|+M_{2}k_{2}^{m}a_{22}^{m}\tau_{22}\left|u_{2}(t)-u_{2}^{*}(t)\right|\\ & +a_{21}^{m}\tau_{21}[r_{1}^{m}+a_{11}^{m}M_{1}+a_{12}^{m}M_{2}+\frac{a_{13}^{m}M_{3}}{b_{13}^{l}m_{3}+m_{1}}+\frac{a_{14}^{m}M_{4}}{b_{14}^{l}m_{4}+m_{1}}+k_{1}^{m}N_{1}]\left|v_{1}(t)-v_{1}^{*}(t)\right|\\ & +M_{1}a_{21}^{m}a_{11}^{m}\tau_{21}\left|v_{1}(t-\tau_{11})-v_{1}^{*}(t-\tau_{11})\right|+M_{1}a_{12}^{m}a_{21}^{m}\tau_{21}\left|v_{2}(t-\tau_{12})-v_{2}^{*}(t-\tau_{12})\right|\\ & +\frac{a_{13}^{m}{\left(M_{1}\right)}^{2}a_{21}^{m}\tau_{21}}{{\left(b_{13}^{l}m_{3}+m_{1}\right)}^{2}}\left|v_{3}(t)-v_{3}^{*}(t)\right|+M_{1}a_{21}^{m}\tau_{21}(\frac{a_{13}^{m}M_{3}}{{\left(b_{13}^{l}m_{3}+m_{1}\right)}^{2}}+\frac{a_{14}^{m}M_{4}}{{\left(b_{14}^{l}m_{4}+m_{1}\right)}^{2}})\left|v_{1}(t)-v_{1}^{*}(t)\right|\\ & +\frac{a_{14}^{m}{\left(M_{1}\right)}^{2}a_{21}^{m}\tau_{21}}{{\left(b_{14}^{l}m_{4}+m_{1}\right)}^{2}}\left|v_{4}(t)-v_{4}^{*}(t)\right|+M_{1}k_{1}^{m}a_{21}^{m}\tau_{21}\left|u_{1}(t)-u_{1}^{*}(t)\right|\\ & \leq \{\frac{a_{23}^{m}M_{3}}{{\left(b_{23}^{l}m_{3}+m_{2}\right)}^{2}}+\frac{a_{24}^{m}M_{4}}{{\left(b_{24}^{l}m_{4}+m_{2}\right)}^{2}}-a_{22}^{l}+a_{22}^{m}\tau_{22}[r_{2}^{m}+a_{22}^{m}M_{2}+a_{21}^{m}M_{1}+\frac{a_{23}^{m}M_{3}}{b_{23}^{l}m_{3}+m_{2}}+\frac{a_{24}^{m}M_{4}}{b_{24}^{l}m_{4}+m_{2}}+k_{2}^{m}N_{2}]\\ & +M_{2}a_{22}^{m}\tau_{22}(\frac{a_{23}^{m}M_{3}}{{\left(b_{23}^{l}m_{3}+m_{2}\right)}^{2}}+\frac{a_{24}^{m}M_{4}}{{\left(b_{24}^{l}m_{4}+m_{2}\right)}^{2}})\}\left|v_{2}(t)-v_{2}^{*}(t)\right|+\{a_{21}^{m}+a_{21}^{m}\tau_{21}[r_{1}^{m}+a_{11}^{m}M_{1}+a_{12}^{m}M_{2}\\ & +\frac{a_{13}^{m}M_{3}}{b_{13}^{l}m_{3}+m_{1}}+\frac{a_{14}^{m}M_{4}}{b_{14}^{l}m_{4}+m_{1}}+k_{1}^{m}N_{1}]+M_{1}a_{21}^{m}\tau_{21}(\frac{a_{13}^{m}M_{3}}{{\left(b_{13}^{l}m_{3}+m_{1}\right)}^{2}}+\frac{a_{14}^{m}M_{4}}{{\left(b_{14}^{l}m_{4}+m_{1}\right)}^{2}})\}\left|v_{1}(t)-v_{1}^{*}(t)\right|\\ & +(\frac{a_{23}^{m}M_{2}+a_{23}^{m}{\left(M_{2}\right)}^{2}a_{22}^{m}\tau_{22}}{{\left(b_{23}^{l}m_{3}+m_{2}\right)}^{2}}+\frac{a_{13}^{m}{\left(M_{1}\right)}^{2}a_{21}^{m}\tau_{21}}{{\left(b_{13}^{l}m_{3}+m_{1}\right)}^{2}})\left|v_{3}(t)-v_{3}^{*}(t)\right|\\ & +(\frac{a_{24}^{m}M_{2}+a_{24}^{m}{\left(M_{2}\right)}^{2}a_{22}^{m}\tau_{22}}{{\left(b_{24}^{l}m_{4}+m_{2}\right)}^{2}}+\frac{a_{14}^{m}{\left(M_{1}\right)}^{2}a_{21}^{m}\tau_{21}}{{\left(b_{14}^{l}m_{4}+m_{1}\right)}^{2}})\left|v_{4}(t)-v_{4}^{*}(t)\right|\\ & +k_{2}^{m}(1+M_{2}a_{22}^{m}\tau_{22})\left|u_{2}(t)-u_{2}^{*}(t)\right|+M_{1}k_{1}^{m}a_{21}^{m}\tau_{21}\left|u_{1}(t)-u_{1}^{*}(t)\right|\\ & +M_{2}{\left(a_{22}^{m}\right)}^{2}\tau_{22}\left|v_{2}(t-\tau_{22})-v_{2}^{*}(t-\tau_{22})\right|+M_{2}a_{21}^{m}a_{22}^{m}\tau_{22}\left|v_{1}(t-\tau_{21})-v_{1}^{*}(t-\tau_{21})\right|\\ & +M_{1}a_{21}^{m}a_{11}^{m}\tau_{21}\left|v_{1}(t-\tau_{11})-v_{1}^{*}(t-\tau_{11})\right|+M_{1}a_{12}^{m}a_{21}^{m}\tau_{21}\left|v_{2}(t-\tau_{12})-v_{2}^{*}(t-\tau_{12})\right|. \end{array}$$

Let

(36) $$\begin{array}{ll} V_{23}(t) & =M_{2}{\left(a_{22}^{m}\right)}^{2}\tau_{22}\int_{t-\tau_{22}}^{t}\left|v_{2}(w)-v_{2}^{*}(w)\right|dw+M_{2}a_{21}^{m}a_{22}^{m}\tau_{22}\int_{t-\tau_{21}}^{t}\left|v_{1}(w)-v_{1}^{*}(w)\right|dw\\ & \;+M_{1}a_{21}^{m}a_{11}^{m}\tau_{21}\int_{t-\tau_{11}}^{t}\left|v_{1}(w)-v_{1}^{*}(w)\right|dw+M_{1}a_{12}^{m}a_{21}^{m}\tau_{21}\int_{t-\tau_{12}}^{t}\left|v_{2}(w)-v_{2}^{*}(w)\right|dw, \end{array}$$

and

(37) $$V_{2}(t)=V_{21}(t)+V_{22}(t)+V_{23}(t).$$

By (35) and (36), we have

(38) $$\begin{array}{ll} D^{+}V_{2}(t) & \leq \{\frac{a_{23}^{m}M_{3}}{{\left(b_{23}^{l}m_{3}+m_{2}\right)}^{2}}+\frac{a_{24}^{m}M_{4}}{{\left(b_{24}^{l}m_{4}+m_{2}\right)}^{2}}-a_{22}^{l}+a_{22}^{m}\tau_{22}[r_{2}^{m}+a_{22}^{m}M_{2}+a_{21}^{m}M_{1}+\frac{a_{23}^{m}M_{3}}{b_{23}^{l}m_{3}+m_{2}}\\ & \;+\frac{a_{24}^{m}M_{4}}{b_{24}^{l}m_{4}+m_{2}}+k_{2}^{m}N_{2}]+M_{2}a_{22}^{m}\tau_{22}(\frac{a_{23}^{m}M_{3}}{{\left(b_{23}^{l}m_{3}+m_{2}\right)}^{2}}+\frac{a_{24}^{m}M_{4}}{{\left(b_{24}^{l}m_{4}+m_{2}\right)}^{2}})+M_{2}{\left(a_{22}^{m}\right)}^{2}\tau_{22}\\ & \;+M_{1}a_{12}^{m}a_{21}^{m}\tau_{21}\}\left|v_{2}(t)-v_{2}^{*}(t)\right|+\{a_{21}^{m}+a_{21}^{m}\tau_{21}[r_{1}^{m}+a_{11}^{m}M_{1}+a_{12}^{m}M_{2}+\frac{a_{13}^{m}M_{3}}{b_{13}^{l}m_{3}+m_{1}}\\ & \;+\frac{a_{14}^{m}M_{4}}{b_{14}^{l}m_{4}+m_{1}}+k_{1}^{m}N_{1}]+M_{1}a_{21}^{m}\tau_{21}(\frac{a_{13}^{m}M_{3}}{{\left(b_{13}^{l}m_{3}+m_{1}\right)}^{2}}+\frac{a_{14}^{m}M_{4}}{{\left(b_{14}^{l}m_{4}+m_{1}\right)}^{2}})+M_{2}a_{21}^{m}a_{22}^{m}\tau_{22}\\ & \;+M_{1}a_{21}^{m}a_{11}^{m}\tau_{21}\}\left|v_{1}(t)-v_{1}^{*}(t)\right|+(\frac{a_{23}^{m}M_{2}+a_{23}^{m}{\left(M_{2}\right)}^{2}a_{22}^{m}\tau_{22}}{{\left(b_{23}^{l}m_{3}+m_{2}\right)}^{2}}+\frac{a_{13}^{m}{\left(M_{1}\right)}^{2}a_{21}^{m}\tau_{21}}{{\left(b_{13}^{l}m_{3}+m_{1}\right)}^{2}})\left|v_{3}(t)-v_{3}^{*}(t)\right|\\ & \;+(\frac{a_{24}^{m}M_{2}+a_{24}^{m}{\left(M_{2}\right)}^{2}a_{22}^{m}\tau_{22}}{{\left(b_{24}^{l}m_{4}+m_{2}\right)}^{2}}+\frac{a_{14}^{m}{\left(M_{1}\right)}^{2}a_{21}^{m}\tau_{21}}{{\left(b_{14}^{l}m_{4}+m_{1}\right)}^{2}})\left|v_{4}(t)-v_{4}^{*}(t)\right|\\ & \;+k_{2}^{m}(1+M_{2}a_{22}^{m}\tau_{22})\left|u_{2}(t)-u_{2}^{*}(t)\right|+M_{1}k_{1}^{m}a_{21}^{m}\tau_{21}\left|u_{1}(t)-u_{1}^{*}(t)\right|. \end{array}$$

Analogously, we define

$$V_{31}(t)=\left|\ln v_{3}(t)-\ln v_{3}^{*}(t)\right|.$$

Let $D^{+}V_{31}(t)$ represent the right-hand derivative of $V_{31}(t)$, and it holds that

(39) $$\begin{array}{ll} D^{+}V_{31}(t) & =\mathrm{sgn}(v_{3}(t)-v_{3}^{*}(t))[a_{31}(t)(\frac{v_{1}(t-\tau_{31})}{b_{13}(t)v_{3}(t)+v_{1}(t-\tau_{31})}-\frac{v_{1}^{*}(t-\tau_{31})}{b_{13}(t)v_{3}^{*}(t)+v_{1}^{*}(t-\tau_{31})})\\ & \;+a_{32}(t)(\frac{v_{2}(t-\tau_{32})}{b_{23}(t)v_{3}(t)+v_{2}(t-\tau_{32})}-\frac{v_{2}^{*}(t-\tau_{32})}{b_{23}(t)v_{3}^{*}(t)+v_{2}^{*}(t-\tau_{32})}))\\ & \;-a_{34}(t)(v_{4}(t-\tau_{34})-v_{4}^{*}(t-\tau_{34}))+k_{3}(t)(u_{3}(t)-u_{3}^{*}(t))]\\ & =\mathrm{sgn}(v_{3}(t)-v_{3}^{*}(t))[a_{31}(t)b_{13}(t)\frac{v_{3}^{*}(t)(v_{1}(t-\tau_{31})-v_{1}^{*}(t-\tau_{31}))-v_{1}^{*}(t-\tau_{31})(v_{3}(t)-v_{3}^{*}(t))}{\left(b_{13}(t)v_{3}(t)+v_{1}(t-\tau_{31}))(b_{13}(t)v_{3}^{*}(t)+v_{1}^{*}(t-\tau_{31})\right)}\\ & \;+a_{32}(t)b_{23}(t)\frac{v_{3}^{*}(t)(v_{2}(t-\tau_{32})-v_{2}^{*}(t-\tau_{32}))-v_{2}^{*}(t-\tau_{32})(v_{3}(t)-v_{3}^{*}(t))}{\left(b_{23}(t)v_{3}(t)+v_{2}(t-\tau_{32}))(b_{23}(t)v_{3}^{*}(t)+v_{2}^{*}(t-\tau_{32})\right)}+k_{3}(t)(u_{3}(t)-u_{3}^{*}(t))\\ & \;-a_{34}(t)(v_{4}(t)-v_{4}^{*}(t))+a_{34}(t)\int_{t-\tau_{34}}^{t}({\dot{v}}_{4}(\theta )-{\dot{v}}_{4}^{*}(\theta ))d\theta \\ & =\mathrm{sgn}(v_{3}(t)-v_{3}^{*}(t))[a_{31}(t)b_{13}(t)\frac{v_{3}^{*}(t)(v_{1}(t-\tau_{31})-v_{1}^{*}(t-\tau_{31}))-v_{1}^{*}(t-\tau_{31})(v_{3}(t)-v_{3}^{*}(t))}{\left(b_{13}(t)v_{3}(t)+v_{1}(t-\tau_{31}))(b_{13}(t)v_{3}^{*}(t)+v_{1}^{*}(t-\tau_{31})\right)}\\ & \;+a_{32}(t)b_{23}(t)\frac{v_{3}^{*}(t)(v_{2}(t-\tau_{32})-v_{2}^{*}(t-\tau_{32}))-v_{2}^{*}(t-\tau_{32})(v_{3}(t)-v_{3}^{*}(t))}{\left(b_{23}(t)v_{3}(t)+v_{2}(t-\tau_{32}))(b_{23}(t)v_{3}^{*}(t)+v_{2}^{*}(t-\tau_{32})\right)}+k_{3}(t)(u_{3}(t)-u_{3}^{*}(t))\\ & \;-a_{34}(t)(v_{4}(t)-v_{4}^{*}(t))+a_{34}(t)\int_{t-\tau_{34}}^{t}\{v_{4}(\theta )[-r_{4}(\theta )+\frac{a_{41}(\theta )v_{1}(\theta -\tau_{41})}{b_{14}(\theta )v_{4}(\theta )+v_{1}(\theta -\tau_{41})}\\ & \;+\frac{a_{42}(\theta )v_{2}(\theta -\tau_{42})}{b_{24}(\theta )v_{4}(\theta )+v_{2}(\theta -\tau_{42})}-a_{43}(\theta )v_{3}(\theta -\tau_{43})+k_{4}(\theta )u_{4}(\theta )]-v_{4}^{*}(\theta )[-r_{4}(\theta )\\ & \;+\frac{a_{41}(\theta )v_{1}^{*}(\theta -\tau_{41})}{b_{14}(\theta )v_{4}^{*}(\theta )+v_{1}^{*}(\theta -\tau_{41})}+\frac{a_{42}(\theta )v_{2}^{*}(\theta -\tau_{42})}{b_{24}(\theta )v_{4}^{*}(\theta )+v_{2}^{*}(\theta -\tau_{42})}-a_{43}(\theta )v_{3}^{*}(\theta -\tau_{43})+k_{4}(\theta )u_{4}^{*}(\theta )]\}d\theta \\ & =\mathrm{sgn}(v_{3}(t)-v_{3}^{*}(t))[a_{31}(t)b_{13}(t)\frac{v_{3}^{*}(t)(v_{1}(t-\tau_{31})-v_{1}^{*}(t-\tau_{31}))-v_{1}^{*}(t-\tau_{31})(v_{3}(t)-v_{3}^{*}(t))}{\left(b_{13}(t)v_{3}(t)+v_{1}(t-\tau_{31}))(b_{13}(t)v_{3}^{*}(t)+v_{1}^{*}(t-\tau_{31})\right)}\\ & \;+a_{32}(t)b_{23}(t)\frac{v_{3}^{*}(t)(v_{2}(t-\tau_{32})-v_{2}^{*}(t-\tau_{32}))-v_{2}^{*}(t-\tau_{32})(v_{3}(t)-v_{3}^{*}(t))}{\left(b_{23}(t)v_{3}(t)+v_{2}(t-\tau_{32}))(b_{23}(t)v_{3}^{*}(t)+v_{2}^{*}(t-\tau_{32})\right)}+k_{3}(t)(u_{3}(t)-u_{3}^{*}(t))\\ & \;-a_{34}(t)(v_{4}(t)-v_{4}^{*}(t))+a_{34}(t)\int_{t-\tau_{34}}^{t}\{(v_{4}(\theta )-v_{4}^{*}(\theta ))[-r_{4}(\theta )+\frac{a_{41}(\theta )v_{1}(\theta -\tau_{41})}{b_{14}(\theta )v_{4}(\theta )+v_{1}(\theta -\tau_{41})}\\ & \;+\frac{a_{42}(\theta )v_{2}(\theta -\tau_{42})}{b_{24}(\theta )v_{4}(\theta )+v_{2}(\theta -\tau_{42})}-a_{43}(\theta )v_{3}(\theta -\tau_{43})+k_{4}(\theta )u_{4}(\theta )]\\ & \;-v_{4}^{*}(\theta )[a_{41}(\theta )b_{14}(\theta )\frac{v_{1}^{*}(\theta -\tau_{41})(v_{4}(\theta )-v_{4}^{*}(\theta ))-v_{4}^{*}(\theta )(v_{1}(\theta -\tau_{41})-v_{1}^{*}(\theta -\tau_{41}))}{\left(b_{14}(\theta )v_{4}^{*}(\theta )+v_{1}^{*}(\theta -\tau_{41}))(b_{14}(\theta )v_{4}(\theta )+v_{1}(\theta -\tau_{41})\right)}\\ & \;+a_{42}(\theta )b_{24}(\theta )\frac{v_{2}^{*}(\theta -\tau_{42})(v_{4}(\theta )-v_{4}^{*}(\theta ))-v_{4}^{*}(\theta )(v_{2}(\theta -\tau_{42})-v_{2}^{*}(\theta -\tau_{42}))}{\left(b_{24}(\theta )v_{4}^{*}(\theta )+v_{2}^{*}(\theta -\tau_{42}))((b_{14}(\theta )v_{4}(\theta )+v_{2}(\theta -\tau_{42})\right)}\\ & \;+a_{43}(\theta )(v_{3}(\theta -\tau_{43})-v_{3}^{*}(\theta -\tau_{43}))-k_{4}(\theta )(u_{4}(\theta )-u_{4}^{*}(\theta ))]\}d\theta \\ & \leq \frac{a_{31}(t)b_{13}(t)v_{3}^{*}(t)}{\left(b_{13}(t)v_{3}(t)+v_{1}(t-\tau_{31}))(b_{13}(t)v_{3}^{*}(t)+v_{1}^{*}(t-\tau_{31})\right)}\left|v_{1}(t-\tau_{31})-v_{1}^{*}(t-\tau_{31})\right|\\ & \;-[\frac{a_{31}(t)b_{13}(t)v_{2}^{*}(t-\tau_{32})}{\left(b_{13}(t)v_{3}(t)+v_{1}(t-\tau_{31}))(b_{13}(t)v_{3}^{*}(t)+v_{1}^{*}(t-\tau_{31})\right)}\\ & \;+\frac{a_{32}(t)b_{23}(t)v_{2}^{*}(t-\tau_{32})}{\left(b_{23}(t)v_{3}(t)+v_{2}(t-\tau_{32}))(b_{23}(t)v_{3}^{*}(t)+v_{2}^{*}(t-\tau_{32})\right)}\left|v_{3}(t)-v_{3}^{*}(t)\right|\\ & \;+\frac{a_{32}(t)b_{23}(t)v_{3}^{*}(t)}{\left(b_{23}(t)v_{3}(t)+v_{2}(t-\tau_{32}))(b_{23}(t)v_{3}^{*}(t)+v_{2}^{*}(t-\tau_{32})\right)}\left|v_{2}(t-\tau_{32})-v_{2}^{*}(t-\tau_{32})\right|\\ & \;+k_{3}(t)\left|u_{3}(t)-u_{3}^{*}(t)\right|+a_{34}(t)\left|v_{4}(t)-v_{4}^{*}(t)\right|\\ & \;+a_{34}(t)\int_{t-\tau_{34}}^{t}\{[r_{4}(\theta )+\frac{a_{41}(\theta )v_{1}(\theta -\tau_{41})}{b_{14}(\theta )v_{4}(\theta )+v_{1}(\theta -\tau_{41})}+\frac{a_{42}(\theta )v_{2}(\theta -\tau_{42})}{b_{24}(\theta )v_{4}(\theta )+v_{2}(\theta -\tau_{42})}\left|v_{4}(\theta )-v_{4}^{*}(\theta )\right|\\ & \;+a_{43}(\theta )v_{3}(\theta -\tau_{43})+k_{4}(\theta )u_{4}(\theta )]\left|v_{4}(\theta )-v_{4}^{*}(\theta )\right|\\ & \;+v_{4}^{*}(\theta )[\frac{a_{41}(\theta )b_{14}(\theta )v_{1}^{*}(\theta -\tau_{41})}{\left(b_{14}(\theta )v_{4}^{*}(\theta )+v_{1}^{*}(\theta -\tau_{41}))(b_{14}(\theta )v_{4}(\theta )+v_{1}(\theta -\tau_{41})\right)}\left|v_{4}(\theta )-v_{4}^{*}(\theta )\right|\\ & \;+\frac{a_{41}(\theta )b_{14}(\theta )v_{4}^{*}(\theta )}{\left(b_{14}(\theta )v_{4}^{*}(\theta )+v_{1}^{*}(\theta -\tau_{41}))(b_{14}(\theta )v_{4}(\theta )+v_{1}(\theta -\tau_{41})\right)}\left|v_{1}(\theta -\tau_{41})-v_{1}^{*}(\theta -\tau_{41})\right|\\ & \;+\frac{a_{42}(\theta )b_{24}(\theta )v_{2}^{*}(\theta -\tau_{42})}{\left(b_{24}(\theta )v_{4}^{*}(\theta )+v_{2}^{*}(\theta -\tau_{42}))((b_{14}(\theta )v_{4}(\theta )+v_{2}(\theta -\tau_{42})\right)}\left|v_{4}(\theta )-v_{4}^{*}(\theta )\right|\\ & \;+\frac{a_{42}(\theta )b_{24}(\theta )v_{4}^{*}(\theta )}{\left(b_{24}(\theta )v_{4}^{*}(\theta )+v_{2}^{*}(\theta -\tau_{42}))((b_{14}(\theta )v_{4}(\theta )+v_{2}(\theta -\tau_{42})\right)}\left|v_{2}(\theta -\tau_{42})-v_{2}^{*}(\theta -\tau_{42})\right|\\ & \;+a_{43}(\theta )\left|v_{3}(\theta -\tau_{43})-v_{3}^{*}(\theta -\tau_{43})\right|+k_{4}(\theta )\left|u_{4}(\theta )-u_{4}^{*}(\theta )\right|]\}d\theta . \end{array}$$

To eliminate the delay term, we redefine it again

(40) $$\begin{array}{ll} V_{32}(t) & =\int_{t-\tau_{34}}^{t}\int_{s}^{t}a_{34}(s+\tau_{34})\{[r_{4}(\theta )+\frac{a_{41}(\theta )v_{1}(\theta -\tau_{41})}{b_{14}(\theta )v_{4}(\theta )+v_{1}(\theta -\tau_{41})}+\frac{a_{42}(\theta )v_{2}(\theta -\tau_{42})}{b_{24}(\theta )v_{4}(\theta )+v_{2}(\theta -\tau_{42})}\\ & \;+a_{43}(\theta )v_{3}(\theta -\tau_{43})+k_{4}(\theta )u_{4}(\theta )]\left|v_{4}(\theta )-v_{4}^{*}(\theta )\right|\\ & \;+v_{4}^{*}(\theta )[\frac{a_{41}(\theta )b_{14}(\theta )v_{1}^{*}(\theta -\tau_{41})}{\left(b_{14}(\theta )v_{4}^{*}(\theta )+v_{1}^{*}(\theta -\tau_{41}))(b_{14}(\theta )v_{4}(\theta )+v_{1}(\theta -\tau_{41})\right)}\left|v_{4}(\theta )-v_{4}^{*}(\theta )\right|\\ & \;+\frac{a_{41}(\theta )b_{14}(\theta )v_{4}^{*}(\theta )}{\left(b_{14}(\theta )v_{4}^{*}(\theta )+v_{1}^{*}(\theta -\tau_{41}))(b_{14}(\theta )v_{4}(\theta )+v_{1}(\theta -\tau_{41})\right)}\left|v_{1}(\theta -\tau_{41})-v_{1}^{*}(\theta -\tau_{41})\right|\\ & \;+\frac{a_{42}(\theta )b_{24}(\theta )v_{2}^{*}(\theta -\tau_{42})}{\left(b_{24}(\theta )v_{4}^{*}(\theta )+v_{2}^{*}(\theta -\tau_{42}))((b_{14}(\theta )v_{4}(\theta )+v_{2}(\theta -\tau_{42})\right)}\left|v_{4}(\theta )-v_{4}^{*}(\theta )\right|\\ & \;+\frac{a_{42}(\theta )b_{24}(\theta )v_{4}^{*}(\theta )}{\left(b_{24}(\theta )v_{4}^{*}(\theta )+v_{2}^{*}(\theta -\tau_{42}))((b_{14}(\theta )v_{4}(\theta )+v_{2}(\theta -\tau_{42})\right)}\left|v_{2}(\theta -\tau_{42})-v_{2}^{*}(\theta -\tau_{42})\right|\\ & \;+a_{43}(\theta )\left|v_{3}(\theta -\tau_{43})-v_{3}^{*}(\theta -\tau_{43})\right|+k_{4}(\theta )\left|u_{4}(\theta )-u_{4}^{*}(\theta )\right|]\}d\theta ds. \end{array}$$

By (39) and (40), then

(41) $$\begin{array}{ll} D^{+}\sum_{i=1}^{2}V_{3i}(t) & \leq \frac{a_{31}(t)b_{13}(t)v_{3}^{*}(t)}{\left(b_{13}(t)v_{3}(t)+v_{1}(t-\tau_{31}))(b_{13}(t)v_{3}^{*}(t)+v_{1}^{*}(t-\tau_{31})\right)}\left|v_{1}(t-\tau_{31})-v_{1}^{*}(t-\tau_{31})\right|\\ & -[\frac{a_{31}(t)b_{13}(t)v_{2}^{*}(t-\tau_{32})}{\left(b_{13}(t)v_{3}(t)+v_{1}(t-\tau_{31}))(b_{13}(t)v_{3}^{*}(t)+v_{1}^{*}(t-\tau_{31})\right)}\\ & +\frac{a_{32}(t)b_{23}(t)v_{2}^{*}(t-\tau_{32})}{\left(b_{23}(t)v_{3}(t)+v_{2}(t-\tau_{32}))(b_{23}(t)v_{3}^{*}(t)+v_{2}^{*}(t-\tau_{32})\right)}\left|v_{3}(t)-v_{3}^{*}(t)\right|\\ & +\frac{a_{32}(t)b_{23}(t)v_{3}^{*}(t)}{\left(b_{23}(t)v_{3}(t)+v_{2}(t-\tau_{32}))(b_{23}(t)v_{3}^{*}(t)+v_{2}^{*}(t-\tau_{32})\right)}\left|v_{2}(t-\tau_{32})-v_{2}^{*}(t-\tau_{32})\right|\\ & +k_{3}(t)\left|u_{3}(t)-u_{3}^{*}(t)\right|+a_{34}(t)\left|v_{4}(t)-v_{4}^{*}(t)\right|\\ & +\int_{t-\tau_{34}}^{t}a_{34}(s+\tau_{34})ds\{[r_{4}(t)+\frac{a_{41}(t)v_{1}(t-\tau_{41})}{b_{14}(t)v_{4}(t)+v_{1}(t-\tau_{41})}+\frac{a_{42}(t)v_{2}(t-\tau_{42})}{b_{24}(t)v_{4}(t)+v_{2}(t-\tau_{42})}\\ & +a_{43}(t)v_{3}(t-\tau_{43})+k_{4}(t)u_{4}(t)]\left|v_{4}(t)-v_{4}^{*}(t)\right|\\ & +v_{4}^{*}(t)[\frac{a_{41}(t)b_{14}(t)v_{1}^{*}(t-\tau_{41})}{\left(b_{14}(t)v_{4}^{*}(t)+v_{1}^{*}(t-\tau_{41}))(b_{14}(t)v_{4}(t)+v_{1}(t-\tau_{41})\right)}\left|v_{4}(t)-v_{4}^{*}(t)\right|\\ & +\frac{a_{41}(t)b_{14}(t)v_{4}^{*}(t)}{\left(b_{14}(t)v_{4}^{*}(t)+v_{1}^{*}(t-\tau_{41}))(b_{14}(t)v_{4}(t)+v_{1}(t-\tau_{41})\right)}\left|v_{1}(t-\tau_{41})-v_{1}^{*}(t-\tau_{41})\right|\\ & +\frac{a_{42}(t)b_{24}(t)v_{2}^{*}(t-\tau_{42})}{\left(b_{24}(t)v_{4}^{*}(t)+v_{2}^{*}(t-\tau_{42}))((b_{14}(t)v_{4}(t)+v_{2}(t-\tau_{42})\right)}\left|v_{4}(t)-v_{4}^{*}(t)\right|\\ & +\frac{a_{42}(t)b_{24}(t)v_{4}^{*}(t)}{\left(b_{24}(t)v_{4}^{*}(t)+v_{2}^{*}(t-\tau_{42}))((b_{14}(t)v_{4}(t)+v_{2}(t-\tau_{42})\right)}\left|v_{2}(t-\tau_{42})-v_{2}^{*}(t-\tau_{42})\right|\\ & +a_{43}(t)\left|v_{3}(t-\tau_{43})-v_{3}^{*}(t-\tau_{43})\right|+k_{4}(t)\left|u_{4}(t)-u_{4}^{*}(t)\right|]\},\\ & \leq -[\frac{a_{31}^{l}b_{13}^{l}m_{2}}{{\left(b_{13}^{m}M_{3}+M_{1}\right)}^{2}}+\frac{a_{32}^{l}b_{23}^{l}m_{2}}{{\left(b_{23}^{m}M_{3}+M_{2}\right)}^{2}}\left|v_{3}(t)-v_{3}^{*}(t)\right|\\ & +\{a_{34}^{m}+a_{34}^{m}\tau_{34}(\frac{a_{41}^{m}b_{14}^{m}M_{1}M_{4}}{{\left(b_{14}^{l}m_{4}+m_{1}\right)}^{2}}+\frac{a_{42}^{m}b_{24}^{m}M_{2}M_{4}}{{\left(b_{24}^{l}m_{4}+m_{2}\right)}^{2}})+a_{34}^{m}\tau_{34}[r_{4}^{m}+\frac{a_{41}^{m}M_{1}}{b_{14}^{l}m_{4}+m_{1}}\\ & +\frac{a_{42}^{m}M_{2}}{b_{24}^{l}m_{4}+m_{2}}+a_{43}^{m}M_{3}+k_{4}^{m}N_{4}^{m}]\}\left|v_{4}(t)-v_{4}^{*}(t)\right|+k_{3}^{m}\left|u_{3}(t)-u_{3}^{*}(t)\right|\\ & +a_{34}^{m}\tau_{34}k_{4}^{m}M_{4}\left|u_{4}(t)-u_{4}^{*}(t)\right|+\frac{a_{31}^{m}b_{13}^{m}M_{3}}{{\left(b_{13}^{l}m_{3}+m_{1}\right)}^{2}}\left|v_{1}(t-\tau_{31})-v_{1}^{*}(t-\tau_{31})\right|\\ & +\frac{a_{32}^{m}b_{23}^{m}M_{3}}{{\left(b_{23}^{l}m_{3}+m_{2}\right)}^{2}}\left|v_{2}(t-\tau_{32})-v_{2}^{*}(t-\tau_{32})\right|\\ & +\frac{a_{34}^{m}\tau_{34}a_{41}^{m}b_{14}^{m}{\left(M_{4}\right)}^{2}}{{\left(b_{14}^{l}m_{4}+m_{1}\right)}^{2}}\left|v_{1}(t-\tau_{41})-v_{1}^{*}(t-\tau_{41})\right|\\ & +\frac{a_{34}^{m}\tau_{34}a_{42}^{m}b_{24}^{m}{\left(M_{4}\right)}^{2}}{{\left(b_{24}^{l}m_{4}+m_{2}\right)}^{2}}\left|v_{2}(t-\tau_{42})-v_{2}^{*}(t-\tau_{42})\right|\\ & +a_{34}^{m}\tau_{34}a_{43}^{m}M_{4}\left|v_{3}(t-\tau_{43})-v_{3}^{*}(t-\tau_{43})\right|. \end{array}$$

Let

(42) $$\begin{array}{ll} V_{33}(t) & =\frac{a_{31}^{m}b_{13}^{m}M_{3}}{{\left(b_{13}^{l}m_{3}+m_{1}\right)}^{2}}\int_{t-\tau_{31}}^{t}\left|v_{1}(w)-v_{1}^{*}(w)\right|dw+\frac{a_{32}^{m}b_{23}^{m}M_{3}}{{\left(b_{23}^{l}m_{3}+m_{2}\right)}^{2}}\int_{t-\tau_{32}}^{t}\left|v_{2}(w)-v_{2}^{*}(w)\right|dw\\ & +\frac{a_{34}^{m}\tau_{34}a_{41}^{m}b_{14}^{m}{\left(M_{4}\right)}^{2}}{{\left(b_{14}^{l}m_{4}+m_{1}\right)}^{2}}\int_{t-\tau_{41}}^{t}\left|v_{1}(w)-v_{1}^{*}(w)\right|dw+\frac{a_{34}^{m}\tau_{34}a_{42}^{m}b_{24}^{m}{\left(M_{4}\right)}^{2}}{{\left(b_{24}^{l}m_{4}+m_{2}\right)}^{2}}\int_{t-\tau_{42}}^{t}\left|v_{2}(w)-v_{2}^{*}(w)\right|dw\\ & +a_{34}^{m}\tau_{34}a_{43}^{m}M_{4}\int_{t-\tau_{43}}^{t}\left|v_{3}(w)-v_{3}^{*}(w)\right|dw, \end{array}$$

and

(43) $$V_{3}(t)=V_{31}(t)+V_{32}(t)+V_{33}(t).$$

By (41) and (42), we have

(44) $$\begin{array}{ll} D^{+}V_{3}(t) & \leq [a_{34}^{m}\tau_{34}a_{43}^{m}M_{4}-\frac{a_{31}^{l}b_{13}^{l}m_{2}}{{\left(b_{13}^{m}M_{3}+M_{1}\right)}^{2}}-\frac{a_{32}^{l}b_{23}^{l}m_{2}}{{\left(b_{23}^{m}M_{3}+M_{2}\right)}^{2}}]\left|v_{3}(t)-v_{3}^{*}(t)\right|\\ & +\{a_{34}^{m}+a_{34}^{m}\tau_{34}(\frac{a_{41}^{m}b_{14}^{m}M_{1}M_{4}}{{\left(b_{14}^{l}m_{4}+m_{1}\right)}^{2}}+\frac{a_{42}^{m}b_{24}^{m}M_{2}M_{4}}{{\left(b_{24}^{l}m_{4}+m_{2}\right)}^{2}})\\ & +a_{34}^{m}\tau_{34}[r_{4}^{m}+\frac{a_{41}^{m}M_{1}}{b_{14}^{l}m_{4}+m_{1}}+\frac{a_{42}^{m}M_{2}}{b_{24}^{l}m_{4}+m_{2}}+a_{43}^{m}M_{3}+k_{4}^{m}N_{4}^{m}]\}\left|v_{4}(t)-v_{4}^{*}(t)\right|\\ & +k_{3}^{m}\left|u_{3}(t)-u_{3}^{*}(t)\right|+a_{34}^{m}\tau_{34}k_{4}^{m}M_{4}\left|u_{4}(t)-u_{4}^{*}(t)\right|\\ & +[\frac{a_{31}^{m}b_{13}^{m}M_{3}}{{\left(b_{13}^{l}m_{3}+m_{1}\right)}^{2}}+\frac{a_{34}^{m}\tau_{34}a_{41}^{m}b_{14}^{m}{\left(M_{4}\right)}^{2}}{{\left(b_{14}^{l}m_{4}+m_{1}\right)}^{2}}]\left|v_{1}(t)-v_{1}^{*}(t)\right|\\ & +[\frac{a_{32}^{m}b_{23}^{m}M_{3}}{{\left(b_{23}^{l}m_{3}+m_{2}\right)}^{2}}+\frac{a_{34}^{m}\tau_{34}a_{42}^{m}b_{24}^{m}{\left(M_{4}\right)}^{2}}{{\left(b_{24}^{l}m_{4}+m_{2}\right)}^{2}}]\left|v_{2}(t)-v_{2}^{*}(t)\right|. \end{array}$$

Analogously, we define

$$V_{41}(t)=\left|\ln v_{4}(t)-\ln v_{4}^{*}(t)\right|.$$

Let $D^{+}V_{41}(t)$ indicate the right-hind derivative of $V_{41}(t)$, and it follows that

(45) $$\begin{array}{ll} D^{+}V_{41}(t) & =\mathrm{sgn}(v_{4}(t)-v_{4}^{*}(t))[a_{41}(t)(\frac{v_{1}(t-\tau_{41})}{b_{14}(t)v_{4}(t)+v_{1}(t-\tau_{41})}-\frac{v_{1}^{*}(t-\tau_{41})}{b_{14}(t)v_{4}^{*}(t)+v_{1}^{*}(t-\tau_{41})})\\ & \;+a_{42}(t)(\frac{v_{2}(t-\tau_{42})}{b_{24}(t)v_{4}(t)+v_{2}(t-\tau_{42})}-\frac{v_{2}^{*}(t-\tau_{42})}{b_{24}(t)v_{4}^{*}(t)+v_{2}^{*}(t-\tau_{42})}))\\ & \;-a_{43}(t)(v_{3}(t-\tau_{43})-v_{3}^{*}(t-\tau_{43}))+k_{4}(t)(u_{4}(t)-u_{4}^{*}(t))]\\ & =\mathrm{sgn}(v_{4}(t)-v_{4}^{*}(t))[a_{41}(t)b_{14}(t)\frac{v_{4}^{*}(t)(v_{1}(t-\tau_{41})-v_{1}^{*}(t-\tau_{41}))-v_{1}^{*}(t-\tau_{41})(v_{4}(t)-v_{4}^{*}(t))}{\left(b_{14}(t)v_{4}(t)+v_{1}(t-\tau_{41}))(b_{14}(t)v_{4}^{*}(t)+v_{1}^{*}(t-\tau_{41})\right)}\\ & \;+a_{42}(t)b_{24}(t)\frac{v_{4}^{*}(t)(v_{2}(t-\tau_{42})-v_{2}^{*}(t-\tau_{42}))-v_{2}^{*}(t-\tau_{42})(v_{4}(t)-v_{4}^{*}(t))}{\left(b_{24}(t)v_{4}(t)+v_{2}(t-\tau_{42}))(b_{24}(t)v_{4}^{*}(t)+v_{2}^{*}(t-\tau_{42})\right)}+k_{4}(t)(u_{4}(t)-u_{4}^{*}(t))\\ & \;-a_{43}(t)(v_{3}(t)-v_{3}^{*}(t))+a_{43}(t)\int_{t-\tau_{43}}^{t}({\dot{v}}_{3}(\theta )-{\dot{v}}_{3}^{*}(\theta ))d\theta \\ & =\mathrm{sgn}(v_{4}(t)-v_{4}^{*}(t))[a_{41}(t)b_{14}(t)\frac{v_{4}^{*}(t)(v_{1}(t-\tau_{41})-v_{1}^{*}(t-\tau_{41}))-v_{1}^{*}(t-\tau_{41})(v_{4}(t)-v_{4}^{*}(t))}{\left(b_{14}(t)v_{4}(t)+v_{1}(t-\tau_{41}))(b_{14}(t)v_{4}^{*}(t)+v_{1}^{*}(t-\tau_{41})\right)}\\ & \;+a_{42}(t)b_{24}(t)\frac{v_{4}^{*}(t)(v_{2}(t-\tau_{42})-v_{2}^{*}(t-\tau_{42}))-v_{2}^{*}(t-\tau_{42})(v_{4}(t)-v_{4}^{*}(t))}{\left(b_{24}(t)v_{4}(t)+v_{2}(t-\tau_{42}))(b_{24}(t)v_{4}^{*}(t)+v_{2}^{*}(t-\tau_{42})\right)}\\ & \;+k_{4}(t)(u_{4}(t)-u_{4}^{*}(t))-a_{43}(t)(v_{3}(t)-v_{3}^{*}(t))\\ & \;+a_{43}(t)\int_{t-\tau_{43}}^{t}\{v_{3}(\theta )[-r_{3}(\theta )+\frac{a_{31}(\theta )v_{1}(\theta -\tau_{31})}{b_{13}(\theta )v_{3}(\theta )+v_{1}(\theta -\tau_{31})}+\frac{a_{32}(\theta )v_{2}(\theta -\tau_{32})}{b_{23}(\theta )v_{3}(\theta )+v_{2}(\theta -\tau_{32})}\\ & \;-a_{34}(\theta )v_{4}(\theta -\tau_{34})+k_{3}(\theta )u_{3}(\theta )]-v_{3}^{*}(\theta )[-r_{3}(\theta )+\frac{a_{31}(\theta )v_{1}^{*}(\theta -\tau_{31})}{b_{13}(\theta )v_{3}^{*}(\theta )+v_{1}^{*}(\theta -\tau_{31})}\\ & \;+\frac{a_{32}(\theta )v_{2}^{*}(\theta -\tau_{32})}{b_{23}(\theta )v_{3}^{*}(\theta )+v_{2}^{*}(\theta -\tau_{32})}-a_{34}(\theta )v_{4}^{*}(\theta -\tau_{34})+k_{3}(\theta )u_{3}^{*}(\theta )]\}d\theta \\ & =\mathrm{sgn}(v_{4}(t)-v_{4}^{*}(t))[a_{41}(t)b_{14}(t)\frac{v_{4}^{*}(t)(v_{1}(t-\tau_{41})-v_{1}^{*}(t-\tau_{41}))-v_{1}^{*}(t-\tau_{41})(v_{4}(t)-v_{4}^{*}(t))}{\left(b_{14}(t)v_{4}(t)+v_{1}(t-\tau_{41}))(b_{14}(t)v_{4}^{*}(t)+v_{1}^{*}(t-\tau_{41})\right)}\\ & \;+a_{42}(t)b_{24}(t)\frac{v_{4}^{*}(t)(v_{2}(t-\tau_{42})-v_{2}^{*}(t-\tau_{42}))-v_{2}^{*}(t-\tau_{42})(v_{4}(t)-v_{4}^{*}(t))}{\left(b_{24}(t)v_{4}(t)+v_{2}(t-\tau_{42}))(b_{24}(t)v_{4}^{*}(t)+v_{2}^{*}(t-\tau_{42})\right)}\\ & \;+k_{4}(t)(u_{4}(t)-u_{4}^{*}(t))-a_{43}(t)(v_{3}(t)-v_{3}^{*}(t))\\ & \;+a_{43}(t)\int_{t-\tau_{43}}^{t}\{(v_{3}(\theta )-v_{3}^{*}(\theta ))[-r_{3}(\theta )+\frac{a_{31}(\theta )v_{1}(\theta -\tau_{31})}{b_{13}(\theta )v_{3}(\theta )+v_{1}(\theta -\tau_{31})}\\ & \;+\frac{a_{32}(\theta )v_{2}(\theta -\tau_{32})}{b_{23}(\theta )v_{3}(\theta )+v_{2}(\theta -\tau_{32})}-a_{34}(\theta )v_{4}(\theta -\tau_{34})+k_{3}(\theta )u_{3}(\theta )]\\ & \;-v_{3}^{*}(\theta )[a_{31}(\theta )b_{13}(\theta )\frac{v_{1}^{*}(\theta -\tau_{31})(v_{3}(\theta )-v_{3}^{*}(\theta ))-v_{3}^{*}(\theta )(v_{1}(\theta -\tau_{31})-v_{1}^{*}(\theta -\tau_{31}))}{\left(b_{13}(\theta )v_{3}^{*}(\theta )+v_{1}^{*}(\theta -\tau_{31}))(b_{13}(\theta )v_{3}(\theta )+v_{1}(\theta -\tau_{31})\right)}\\ & \;+a_{32}(\theta )b_{23}(\theta )\frac{v_{2}^{*}(\theta -\tau_{32})(v_{3}(\theta )-v_{3}^{*}(\theta ))-v_{3}^{*}(\theta )(v_{2}(\theta -\tau_{32})-v_{2}^{*}(\theta -\tau_{32}))}{\left(b_{23}(\theta )v_{3}^{*}(\theta )+v_{2}^{*}(\theta -\tau_{32}))((b_{13}(\theta )v_{3}(\theta )+v_{2}(\theta -\tau_{32})\right)}\\ & \;+a_{34}(\theta )(v_{4}(\theta -\tau_{34})-v_{4}^{*}(\theta -\tau_{34}))-k_{3}(\theta )(u_{3}(\theta )-u_{3}^{*}(\theta ))]\}d\theta \\ & \leq \frac{a_{41}(t)b_{14}(t)v_{4}^{*}(t)}{\left(b_{14}(t)v_{4}(t)+v_{1}(t-\tau_{41}))(b_{14}(t)v_{4}^{*}(t)+v_{1}^{*}(t-\tau_{41})\right)}\left|v_{1}(t-\tau_{41})-v_{1}^{*}(t-\tau_{41})\right|\\ & \;-[\frac{a_{41}(t)b_{14}(t)v_{2}^{*}(t-\tau_{42})}{\left(b_{14}(t)v_{4}(t)+v_{1}(t-\tau_{41}))(b_{14}(t)v_{4}^{*}(t)+v_{1}^{*}(t-\tau_{41})\right)}\\ & \;+\frac{a_{42}(t)b_{24}(t)v_{2}^{*}(t-\tau_{42})}{\left(b_{24}(t)v_{4}(t)+v_{2}(t-\tau_{42}))(b_{24}(t)v_{4}^{*}(t)+v_{2}^{*}(t-\tau_{42})\right)}\left|v_{4}(t)-v_{4}^{*}(t)\right|\\ & \;+\frac{a_{42}(t)b_{24}(t)v_{4}^{*}(t)}{\left(b_{24}(t)v_{4}(t)+v_{2}(t-\tau_{42}))(b_{24}(t)v_{4}^{*}(t)+v_{2}^{*}(t-\tau_{42})\right)}\left|v_{2}(t-\tau_{42})-v_{2}^{*}(t-\tau_{42})\right|\\ & \;+k_{4}(t)\left|u_{4}(t)-u_{4}^{*}(t)\right|+a_{43}(t)\left|v_{3}(t)-v_{3}^{*}(t)\right|\\ & \;+a_{43}(t)\int_{t-\tau_{43}}^{t}\{[r_{3}(\theta )+\frac{a_{31}(\theta )v_{1}(\theta -\tau_{31})}{b_{13}(\theta )v_{3}(\theta )+v_{1}(\theta -\tau_{31})}+\frac{a_{32}(\theta )v_{2}(\theta -\tau_{32})}{b_{23}(\theta )v_{3}(\theta )+v_{2}(\theta -\tau_{32})}\\ & \;+a_{34}(\theta )v_{4}(\theta -\tau_{34})+k_{3}(\theta )u_{3}(\theta )]\left|v_{3}(\theta )-v_{3}^{*}(\theta )\right|\\ & \;+v_{3}^{*}(\theta )[\frac{a_{31}(\theta )b_{13}(\theta )v_{1}^{*}(\theta -\tau_{31})}{\left(b_{13}(\theta )v_{3}^{*}(\theta )+v_{1}^{*}(\theta -\tau_{31}))(b_{13}(\theta )v_{3}(\theta )+v_{1}(\theta -\tau_{31})\right)}\left|v_{3}(\theta )-v_{3}^{*}(\theta )\right|\\ & \;+\frac{a_{31}(\theta )b_{13}(\theta )v_{3}^{*}(\theta )}{\left(b_{13}(\theta )v_{3}^{*}(\theta )+v_{1}^{*}(\theta -\tau_{31}))(b_{13}(\theta )v_{3}(\theta )+v_{1}(\theta -\tau_{31})\right)}\left|v_{1}(\theta -\tau_{31})-v_{1}^{*}(\theta -\tau_{31})\right|\\ & \;+\frac{a_{32}(\theta )b_{23}(\theta )v_{2}^{*}(\theta -\tau_{32})}{\left(b_{23}(\theta )v_{3}^{*}(\theta )+v_{2}^{*}(\theta -\tau_{32}))((b_{13}(\theta )v_{3}(\theta )+v_{2}(\theta -\tau_{32})\right)}\left|v_{3}(\theta )-v_{3}^{*}(\theta )\right|\\ & \;+\frac{a_{32}(\theta )b_{23}(\theta )v_{3}^{*}(\theta )}{\left(b_{23}(\theta )v_{3}^{*}(\theta )+v_{2}^{*}(\theta -\tau_{32}))((b_{13}(\theta )v_{3}(\theta )+v_{2}(\theta -\tau_{32})\right)}\left|v_{2}(\theta -\tau_{32})-v_{2}^{*}(\theta -\tau_{32})\right|\\ & \;+a_{34}(\theta )\left|v_{4}(\theta -\tau_{34})-v_{4}^{*}(\theta -\tau_{34})\right|+k_{3}(\theta )\left|u_{3}(\theta )-u_{3}^{*}(\theta )\right|]\}d\theta . \end{array}$$

To eliminate the delay term, we redefine it again

(46) $$\begin{array}{ll} V_{42}(t) & =\int_{t-\tau_{43}}^{t}\int_{s}^{t}a_{43}(s+\tau_{43})\{[r_{3}(\theta )+\frac{a_{31}(\theta )v_{1}(\theta -\tau_{31})}{b_{13}(\theta )v_{3}(\theta )+v_{1}(\theta -\tau_{31})}\\ & +\frac{a_{32}(\theta )v_{2}(\theta -\tau_{32})}{b_{23}(\theta )v_{3}(\theta )+v_{2}(\theta -\tau_{32})}+a_{34}(\theta )v_{4}(\theta -\tau_{34})+k_{3}(\theta )u_{3}(\theta )]\left|v_{3}(\theta )-v_{3}^{*}(\theta )\right|\\ & +v_{3}^{*}(\theta )[\frac{a_{31}(\theta )b_{13}(\theta )v_{1}^{*}(\theta -\tau_{31})}{\left(b_{13}(\theta )v_{3}^{*}(\theta )+v_{1}^{*}(\theta -\tau_{31}))(b_{13}(\theta )v_{3}(\theta )+v_{1}(\theta -\tau_{31})\right)}\left|v_{3}(\theta )-v_{3}^{*}(\theta )\right|\\ & +\frac{a_{31}(\theta )b_{13}(\theta )v_{3}^{*}(\theta )}{\left(b_{13}(\theta )v_{3}^{*}(\theta )+v_{1}^{*}(\theta -\tau_{31}))(b_{13}(\theta )v_{3}(\theta )+v_{1}(\theta -\tau_{31})\right)}\left|v_{1}(\theta -\tau_{31})-v_{1}^{*}(\theta -\tau_{31})\right|\\ & +\frac{a_{32}(\theta )b_{23}(\theta )v_{2}^{*}(\theta -\tau_{32})}{\left(b_{23}(\theta )v_{3}^{*}(\theta )+v_{2}^{*}(\theta -\tau_{32}))((b_{13}(\theta )v_{3}(\theta )+v_{2}(\theta -\tau_{32})\right)}\left|v_{3}(\theta )-v_{3}^{*}(\theta )\right|\\ & +\frac{a_{32}(\theta )b_{23}(\theta )v_{3}^{*}(\theta )}{\left(b_{23}(\theta )v_{3}^{*}(\theta )+v_{2}^{*}(\theta -\tau_{32}))((b_{13}(\theta )v_{3}(\theta )+v_{2}(\theta -\tau_{32})\right)}\left|v_{2}(\theta -\tau_{32})-v_{2}^{*}(\theta -\tau_{32})\right|\\ & +a_{34}(\theta )\left|v_{4}(\theta -\tau_{34})-v_{4}^{*}(\theta -\tau_{34})\right|+k_{3}(\theta )\left|u_{3}(\theta )-u_{3}^{*}(\theta )\right|]\}d\theta ds. \end{array}$$

From (45) and (46), then

(47) $$\begin{array}{ll} D^{+}\sum_{i=1}^{2}V_{4i}(t) & \leq \frac{a_{41}(t)b_{14}(t)v_{4}^{*}(t)}{\left(b_{14}(t)v_{4}(t)+v_{1}(t-\tau_{41}))(b_{14}(t)v_{4}^{*}(t)+v_{1}^{*}(t-\tau_{41})\right)}\left|v_{1}(t-\tau_{41})-v_{1}^{*}(t-\tau_{41})\right|\\ & \;-[\frac{a_{41}(t)b_{14}(t)v_{2}^{*}(t-\tau_{42})}{\left(b_{14}(t)v_{4}(t)+v_{1}(t-\tau_{41}))(b_{14}(t)v_{4}^{*}(t)+v_{1}^{*}(t-\tau_{41})\right)}\\ & \;+\frac{a_{42}(t)b_{24}(t)v_{2}^{*}(t-\tau_{42})}{\left(b_{24}(t)v_{4}(t)+v_{2}(t-\tau_{42}))(b_{24}(t)v_{4}^{*}(t)+v_{2}^{*}(t-\tau_{42})\right)}\left|v_{4}(t)-v_{4}^{*}(t)\right|\\ & \;+\frac{a_{42}(t)b_{24}(t)v_{4}^{*}(t)}{\left(b_{24}(t)v_{4}(t)+v_{2}(t-\tau_{42}))(b_{24}(t)v_{4}^{*}(t)+v_{2}^{*}(t-\tau_{42})\right)}\left|v_{2}(t-\tau_{42})-v_{2}^{*}(t-\tau_{42})\right|\\ & \;+k_{4}(t)\left|u_{4}(t)-u_{4}^{*}(t)\right|+a_{43}(t)\left|v_{3}(t)-v_{3}^{*}(t)\right|\\ & \;+\int_{t-\tau_{43}}^{t}a_{43}(s+\tau_{43})ds\{[r_{3}(t)+\frac{a_{31}(t)v_{1}(t-\tau_{31})}{b_{13}(t)v_{3}(t)+v_{1}(t-\tau_{31})}+\frac{a_{32}(t)v_{2}(t-\tau_{32})}{b_{23}(t)v_{3}(t)+v_{2}(t-\tau_{32})}\\ & \;+a_{34}(t)v_{4}(t-\tau_{34})+k_{3}(t)u_{3}(t)]\left|v_{3}(t)-v_{3}^{*}(t)\right|\\ & \;+v_{3}^{*}(t)[\frac{a_{31}(t)b_{13}(t)v_{1}^{*}(t-\tau_{31})}{\left(b_{13}(t)v_{3}^{*}(t)+v_{1}^{*}(t-\tau_{31}))(b_{13}(t)v_{3}(t)+v_{1}(t-\tau_{31})\right)}\left|v_{3}(t)-v_{3}^{*}(t)\right|\\ & \;+\frac{a_{31}(t)b_{13}(t)v_{3}^{*}(t)}{\left(b_{13}(t)v_{3}^{*}(t)+v_{1}^{*}(t-\tau_{31}))(b_{13}(t)v_{3}(t)+v_{1}(t-\tau_{31})\right)}\left|v_{1}(t-\tau_{31})-v_{1}^{*}(t-\tau_{31})\right|\\ & \;+\frac{a_{32}(t)b_{23}(t)v_{2}^{*}(t-\tau_{32})}{\left(b_{23}(t)v_{3}^{*}(t)+v_{2}^{*}(t-\tau_{32}))((b_{13}(t)v_{3}(t)+v_{2}(t-\tau_{32})\right)}\left|v_{3}(t)-v_{3}^{*}(t)\right|\\ & \;+\frac{a_{32}(t)b_{23}(t)v_{3}^{*}(t)}{\left(b_{23}(t)v_{3}^{*}(t)+v_{2}^{*}(t-\tau_{32}))((b_{13}(t)v_{3}(t)+v_{2}(t-\tau_{32})\right)}\left|v_{2}(t-\tau_{32})-v_{2}^{*}(t-\tau_{32})\right|\\ & \;+a_{34}(t)\left|v_{4}(t-\tau_{34})-v_{4}^{*}(t-\tau_{34})\right|+k_{3}(t)\left|u_{3}(t)-u_{3}^{*}(t)\right|]\},\\ & \leq -[\frac{a_{41}^{l}b_{14}^{l}m_{2}}{{\left(b_{14}^{m}M_{4}+M_{1}\right)}^{2}}+\frac{a_{42}^{l}b_{24}^{l}m_{2}}{{\left(b_{24}^{m}M_{4}+M_{2}\right)}^{2}}\left|v_{4}(t)-v_{4}^{*}(t)\right|\;+\{a_{43}^{m}+a_{43}^{m}\tau_{43}(\frac{a_{31}^{m}b_{13}^{m}M_{1}M_{3}}{{\left(b_{13}^{l}m_{3}+m_{1}\right)}^{2}}\\ & +\frac{a_{32}^{m}b_{23}^{m}M_{2}M_{3}}{{\left(b_{23}^{l}m_{3}+m_{2}\right)}^{2}})\;+a_{43}^{m}\tau_{43}[r_{3}^{m}+\frac{a_{31}^{m}M_{1}}{b_{13}^{l}m_{3}+m_{1}}+\frac{a_{32}^{m}M_{2}}{b_{23}^{l}m_{3}+m_{2}}+a_{34}^{m}M_{4}\\ & +k_{3}^{m}N_{3}^{m}]\}\left|v_{3}(t)-v_{3}^{*}(t)\right|\;+k_{4}^{m}\left|u_{4}(t)-u_{4}^{*}(t)\right|+a_{43}^{m}\tau_{43}k_{3}^{m}M_{3}\left|u_{3}(t)-u_{3}^{*}(t)\right|\\ & \;+\frac{a_{41}^{m}b_{14}^{m}M_{4}}{{\left(b_{14}^{l}m_{4}+m_{1}\right)}^{2}}\left|v_{1}(t-\tau_{41})-v_{1}^{*}(t-\tau_{41})\right|+\frac{a_{42}^{m}b_{24}^{m}M_{4}}{{\left(b_{24}^{l}m_{4}+m_{2}\right)}^{2}}\left|v_{2}(t-\tau_{42})-v_{2}^{*}(t-\tau_{42})\right|\\ & \;+\frac{a_{43}^{m}\tau_{43}a_{31}^{m}b_{13}^{m}{\left(M_{3}\right)}^{2}}{{\left(b_{13}^{l}m_{3}+m_{1}\right)}^{2}}\left|v_{1}(t-\tau_{31})-v_{1}^{*}(t-\tau_{31})\right|+\frac{a_{43}^{m}\tau_{43}a_{32}^{m}b_{23}^{m}{\left(M_{3}\right)}^{2}}{{\left(b_{23}^{l}m_{3}+m_{2}\right)}^{2}}\left|v_{2}(t-\tau_{32})-v_{2}^{*}(t-\tau_{32})\right|\\ & \;+a_{43}^{m}\tau_{43}a_{34}^{m}M_{3}\left|v_{3}(t-\tau_{34})-v_{3}^{*}(t-\tau_{34})\right|. \end{array}$$

Let

(48) $$\begin{array}{ll} V_{43}(t) & =\frac{a_{41}^{m}b_{14}^{m}M_{4}}{{\left(b_{13}^{l}m_{3}+m_{1}\right)}^{2}}\int_{t-\tau_{41}}^{t}\left|v_{1}(w)-v_{1}^{*}(w)\right|dw+\frac{a_{42}^{m}b_{24}^{m}M_{4}}{{\left(b_{24}^{l}m_{4}+m_{2}\right)}^{2}}\int_{t-\tau_{42}}^{t}\left|v_{2}(w)-v_{2}^{*}(w)\right|dw\\ & +\frac{a_{43}^{m}\tau_{43}a_{31}^{m}b_{13}^{m}{\left(M_{3}\right)}^{2}}{{\left(b_{13}^{l}m_{3}+m_{1}\right)}^{2}}\int_{t-\tau_{31}}^{t}\left|v_{1}(w)-v_{1}^{*}(w)\right|dw+\frac{a_{43}^{m}\tau_{43}a_{32}^{m}b_{23}^{m}{\left(M_{3}\right)}^{2}}{{\left(b_{23}^{l}m_{3}+m_{2}\right)}^{2}}\int_{t-\tau_{32}}^{t}\left|v_{2}(w)-v_{2}^{*}(w)\right|dw\\ & +a_{43}^{m}\tau_{43}a_{34}^{m}M_{3}\int_{t-\tau_{34}}^{t}\left|v_{3}(w)-v_{3}^{*}(w)\right|dw, \end{array}$$

and

(49) $$V_{4}(t)=V_{41}(t)+V_{42}(t)+V_{43}(t).$$

By (47) and (48), we have

(50) $$\begin{array}{ll} D^{+}V_{4}(t) & \leq [a_{43}^{m}\tau_{43}a_{34}^{m}M_{3}-\frac{a_{41}^{l}b_{14}^{l}m_{2}}{{\left(b_{14}^{m}M_{4}+M_{1}\right)}^{2}}-\frac{a_{42}^{l}b_{24}^{l}m_{2}}{{\left(b_{24}^{m}M_{4}+M_{2}\right)}^{2}}]\left|v_{4}(t)-v_{4}^{*}(t)\right|\\ & +\{a_{43}^{m}+a_{43}^{m}\tau_{43}(\frac{a_{31}^{m}b_{13}^{m}M_{1}M_{3}}{{\left(b_{13}^{l}m_{3}+m_{1}\right)}^{2}}+\frac{a_{32}^{m}b_{23}^{m}M_{2}M_{3}}{{\left(b_{23}^{l}m_{3}+m_{2}\right)}^{2}})\\ & +a_{43}^{m}\tau_{43}[r_{3}^{m}+\frac{a_{31}^{m}M_{1}}{b_{13}^{l}m_{3}+m_{1}}+\frac{a_{32}^{m}M_{2}}{b_{23}^{l}m_{3}+m_{2}}+a_{34}^{m}M_{4}+k_{3}^{m}N_{3}^{m}]\}\left|v_{3}(t)-v_{3}^{*}(t)\right|\\ & +k_{4}^{m}\left|u_{4}(t)-u_{4}^{*}(t)\right|+a_{43}^{m}\tau_{43}k_{3}^{m}M_{3}\left|u_{3}(t)-u_{3}^{*}(t)\right|+[\frac{a_{41}^{m}b_{14}^{m}M_{4}}{{\left(b_{14}^{l}m_{4}+m_{1}\right)}^{2}}+\frac{a_{43}^{m}\tau_{43}a_{31}^{m}b_{13}^{m}{\left(M_{3}\right)}^{2}}{{\left(b_{13}^{l}m_{3}+m_{1}\right)}^{2}}]\\ & \cdot \left|v_{1}(t)-v_{1}^{*}(t)\right|+[\frac{a_{42}^{m}b_{24}^{m}M_{4}}{{\left(b_{24}^{l}m_{4}+m_{2}\right)}^{2}}+\frac{a_{43}^{m}\tau_{43}a_{32}^{m}b_{23}^{m}{\left(M_{3}\right)}^{2}}{{\left(b_{23}^{l}m_{3}+m_{2}\right)}^{2}}]\left|v_{2}(t)-v_{2}^{*}(t)\right|. \end{array}$$

Define

$$\begin{array}{l} V_{5}(t)=\left|\ln u_{1}(t)-\ln u_{1}^{*}(t)\right|,\;V_{6}(t)=\left|\ln u_{2}(t)-\ln u_{2}^{*}(t)\right|,\;\\ V_{7}(t)=\left|\ln u_{3}(t)-\ln u_{3}^{*}(t)\right|,\;V_{8}(t)=\left|\ln u_{4}(t)-\ln u_{4}^{*}(t)\right|, \end{array}$$

and calculate the right-hind derivative of $V_{5}(t),\;V_{6}(t),\;V_{7}(t),\;V_{8}(t),$ along system (3), it follows that

(51) $$\begin{array}{ll} D^{+}V_{5}(t) & \leq \mathrm{sgn}(u_{1}(t)-u_{1}^{*}(t))[-f_{1}(t)(u_{1}(t)-u_{1}^{*}(t))+q_{1}(t)(v_{1}(t)-v_{1}^{*}(t))]\\ & \leq -f_{1}^{l}\left|u_{1}(t)-u_{1}^{*}(t)\right|+q_{1}^{m}\left|v_{1}(t)-v_{1}^{*}(t)\right|. \end{array}$$

(52) $$\begin{array}{ll} D^{+}V_{6}(t) & \leq \mathrm{sgn}(u_{2}(t)-u_{2}^{*}(t))[-f_{2}(t)(u_{2}(t)-u_{2}^{*}(t))+q_{2}(t)(v_{2}(t)-v_{2}^{*}(t))]\\ & \leq -f_{2}^{l}\left|u_{2}(t)-u_{2}^{*}(t)\right|+q_{2}^{m}\left|v_{2}(t)-v_{2}^{*}(t)\right|. \end{array}$$

(53) $$\begin{array}{ll} D^{+}V_{7}(t) & \leq \mathrm{sgn}(u_{3}(t)-u_{3}^{*}(t))[-f_{3}(t)(u_{3}(t)-u_{3}^{*}(t))-q_{3}(t)(v_{3}(t)-v_{3}^{*}(t))]\\ & \leq -f_{3}^{l}\left|u_{3}(t)-u_{3}^{*}(t)\right|+q_{3}^{m}\left|v_{3}(t)-v_{3}^{*}(t)\right|. \end{array}$$

and

(54) $$\begin{array}{ll} D^{+}V_{8}(t) & \leq \mathrm{sgn}(u_{4}(t)-u_{4}^{*}(t))[-f_{4}(t)(u_{4}(t)-u_{4}^{*}(t))-q_{4}(t)(v_{4}(t)-v_{4}^{*}(t))]\\ & \leq -f_{4}^{l}\left|u_{4}(t)-u_{4}^{*}(t)\right|+q_{4}^{m}\left|v_{4}(t)-v_{4}^{*}(t)\right|. \end{array}$$

Furthermore, we delineate a Lyapunov function in the manner outlined below.

$$V(t)=\sum_{i=1}^{8}V_{i}(t).$$

Then, from (32), (38), (44), and (50)–(54), we finally can obtain for all t≥T+τ

(55) $$D^{+}V(t)\leq -\sum_{i=1}^{4}A_{i}(t)\left|v_{i}(t)-v_{i}^{*}(t)\right|-\sum_{i=1}^{4}B_{i}(t)\left|u_{i}(t)-u_{i}^{*}(t)\right|.$$

From assumption $\left(H_{11})-(H_{18}\right)$, there exists a constant α>0 and $\omega^{*}>\omega +\tau$ such that for all $t\geq \omega^{*}$, we have

(56) $$A_{i}(t)\geq \alpha >0,\;B_{i}(t)\geq \alpha >0,\;i=1,2,3,4.$$

Integrating both sides of (55) on interval $\left[\omega^{*},\;t\right]$ and by (56), we have

(57) $$V(t)+\alpha \int_{\omega^{*}}^{t}(\sum_{i=1}^{4}[\left|v_{i}(s)-v_{i}^{*}(s)\right|+\left|u_{i}(s)-u_{i}^{*}(s)\right|])ds\leq V(\omega^{*})<+\infty .$$

Hence, V(t) is bounded on $[\omega^{*},+\infty ),$ and

(58) $$\int_{\omega^{*}}^{\infty }(\sum_{i=1}^{4}[\left|v_{i}(t)-v_{i}^{*}(t)\right|+\left|u_{i}(t)-u_{i}^{*}(t)\right|])ds\leq \frac{V(\omega^{*})}{\alpha }<+\infty .$$

Therefore

(59) $$\sum_{i=1}^{4}[\left|v_{i}(t)-v_{i}^{*}(t)\right|+\left|u_{i}(t)-u_{i}^{*}(t)\right|]\in L^{1}(\omega^{*},+\infty ).$$

From the uniform permanence of model (3), we can obtain that $\left|v_{i}(t)-v_{i}^{*}(t)\right|,\;\left|u_{i}(t)-u_{i}^{*}(t)\right|$ and their derivatives remain bounded on $\left[\omega^{*},+\infty \right)$. Thus, $\left|z_{i}(t)-z_{i}^{*}(t)\right|,u_{i}(t)-u_{i}^{*}(t)$ are uniformly continuous on $\left[\omega^{*},+\infty \right)$. By Lemma 8.2 in [44], we can conclude that

$$\underset{t\to \infty }{\lim\;}\left|v_{i}(t)-v_{i}^{*}(t)\right|=0,\;\underset{t\to \infty }{\lim\;}\left|u_{i}(t)-u_{i}^{*}(t)\right|=0,\;i=1,2,3,4.$$

From (25) and the squeeze theorem, (24) holds true. That is, (1) and (2) have a spatial homogeneity strictly positive and globally asymptotically stable ω-periodic solution $(v_{1}^{*}(t),v_{2}^{*}(t),v_{3}^{*}(t),v_{4}^{*}(t),u_{1}^{*}(t),$ see (Definition 2.3, [41]). The proof of Theorem 4 is now completed. □

**Theorem 5.**  *Assume that the* ω- *periodic system (1) fulfills prerequisites* $\left(H_{1})-(H_{18}\right)$ *, then the system (1) is permanent, i.e., the solution* $\left(v_{1}(x,t),v_{2}(x,t),v_{3}(x,t),v_{4}(x,t),u_{1}(x,t),u_{2}(x,t),u_{3}(x,t),u_{4}(x,t)\right)$ *of system (1) and (2) with any positive IC satisfies*

(60) $$m_{1}\leq v_{i}(x,t)\leq M_{1},n_{i}\leq u_{i}(x,t)\leq N_{i},\;uniformly\;for\;\left(x,t)\in \bar{\Omega }\times (T,+\infty \right).$$

**Proof.**  From Theorem 2, it holds that

(61) $$m_{i}\leq v_{i}^{*}(t)=v_{i}^{*}(t+\omega )\leq M_{i},\;n_{i}\leq u_{i}^{*}(t)=u_{i}^{*}(t+\omega )\leq N_{i},\;t\in [-\tau ,+\infty ),$$

where $M_{i},N_{i},n_{i}$ and $m_{i}$ are positive real numbers. Moreover, from Theorem 4, one has

(62) $$\underset{t\to +\infty }{\lim\;}\left|v_{i}(x,t)-v_{i}^{*}(t)\right|=0,\;\underset{t\to +\infty }{\lim\;}\left|u_{i}(x,t)-u_{i}^{*}(t)\right|=0,\;uniformly\;for\;x\in \bar{\Omega }.$$

Therefore, by (61) and (62), there exists a positive real number T such that the solution $\left(v_{1}(x,t),v_{2}(x,t),v_{3}(x,t),v_{4}(x,t),u_{1}(x,t),u_{2}(x,t),u_{3}(x,t),u_{4}(x,t)\right)$ of system (1) and (2) with any positive initial values fulfills

$$m_{i}\leq v_{i}(x,t)\leq M_{i},n_{i}\leq u_{i}(x,t)\leq N_{i},i=1,2,3,4,\;uniformly\;for\;\left(x,t)\in \bar{\Omega }\times (T,+\infty \right).$$

The proof of Theorem 5 is now completed. □

## 4. Numerical Example

**Example 1**. Taking into account the provided four-species periodic diffusive predator–prey models that incorporate delay and feedback control, we determine specific parameter values for the models presented in equations (63) and (64) based on the prerequisites stated in Theorem 4 and subsequent computations. It is important to emphasize that the selection of these parameters is not exclusive.

(63) $$\left\{\begin{array}{ll} \frac{\partial v_{1}(x,t)}{\partial t}-\frac{\partial^{2}v_{1}(x,t)}{\partial x^{2}} & =v_{1}(x,t)[(22+\cos\pi t)-(10+\sin\pi t)v_{1}(x,t-0.001)-(0.06+0.01\sin\pi t)v_{2}(x,t-0.004)\\ & -\frac{\left(0.04+0.01\sin\pi t)v_{3}(x,t\right)}{\left(2.1+0.1\sin\pi t)v_{3}(x,t)+v_{1}(x,t\right)}-\frac{\left(0.05+0.01\sin\pi t)v_{4}(x,t\right)}{\left(2+0.1\sin\pi t)v_{4}(x,t)+v_{1}(x,t\right)}-(0.05+0.01\sin\pi t)u_{1}(x,t)],\\ \frac{\partial v_{2}(x,t)}{\partial t}-\frac{\partial^{2}v_{2}(x,t)}{\partial x^{2}} & =v_{2}(x,t)[(23+\cos\pi t)-(11+\sin\pi t)v_{2}(x,t-0.002)-(0.05+0.01\sin\pi t)v_{1}(x,t-0.003)\\ & -\frac{\left(0.05+0.01\sin\pi t)v_{3}(x,t\right)}{\left(2.1+0.01\sin\pi t)v_{3}(x,t)+v_{2}(x,t\right)}-\frac{\left(0.04+0.01\sin\pi t)v_{4}(x,t\right)}{\left(2+0.1\sin\pi t)v_{4}(x,t)+v_{2}(x,t\right)}-(0.06+0.01\sin\pi t)u_{2}(x,t)],\\ \frac{\partial v_{3}(x,t)}{\partial t}-\frac{\partial^{2}v_{3}(x,t)}{\partial x^{2}} & =v_{3}(x,t)[-(10.5+0.1\cos\pi t)+\frac{\left(20+\sin\pi t)v_{1}(x,t-0.007\right)}{\left(2.1+0.1\sin\pi t)v_{3}(x,t)+v_{1}(x,t-0.007\right)}\\ & +\frac{\left(1.5+0.1\sin\pi t)v_{2}(x,t-0.008\right)}{\left(2.1+0.1\sin\pi t)v_{3}(x,t)+v_{2}(x,t-0.008\right)}-(0.05+0.01\sin\pi t)v_{4}(x,t-0.005)\\ & +(0.05+0.01\sin\pi t)u_{3}(x,t)],\\ \frac{\partial v_{4}(x,t)}{\partial t}-\frac{\partial^{2}v_{4}(x,t)}{\partial x^{2}} & =v_{4}(x,t)[-(11+0.1\cos\pi t)+\frac{\left(21+\sin\pi t)v_{1}(x,t-0.009\right)}{\left(2+0.1\sin\pi t)v_{4}(x,t)+v_{1}(x,t-0.009\right)}\\ & +\frac{\left(1.5+0.1\sin\pi t)v_{2}(x,t-0.01\right)}{\left(2+0.1\sin\pi t)v_{4}(x,t)+v_{2}(x,t-0.01\right)}-(0.04+0.01\sin\pi t)v_{3}(x,t-0.006)\\ & +(0.04+0.01\sin\pi t)u_{4}(x,t)],\\ \;\;\;\;\;\frac{\partial u_{1}(x,t)}{\partial t} & =(21+\sin\pi t)-(10+0.5\sin\pi t)u_{1}(x,t)+(0.05+0.01\sin\pi t)v_{1}(x,t),\\ \;\;\;\;\;\frac{\partial u_{2}(x,t)}{\partial t} & =(21.5+\sin\pi t)-(9.5+0.5\sin\pi t)u_{2}(x,t)+(0.04+0.01\sin\pi t)v_{2}(x,t),\\ \;\;\;\;\;\frac{\partial u_{3}(x,t)}{\partial t} & =(19.5+\sin\pi t)-(9.5+0.5\sin\pi t)u_{3}(x,t)-(0.05+0.01\sin\pi t)v_{3}(x,t),\\ \;\;\;\;\;\frac{\partial u_{4}(x,t)}{\partial t} & =(20+\sin\pi t)-(10+0.5\sin\pi t)u_{4}(x,t)-(0.04+0.01\sin\pi t)v_{4}(x,t), \end{array}\right.$$

with boundary and initial conditions

(64) $$\left\{\begin{array}{l} \partial v_{i}(x,t)/\partial n=\partial u_{i}(x,t)/\partial n=0,\;(x,t)\in \{0,2\pi \}\times [0,+\infty ),\;i=1,2,3,4,\\ v_{1}(x,t)=(2+2t)[1+\cos(x+\pi )],v_{2}(x,t)=(2.5+2.5t)[1+\cos(x+\pi )],\\ v_{3}(x,t)=(1+2t)[1+\sin(x-\frac{\pi }{2})],v_{4}(x,t)=(0.9+1.5t)[1+\sin(x-\frac{\pi }{2})],\\ u_{1}(x,t)=1.5\sin(x-\frac{\pi }{2})],u_{2}(x,t)=\sin(x-\frac{\pi }{2})],\\ u_{3}(x,t)=0.7\sin(x-\frac{\pi }{2})],\;u_{4}(x,t)=0.3\sin(x-\frac{\pi }{2})],\\ (x,t)\in (0,2\pi )\times [-0.005,0]. \end{array}\right.$$

By calculating, we have

$$\begin{array}{c} M_{1}=\frac{r_{1}^{m}}{a_{11}^{l}}\exp(r_{1}^{m}\tau_{11})\approx 2.6150,M_{2}=\frac{r_{2}^{m}}{a_{22}^{l}}\exp(r_{2}^{m}\tau_{22})\approx 2.5180,\\ N_{1}=\frac{e_{1}^{m}+q_{1}^{m}M_{1}}{f_{1}^{l}}\approx 2.3370,N_{2}=\frac{e_{2}^{m}+q_{2}^{m}M_{2}}{f_{2}^{l}}\approx 2.5175,N_{3}=\frac{e_{3}^{m}}{f_{3}^{l}}\approx 2.3370,N_{4}=\frac{e_{4}^{m}}{f_{4}^{l}}\approx 2.2105, \end{array}$$

$$\begin{array}{c} M_{3}^{*}=\frac{M_{1}(a_{31}^{m}-r_{3}^{l}+a_{32}^{m}+k_{3}^{m}N_{3})}{b_{13}^{l}(r_{3}^{l}-a_{32}^{m}-k_{3}^{m}N_{3})}\approx 1.8619,M_{4}^{*}=\frac{M_{1}(a_{41}^{m}-r_{4}^{l}+a_{42}^{m}+k_{4}^{m}N_{4})}{b_{14}^{l}(r_{4}^{l}-a_{42}^{m}-k_{4}^{m}N_{4})}\approx 1.9186,\\ m_{1}=\frac{(r_{1}^{l}-a_{12}^{m}M_{2}-k_{1}^{m}N_{1})b_{13}^{l}b_{14}^{l}-a_{13}^{m}b_{14}^{l}-a_{14}^{m}b_{13}^{l}}{b_{13}^{l}b_{14}^{l}a_{11}^{m}}\exp[(\frac{(r_{1}^{l}-a_{12}^{m}M_{2}-k_{1}^{m}N_{1})b_{13}^{l}b_{14}^{l}-a_{13}^{m}b_{14}^{l}-a_{14}^{m}b_{13}^{l}}{b_{13}^{l}b_{14}^{l}}-a_{11}^{m}M_{1})\tau_{11}]\approx 1.8600,\\ m_{2}=\frac{(r_{2}^{l}-a_{21}^{m}M_{1}-k_{2}^{m}N_{2})b_{23}^{l}b_{24}^{l}-a_{23}^{m}b_{24}^{l}-a_{24}^{m}b_{23}^{l}}{b_{23}^{l}b_{24}^{l}a_{22}^{m}}\exp[(\frac{(r_{2}^{l}-a_{21}^{m}M_{1}-k_{2}^{m}N_{2})b_{23}^{l}b_{24}^{l}-a_{23}^{m}b_{24}^{l}-a_{24}^{m}b_{23}^{l}}{b_{23}^{l}b_{24}^{l}}-a_{22}^{m}M_{2})\tau_{22}]\approx 1.7702,\\ m_{3}^{*}=\frac{a_{31}^{l}m_{1}-M_{1}(r_{3}^{m}+a_{34}^{m}M_{4}-k_{3}^{l}n_{3})}{b_{13}^{m}(r_{3}^{m}+a_{34}^{m}M_{4}-k_{3}^{l}n_{3})}\approx 0.3208,m_{4}^{*}=\frac{a_{41}^{l}m_{1}-M_{1}(r_{4}^{m}+a_{43}^{m}M_{3}-k_{4}^{l}n_{4})}{b_{14}^{m}(r_{4}^{m}+a_{43}^{m}M_{3}-k_{4}^{l}n_{4})}\approx 0.3449. \end{array}$$

$$\begin{array}{c} r_{3}^{l}-a_{32}^{m}-k_{3}^{m}N_{3}\approx 8.6633>0,\;r_{4}^{l}-a_{42}^{m}-k_{4}^{m}N_{4}\approx 9.1895>0,\\ a_{31}^{m}-r_{3}^{l}+a_{32}^{m}+k_{3}^{m}N_{3}\approx 12.3367>0,\;a_{41}^{m}-r_{4}^{l}+a_{42}^{m}+k_{4}^{m}N_{4}\approx 12.8105>0,\\ (r_{1}^{l}-a_{12}^{m}M_{2}-k_{1}^{m}N_{1})b_{13}^{l}b_{14}^{l}-a_{13}^{m}b_{14}^{l}-a_{14}^{m}b_{13}^{l}\approx 78.3834>0,\;\\ (r_{2}^{l}-a_{21}^{m}M_{1}-k_{2}^{m}N_{2})b_{23}^{l}b_{24}^{l}-a_{23}^{m}b_{24}^{l}-a_{24}^{m}b_{23}^{l}\approx 82.1211>0,\\ r_{3}^{m}+a_{34}^{m}M_{4}-k_{3}^{l}n_{3}\approx 10.6416>0,\;r_{4}^{m}+a_{43}^{m}M_{3}-k_{4}^{l}n_{4}\approx 11.1391>0,\\ a_{31}^{l}m_{1}-M_{1}(r_{3}^{m}+a_{34}^{m}M_{4}-k_{3}^{l}n_{3})\approx 7.5122>0,\;\\ a_{41}^{l}m_{1}-M_{1}(r_{4}^{m}+a_{43}^{m}M_{3}-k_{4}^{l}n_{4})\approx 8.0712>0, \end{array}$$

$$\begin{array}{ll} A_{1} & =a_{11}^{l}-a_{21}^{m}-q_{1}^{m}-(a_{11}^{m}\tau_{11}+a_{21}^{m}\tau_{21})[r_{1}^{m}+a_{11}^{m}M_{1}+a_{12}^{m}M_{2}+\frac{a_{13}^{m}M_{3}}{b_{13}^{l}m_{3}+m_{1}}\\ & \;\;+\frac{a_{14}^{m}M_{4}}{b_{14}^{l}m_{4}+m_{1}}+k_{1}^{m}N_{1}]-(M_{1}a_{11}^{m}\tau_{11}+M_{1}a_{21}^{m}\tau_{21}+1+b_{13}^{m})\frac{a_{13}^{m}M_{3}}{{\left(b_{13}^{l}m_{3}+m_{1}\right)}^{2}}\\ & \;\;-(M_{1}a_{11}^{m}\tau_{11}+M_{1}a_{21}^{m}\tau_{21}+1+b_{14}^{m})\frac{a_{14}^{m}M_{4}}{{\left(b_{14}^{l}m_{4}+m_{1}\right)}^{2}}-M_{1}{\left(a_{11}^{m}\right)}^{2}\tau_{11}-M_{2}a_{21}^{m}a_{12}^{m}\tau_{12}\\ & \;\;-M_{2}a_{21}^{m}a_{22}^{m}\tau_{22}-M_{1}a_{21}^{m}a_{11}^{m}\tau_{21}-\frac{a_{34}^{m}\tau_{34}a_{41}^{m}b_{14}^{m}{\left(M_{4}\right)}^{2}}{{\left(b_{14}^{l}m_{4}+m_{1}\right)}^{2}}-\frac{a_{43}^{m}\tau_{43}a_{31}^{m}b_{13}^{m}{\left(M_{3}\right)}^{2}}{{\left(b_{13}^{l}m_{3}+m_{1}\right)}^{2}}\\ & \approx 7.8508, \end{array}$$

$$\begin{array}{ll} A_{2} & =a_{22}^{l}-a_{12}^{m}-q_{2}^{m}-(a_{12}^{m}\tau_{12}+a_{22}^{m}\tau_{22})[r_{2}^{m}+a_{22}^{m}M_{2}+a_{21}^{m}M_{1}+\frac{a_{23}^{m}M_{3}}{b_{23}^{l}m_{3}+m_{2}}\\ & \;\;+\frac{a_{24}^{m}M_{4}}{b_{24}^{l}m_{4}+m_{2}}+k_{2}^{m}N_{2}]-(M_{2}a_{12}^{m}\tau_{12}+M_{2}a_{22}^{m}\tau_{22}+1+b_{23}^{m})\frac{a_{23}^{m}M_{3}}{{\left(b_{23}^{l}m_{3}+m_{2}\right)}^{2}}\\ & \;\;-(M_{2}a_{12}^{m}\tau_{12}+M_{2}a_{22}^{m}\tau_{22}+1+b_{24}^{m})\frac{a_{24}^{m}M_{4}}{{\left(b_{24}^{l}m_{4}+m_{2}\right)}^{2}})-M_{1}a_{12}^{m}a_{11}^{m}\tau_{11}-M_{2}a_{12}^{m}a_{22}^{m}\tau_{12}\\ & \;\;-M_{2}{\left(a_{22}^{m}\right)}^{2}\tau_{22}-M_{1}a_{12}^{m}a_{21}^{m}\tau_{21}-\frac{a_{34}^{m}\tau_{34}a_{42}^{m}b_{24}^{m}{\left(M_{4}\right)}^{2}}{{\left(b_{24}^{l}m_{4}+m_{2}\right)}^{2}}-\frac{a_{43}^{m}\tau_{43}a_{32}^{m}b_{23}^{m}{\left(M_{3}\right)}^{2}}{{\left(b_{23}^{l}m_{3}+m_{2}\right)}^{2}}\\ & \approx 7.7023, \end{array}$$

$$\begin{array}{ll} A_{3} & =\frac{a_{31}^{l}b_{13}^{l}m_{2}}{{\left(b_{13}^{m}M_{3}+M_{1}\right)}^{2}}+\frac{a_{32}^{l}b_{23}^{l}m_{2}}{{\left(b_{23}^{m}M_{3}+M_{2}\right)}^{2}}-a_{34}^{m}\tau_{34}a_{43}^{m}M_{4}-a_{43}^{m}-q_{3}^{m}\\ & \;-\frac{a_{13}^{m}M_{1}+a_{13}^{m}{\left(M_{1}\right)}^{2}(a_{11}^{m}\tau_{11}+a_{21}^{m}\tau_{21})+a_{43}^{m}\tau_{43}a_{31}^{m}b_{13}^{m}M_{1}M_{3}}{{\left(b_{13}^{l}m_{3}+m_{1}\right)}^{2}}\\ & \;\;-\frac{a_{23}^{m}{\left(M_{2}\right)}^{2}a_{12}^{m}\tau_{12}+a_{43}^{m}\tau_{43}a_{32}^{m}b_{23}^{m}M_{2}M_{3}+a_{23}^{m}M_{2}+a_{23}^{m}{\left(M_{2}\right)}^{2}a_{22}^{m}\tau_{22}}{{\left(b_{23}^{l}m_{3}+m_{2}\right)}^{2}}\\ & \;\;-a_{43}^{m}\tau_{43}[r_{3}^{m}+\frac{a_{31}^{m}M_{1}}{b_{13}^{l}m_{3}+m_{1}}+\frac{a_{32}^{m}M_{2}}{b_{23}^{l}m_{3}+m_{2}}+a_{34}^{m}M_{4}+k_{3}^{m}N_{3}^{m}]\\ & \approx 1.4256, \end{array}$$

$$\begin{array}{ll} A_{4} & =\frac{a_{41}^{l}b_{14}^{l}m_{2}}{{\left(b_{14}^{m}M_{4}+M_{1}\right)}^{2}}+\frac{a_{42}^{l}b_{24}^{l}m_{2}}{{\left(b_{24}^{m}M_{4}+M_{2}\right)}^{2}}-a_{34}^{m}-a_{43}^{m}\tau_{43}a_{34}^{m}M_{3}-q_{4}^{m}\\ & \;\;-\frac{a_{14}^{m}M_{1}+a_{14}^{m}{\left(M_{1}\right)}^{2}a_{11}^{m}\tau_{11}+a_{14}^{m}{\left(M_{1}\right)}^{2}a_{21}^{m}\tau_{21}+a_{34}^{m}\tau_{34}a_{41}^{m}b_{14}^{m}M_{1}M_{4}}{{\left(b_{14}^{l}m_{4}+m_{1}\right)}^{2}}\\ & \;\;-\frac{a_{24}^{m}{\left(M_{2}\right)}^{2}(a_{12}^{m}\tau_{12}+a_{22}^{m}\tau_{22})+a_{34}^{m}\tau_{34}a_{42}^{m}b_{24}^{m}M_{2}M_{4}+a_{24}^{m}M_{2}}{{\left(b_{24}^{l}m_{4}+m_{2}\right)}^{2}}\\ & \;\;-a_{34}^{m}\tau_{34}[r_{4}^{m}+\frac{a_{41}^{m}M_{1}}{b_{14}^{l}m_{4}+m_{1}}+\frac{a_{42}^{m}M_{2}}{b_{24}^{l}m_{4}+m_{2}}+a_{43}^{m}M_{3}+k_{4}^{m}N_{4}^{m}]\\ & \approx 1.4528, \end{array}$$

$$B_{1}=f_{1}^{l}-k_{1}^{m}(1+M_{1}a_{11}^{m}\tau_{11})-M_{1}k_{1}^{m}a_{21}^{m}\tau_{21}\approx 9.4382,$$

$$B_{2}=f_{2}^{l}-M_{2}k_{2}^{m}a_{12}^{m}\tau_{12}-k_{2}^{m}(1+M_{2}a_{22}^{m}\tau_{22})\approx 8.9257,$$

$$B_{3}=f_{3}^{l}-k_{3}^{m}-a_{43}^{m}\tau_{43}k_{3}^{m}M_{3}\approx 8.9400,$$

and

$$B_{4}=f_{4}^{l}-k_{4}^{m}-a_{34}^{m}\tau_{34}k_{4}^{m}M_{4}\approx 9.4500.$$

In light of the previously obtained computational results, it is apparent that the models (63)–(64) satisfy the conditions $\left(H_{1})-(H_{18}\right)$ stipulated in Theorem 4. As is consistent with the assertions of Theorem 4, these models exhibit a unique, strictly positive, 2-periodic SHPS, denoted by $\left(v_{1}^{*}(t),v_{2}^{*}(t),v_{3}^{*}(t),v_{4}^{*}(t),u_{1}^{*}(t),u_{2}^{*}(t),u_{3}^{*}(t),u_{4}^{*}(t)\right)$, which additionally fulfills

$$\underset{t\to +\infty }{\lim\;}\left|v_{i}(x,t)-v_{i}^{*}(t)\right|=0,\;\underset{t\to +\infty }{\lim\;}\left|u_{i}(x,t)-u_{i}^{*}(t)\right|=0,\;i=1,2,3,4,\;uniformly\;for\;x\in [0,2\pi ].$$

**Figure 1:**
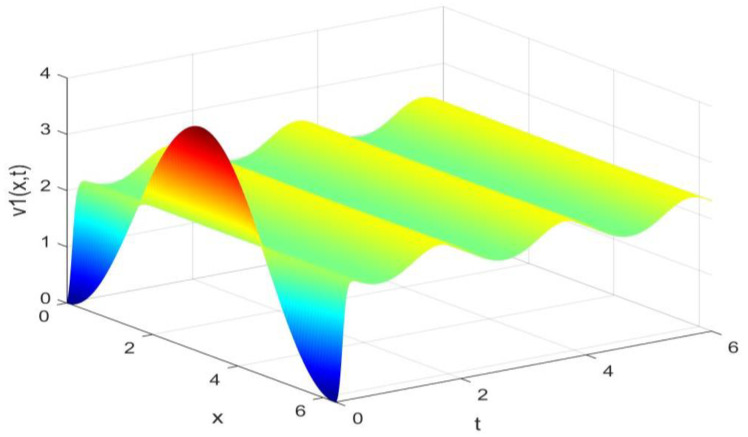
The evolution process of the density for the species $v_{1}(x,t)$ of models (63) and (64).

**Figure 2:**
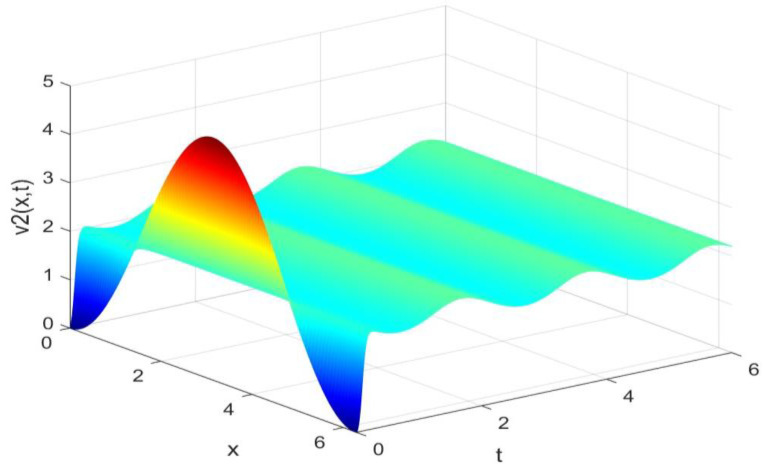
The evolution process of the density for the species $v_{2}(x,t)$ of models (63) and (64).

**Figure 3:**
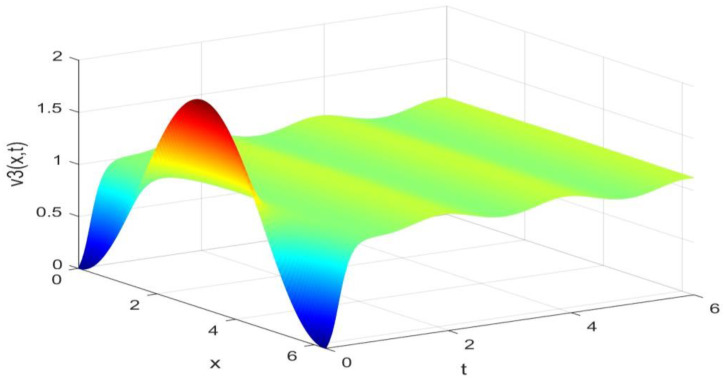
The evolution process of the density for the species $v_{3}(x,t)$ of models (63) and (64).

**Figure 4:**
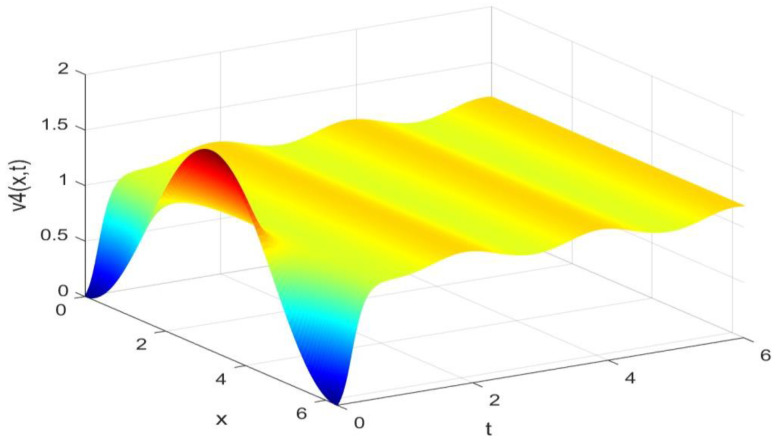
The evolution process of the density for the species $v_{4}(x,t)$ of models (63) and (64).

**Figure 5:**
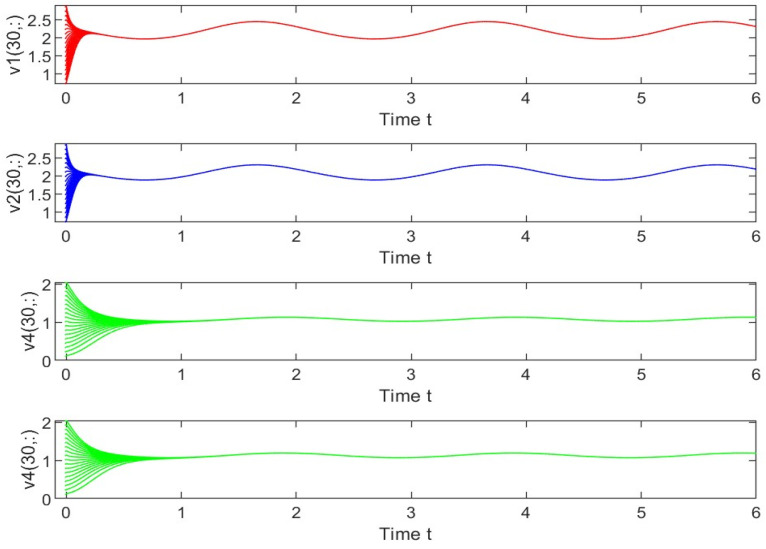
As the spatial variable x=0.6π, the changing patterns of population densities in models (63) and (64) are caused by different positive initial values.

**Figure 6:**
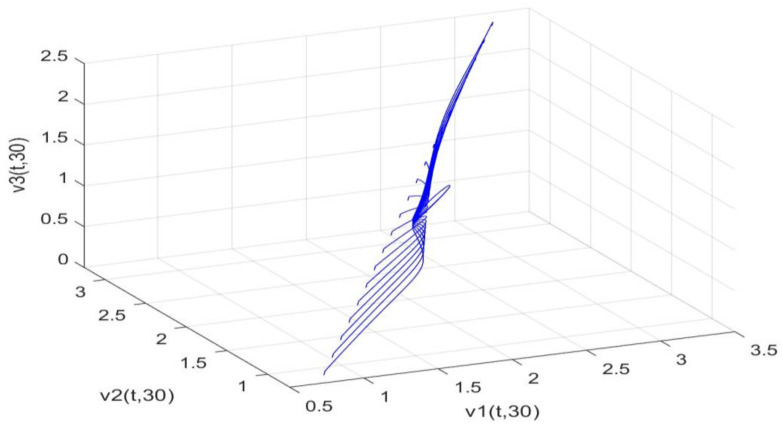
As the spatial variable x=0.6π, the changing patterns of population densities. $v_{1},v_{2},v_{3}$ in models (63) and (64) are caused by different positive initial values.

**Figure 7:**
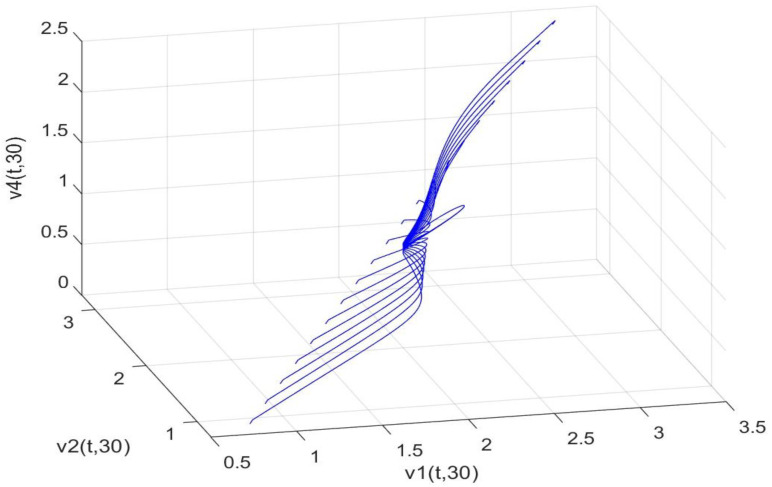
As the spatial variable x=0.6π, the changing patterns of population densities $v_{1},v_{2},v_{4}$ in models (63) and (64) are caused by different positive initial values.

**Figure 8:**
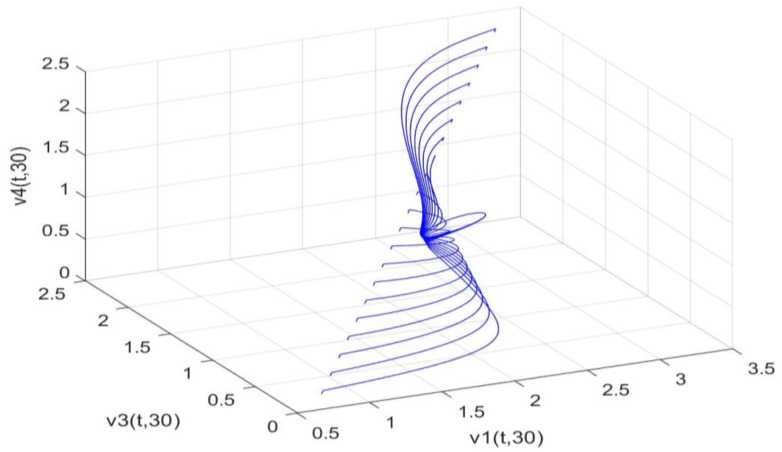
As the spatial variable x=0.6π, the changing patterns of population densities $v_{1},v_{3},v_{4}$ in models (63) and (64) are caused by different positive initial values.

**Figure 9:**
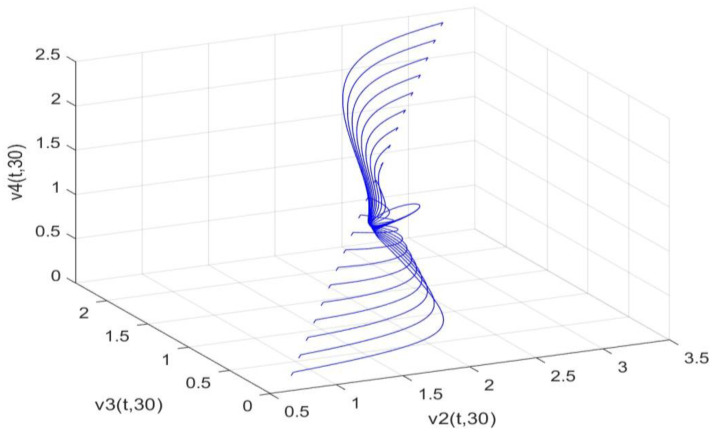
As the spatial variable x=0.6π, the changing patterns of population densities $v_{2},v_{3},v_{4}$ in models (63) and (64) are caused by different positive initial values.

**Figure 10:**
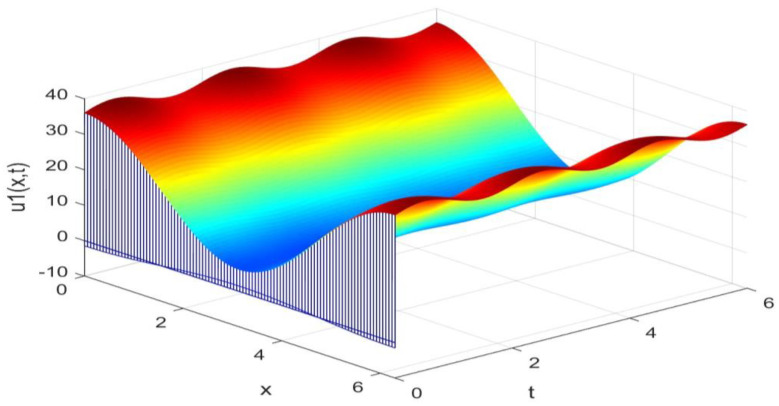
The variation patterns of the values of the control function $u_{1}(x,t)$.

**Figure 11:**
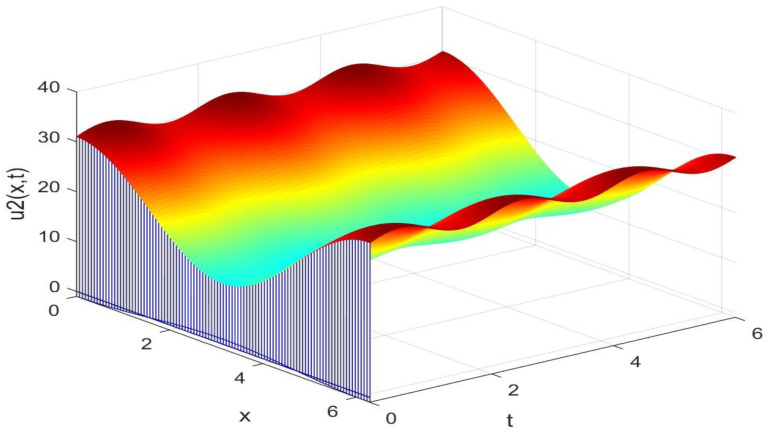
The variation patterns of the values of the control function $u_{2}(x,t)$.

**Figure 12:**
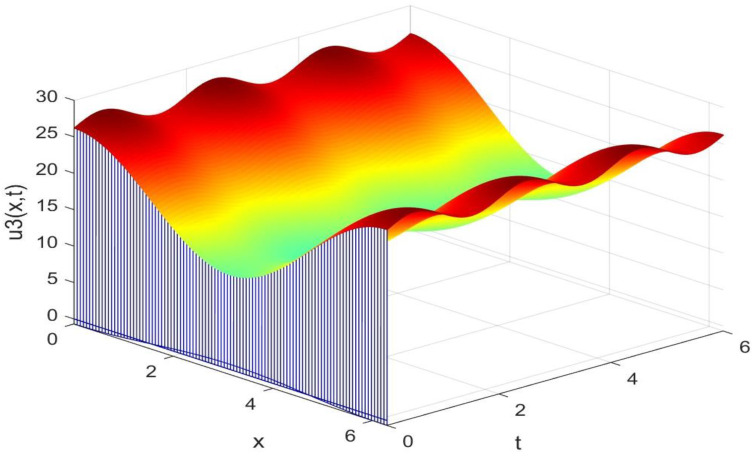
The variation patterns of the values of the control function $u_{3}(x,t)$.

**Figure 13:**
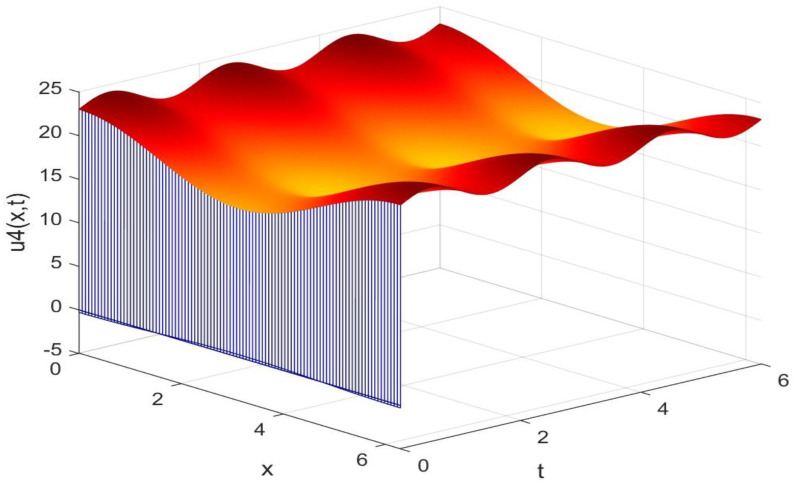
The variation patterns of the values of the control function $u_{4}(x,t)$.

Utilizing the finite difference method and MATLAB R2023a, we can obtain numerical solutions for the model (63) and (64), which are illustrated in Figure 1, Figure 2, Figure 3 and Figure 4. An inspection of Figure 1, Figure 2, Figure 3 and Figure 4 reveals that the system defined by (63) and (64) possesses a strictly positive SHPS. Within the framework of models (63) and (64), the populations of both prey and predators undergo periodic fluctuations with a cycle of 2, ultimately converging to above SHPS as time advances sufficiently. To confirm the global asymptotic stability of the aforementioned SHPS for models (63) and (64), we conducted extensive numerical simulations using diverse positive initial values. The results consistently demonstrated that, regardless of the positive initial condition chosen, the 2-periodic solution of the models (63) and (64) is asymptotically stable. For further details, please refer to Figure 5, Figure 6, Figure 7, Figure 8 and Figure 9. Additionally, Figure 10, Figure 11, Figure 12 and Figure 13 illustrate the variation patterns of the control function’s values.

The theoretical analysis presented in this article elucidates the dynamic stability mechanisms underlying the predator–prey system. When the prey birth rate and predators’ post-predation nutrient absorption rates reach sufficient levels, the four species within the predator–prey system can sustain long-term, stable coexistence, thereby effectively mitigating the risk of population extinction (as outlined in Theorem 2). Even more fascinating is the observation that, upon fulfilling these fundamental conditions, if additional key parameters such as population diffusion rates, interaction intensities, predators’ natural mortality rates, and predator-to-prey numerical ratios also meet specific thresholds, then, under minor temporal delays, the species densities within the predator–prey system will exhibit periodic fluctuations. This further reveals the intricate beauty of dynamic equilibrium within ecosystems (as demonstrated in Theorem 4). These theoretical findings have been rigorously validated through precise numerical simulations, providing a solid theoretical foundation for understanding, assessing, and preserving ecosystem balance. Specifically, the conditions are formulated as a set of inequalities, rather than stringent equations, thereby offering significant ease and flexibility in the application of these theoretical insights to practical ecosystem management scenarios.

## 5. Conclusions and Future Research Directions

With the advancement of mathematical theories and computational capabilities, research on multi-species periodic reaction–diffusion predator–prey models incorporating time delays and feedback control has garnered increasing attention. This article not only considers time delay effects and feedback control but also takes into account ratio-dependent interactions between predators and prey. Furthermore, it emphasizes the external influences of environmental factors (such as climate change and seasonal resource fluctuations) on population dynamics, which often vary over time, rendering the system non-closed or non-self-sufficient and aligning the model more closely with real-world ecological environments. Additionally, this study innovatively transforms the complex stability problem of time-delayed reaction–diffusion ecological systems into the corresponding stability problem of time-delayed ordinary differential ecological systems, enabling the utilization of well-established stability theories for ordinary differential equations for analysis. This transformation not only simplifies the research process and enhances efficiency but also provides a new perspective and methodology for dealing with similar complex systems.

Despite significant progress in this study, it primarily focuses on integer-order partial differential ecological systems. Given that complex dynamic processes in ecological systems often involve fractional-order derivatives, which can more finely capture nuanced patterns of interspecies interactions, such as the long-term impact of environmental factor accumulation on population size, future research will shift towards fractional-order time-delayed reaction–diffusion ecological systems. This shift aims to comprehensively unveil the dynamic characteristics of this domain, thereby offering more precise theoretical guidance and decision-making support for ecosystem management and biodiversity conservation. The stability of periodic solutions in diffusive ecological systems with variable or continuous time delays, incorporating feedback control, is also an important topic worthy of further investigation.

## Figures and Tables

**Table 1:** The biological implications associated with the parameters in the models (1).

Parameter	Definition	Parameter	Definition
$d_{i}(t),(i=1,2,3,4)$	The diffusivity rates	$a_{ij}(t),(i=1,2,j=3,4)$	The capturingrates of the predators
$r_{i}(t),(i=1,2)$	The intrinsic growth rate	$a_{\mathrm{ii}}(t),(i=1,2)$	The interaction withinprey species
$r_{i}(t),(j=1,2)$	The death growth rate	$a_{12}(t),a_{21}(t),a_{34}(t),a_{43}(t)$	The interference between two species
$a_{ij}(t),(i=3,4,j=1,2)$	The conversion rates	$b_{ij}(t),(i=1,2,j=3,4)$	The interference within predator species

## Data Availability

Data are contained within the article.
